# The Role of PDE11A4 in Social Isolation-Induced Changes in Intracellular Signaling and Neuroinflammation

**DOI:** 10.3389/fphar.2021.749628

**Published:** 2021-11-23

**Authors:** Katy Pilarzyk, Reagan Farmer, Latarsha Porcher, Michy P. Kelly

**Affiliations:** ^1^ Department of Pharmacology, Physiology and Neuroscience, University of South Carolina School of Medicine, Columbia, SC, United States; ^2^ Department of Anatomy and Neurobiology, University of Maryland School of Medicine, Baltimore, MD, United States; ^3^ Center for Aging Research, University of Maryland School of Medicine, Baltimore, MD, United States

**Keywords:** phosphodiesterase, PDE11, social isolation, neuroinflammation, social memory, subcellular compartmentalization, ventral hippocampus, PDE10

## Abstract

Phosphodiesterase 11A (PDE11A), an enzyme that degrades cyclic nucleotides (cAMP and cGMP), is the only PDE whose mRNA expression in brain is restricted to the hippocampal formation. Previously, we showed that chronic social isolation changes subsequent social behaviors in adult mice by reducing expression of PDE11A4 in the membrane fraction of the ventral hippocampus (VHIPP). Here we seek extend these findings by determining 1) if isolation-induced decreases in PDE11A4 require chronic social isolation or if they occur acutely and are sustained long-term, 2) if isolation-induced decreases occur uniquely in adults (i.e., not adolescents), and 3) how the loss of PDE11 signaling may increase neuroinflammation. Both acute and chronic social isolation decrease PDE11A4 expression in adult but not adolescent mice. This decrease in PDE11A4 is specific to the membrane compartment of the VHIPP, as it occurs neither in the soluble nor nuclear fractions of the VHIPP nor in any compartment of the dorsal HIPP. The effect of social isolation on membrane PDE11A4 is also selective in that PDE2A and PDE10A expression remain unchanged. Isolation-induced decreases in PDE11A4 expression appear to be functional as social isolation elicited changes in PDE11A-relevant signal transduction cascades (i.e., decreased pCamKIIα and pS6-235/236) and behavior (i.e., increased remote long-term memory for social odor recognition). Interestingly, we found that isolation-induced decreases in membrane PDE11A4 correlated with increased expression of interleukin-6 (IL-6) in the soluble fraction, suggesting pro-inflammatory signaling for this cytokine. This effect on IL-6 is consistent with the fact that PDE11A deletion increased microglia activation, although it left astrocytes unchanged. Together, these data suggest that isolation-induced decreases in PDE11A4 may alter subsequent social behavior via increased neuroinflammatory processes in adult mice.

## Introduction

Various phosphodiesterase (PDE) families are known to influence social behaviors (Sano et al., 2008; Grauer et al., 2009a; Alexander et al., 2016; Gurney et al., 2017; Maurin et al., 2018; Enomoto et al., 2019) or neuroinflammation (Sebastiani et al., 2006; Peixoto et al., 2015; Schwenkgrub et al., 2017; Tibbo and Baillie, 2020; Ponsaerts et al., 2021); but evidence suggests the PDE11A family may be a key regulator of both. Although there are four isoforms of PDE11A, PDE11A4 is the longest and only isoform found in the brain (Yuasa et al., 2001a; Yuasa et al., 2001b). PDE11A4 exists as a homodimer and degrades both 3′,5′-cyclic adenosine monophosphate (cAMP) and 3′,5′-cyclic guanosine monophosphate (cGMP) (Kelly, 2015) and is particularly enriched in cell bodies, dendrites and axons of neurons of the superficial layer of CA1, the subiculum, and the adjacently connected amygdalohippocampal area of the ventral hippocampal formation (VHIPP; aka anterior hippocampal formation in primates) (Kelly et al., 2010; Hegde et al., 2016a). Low levels of PDE11A4 expression can be found in neurons of the dorsal hippocampus (DHIPP), dorsal root ganglion, and spinal cord, but no protein expression has been found to originate from glia, other brain regions, or over 20 peripheral organs (Kelly et al., 2010; Kelly, 2015; Hegde et al., 2016a; Pathak et al., 2017; Kelly, 2018b). Interestingly, expression of PDE11A4 in the hippocampus dramatically increases over the lifespan, suggesting PDE11A function may evolve with age (Kelly et al., 2014; Hegde et al., 2016a). Of relevance to the current study, several findings point to VHIPP as a key node in the neurocircuitry underlying social behaviors, including studies employing lesions, optogenetic stimulation, or manipulation of the cAMP cascade (Brightwell et al., 2005; Bickle, 2008; Tseng et al., 2009; Okuyama et al., 2016). Interestingly, PDE11A4-expressing neurons in the VHIPP project to the nucleus accumbens (i.e., a brain region key in motivation and reward) (Cembrowski et al., 2016), and possibly the prefrontal cortex (Pilarzyk et al., 2019), but not the amygdala (i.e., a brain region key to emotion) (Cembrowski et al., 2016). While little has been characterized of the signaling pathways lying up or downstream of PDE11A4, we have shown that PDE11A4 regulates glutamatergic and calcium/calmodulin-dependent kinase II (CamKII) signaling as well as protein synthesis (Kelly et al., 2010; Kelly et al., 2014; Hegde et al., 2016a; Pilarzyk et al., 2019). Further, deleting PDE11A triggers changes in the oxytocin signaling pathway, a pathway crucial in regulating social behaviors (Hegde et al., 2016b). Thus, it may not be surprising that PDE11A4 is proving to be a key regulator of social memory and social interactions (Kelly et al., 2010; Hegde et al., 2016a; Hegde et al., 2016b; Pilarzyk et al., 2019; Smith et al., 2021).

Social experiences sculpt subsequent social behaviors, and PDE11A4 appears to be a critical neurobiological mechanism regulating this feedforward cycle. Upon biochemical fractionation of the hippocampus, about half of PDE11A4 is found in the soluble fraction, with the other half split between the nuclear and membrane fractions (Kelly, 2018b). We find that it is membrane PDE11A4 that is particularly important in regulating social behaviors in both young adult and old mice (Hegde et al., 2016b; Smith et al., 2021). Previously, we showed that chronic social isolation in adult mice decreases PDE11A4 expression specifically within the membrane compartment of the VHIPP, and that this isolation-induced decrease was sufficient to alter subsequent social interactions and social memory in a PDE11A genotype-dependent manner (Hegde et al., 2016b). Importantly, deletion of PDE11A mimicked these behavioral effects of social isolation, and we found no further effect of social isolation on these social behaviors in *Pde11a* KO mice. Together, this suggests that the isolation-induced decreases in membrane-associated PDE11A4 fully accounts for the ability of social isolation to alter subsequent social behavior. In support of this finding, expressing an isolated PDE11A GAF-B domain to disrupt PDE11A4 homodimerization, which selectively degrades membrane-associated PDE11A4 (Pathak et al., 2017), was sufficient to alters social preference behavior (Smith et al., 2021). In a similar vein, both group-housed and single-housed *Pde11a* KO mice showed equivalent antidepressant-like effects of chronic lithium; whereas, only single-housed *Pde11a* WT mice showed this antidepressant-like effect (Pathak et al., 2017). This suggests that either genetic deletion or isolation-induced reductions in PDE11A4 are sufficient to drive increased lithium responsivity. That said, social isolation can impact other non-social phenotypes in *Pde11a* KO mice. For example, single-housed *Pde11a* KO mice show hyperactivity in an open field but normal prepulse inhibition (PPI) (Kelly et al., 2010), while group-housed *Pde11a* KO mice show no hyperactivity but slightly reduced PPI (Kelly, 2017). Together, these data suggest that the isolation-induced decreases in PDE11A4 described herein mediate some—but not all—phenotypes caused by social isolation.

Several lines of evidence suggest neuroinflammation may be a critical link between PDE11A, social isolation, and subsequent changes in social behaviors. As noted above, we previously found that isolation-induced decreases in membrane PDE11A occur following chronic social isolation in adult mice (Hegde et al., 2016b). Interestingly, mice with lower levels of membrane PDE11A4 express significantly higher levels of soluble interleukin 6 (IL-6) (Pathak et al., 2017), a cytokine that is upregulated in humans and rodents by social isolation (Karelina et al., 2009; Alshammari et al., 2020) and that negatively affects social behaviors (Wei et al., 2012a). The fact that deletion of PDE11A increased IL-6 expression in the DHIPP (Pathak et al., 2017) suggests isolation-induced decreases in membrane PDE11A signaling may drive a compartment-specific increase in IL-6 (i.e., in the soluble compartment), at least in the adult brain. Literature suggests the adolescent brain is generally more sensitive to the effects of social isolation than is the adult brain (Lukkes et al., 2009; Burke et al., 2017; Arakawa, 2018; Walker et al., 2019). That said, hippocampal PDE11A4 expression is extremely low during early postnatal development (Hegde et al., 2016a) and the effect of PDE11A deletion on social behaviors during adolescence can differ from that during adulthood (Hegde et al., 2016b). This may suggest that social isolation may not impact brain function via PDE11A in the adolescent brain as it does in the adult brain. Therefore, we seek to test the hypothesis that specifically during adulthood, isolation-induced decreases in PDE11A alter subsequent social behaviors by regulating neuroinflammation in the hippocampus. To do so, we determined 1) whether social isolation-induced decreases in PDE11A4 expression occur acutely and are sustained long term or whether they only manifest as a result of long-term social isolation, 2) if isolation decreases PDE11A4 only in adult brains (i.e., not adolescent brains), and 3) if social isolation and loss of PDE11A signaling similarly upregulate markers of neuroinflammation. We found that in adults, but not adolescent mice, 1) both acute and chronic social isolation selectively decrease PDE11A4 in the VHIPP membrane and 2) both isolation and loss of PDE11A signaling increase IL-6 in the soluble fraction, possibly due to increased activation of microglia.

## Materials and Methods

*Subjects.* Adult C57BL/6J mice were obtained from Jackson Laboratories at 9 weeks of age and were group-housed for at least 1 week to habituate to the facility (3/cage). After habituation, the adult C57BL/6J mice continued to be group-housed (GH) or were single-housed for 1 month (SM), 1 week (SW), 1 day (SD), or 1 h (SH) prior to being sacrificed. It was anecdotally noted that the mice that were isolated for only 1 h were more far more active in their cages relative to the other groups at the time of sacrifice, likely due to the more recent handling and/or cage change. The other groups were not similarly moved to new cages 1 h before sacrifice to be able to compare results here with those we obtained previously (Hegde et al., 2016b). All adult groups were sacrificed at ∼3 months of age. Adolescent C57BL6/J mice arrived at facility on post-natal day 21. The adolescent mice were allowed 1 week to habituate while group-housed, after which they either remained group-housed (GH) or were single-housed for 1 month (SM) prior to sacrifice at 8 weeks of age. *Pde11a* wildtype (WT) and knockout (KO) mice were originally developed by Deltagen (San Mateo, CA, United States), and are maintained on a mixed C57BL/6J (86.6%)-C57BL/6N (12.4%)-129S6 (1%) background (confirmed by Transnetyx genetic monitoring service, Cordova TN; (Kelly et al., 2010). *Pde11a* mice were bred onsite in heterozygous (HT) × HT matings, with same-sex wild-type (WT), HT, and KO littermates weaned together into the same cage at P28 (3–5/cage) and aged into adulthood. Mice used in the biochemical fractionation experiment were 2–6 months old; whereas, mice used in the proteomics study were 18–24 months old. All mice were housed on a 12 h:12 h light:dark cycle and given food and water ad libitum. See Figure legends for experimental n’s. Experiments were performed in accordance with the National Institutes of Health Guide (Pub 85-23, revised 1996) as well as approved by the Institutional Animal Care and Use Committee of the University of South Carolina.

*Western Blotting.* Animals were sacrificed by cervical dislocation, and the brain regions of interest were then dissected out and placed into prelabeled tubes on dry ice. Samples were stored at −80 C until processed. Each sample contained tissue from three mice to provide sufficient starting material for biochemical fractionation. Tissue samples were homogenized and biochemically fractionated as previously described (Pathak et al., 2017; Patel et al., 2018). Lysis buffer was prepared (20 mM Tris-HCl, pH 7.5; 2 mM MgCl_2;_ Thermo Pierce Scientific phosphatase tablet #A32959 and protease inhibitor 3 #P0044) and kept on wet ice. Once the buffer was added to frozen tissue, the sample was quickly homogenized using a probe sonicator. Aliquots of total homogenates were removed and set aside. The remaining sample was spun in a centrifuge held at 4°C at 1,000 g × 10 min to obtain nuclear pellet and supernatant containing both soluble and membrane fractions. This supernatant was then placed in a new Beckman Centrifuge tube and spun at 89,000 rcf x 1 h at 4°C. This resulted in producing a membrane pellet and a supernatant which contains the soluble fraction. Following the removal of the soluble fraction supernatant, the membrane pellet was washed and resuspend in fresh buffer and the 1-h spin was repeated. Triton X-100 (0.5%) was added to the lysis buffer, and the membrane pellet was resuspended in this buffer by sonication. The sample was nutated for 30 min at 4°C. The soluble membrane was separated from the insoluble membrane by centrifugation at 60,000 rcf × 30 min at 4°C and the supernatant was collected as the soluble membrane. It has been reported that PDE11A is expressed in both soluble and insoluble membrane (Bergeron et al., 2017; Kelly, 2017), but we have found a high degree of background with our antibody when probing insoluble membrane fractions (Kelly, 2018a). Therefore, insoluble membrane fractions were not tested here. All fractions were kept at −80°C until use. Protein expression was assessed by Western blotting as previously described (Kelly et al., 2010; Kelly et al., 2014). Invitrogen 4–12% Bis-Tris gels (Life Technologies, NP0323BOX) were used to separate proteins using electrophoresis. Following electrophoresis, the gels were set to transfer to nitrocellulose membrane (GE Healthcare Life Sciences, 10600008). As previously described (Porcher et al., 2021; Smith et al., 2021), nitrocellulose membranes were cut in order to probe for multiple antibodies at different molecular weights. We have found this is preferred over stripping and reprobing the same membrane as antibody binding specificity can differ between fresh verus stripped blots. We have also found that the smaller size of cut membranes allows for improved movement in the trays during washes and improves signal: noise. Cut membranes were then probed with the following antibodies: PDE11A–112 (Fabgennix, Custom; 1:500), S6 (Cell Signaling 2217S; 1:200), pS6–235/236 (Cell Signaling 4856S; 1:1000), pS6–240/244 (Cell Signaling 4838S; 1:2500), Interleukin-6 (Life Technologies, ARC0962; 1:2500), CamKIIα/CamKIIβ (Cell Signaling, 3362; 1:20,000), pCamKIIα/pCamKIIβ (Cell Signaling, 3361S; 1:1000), and Actin (Sigma-Aldrich, A2066; 1:10 000) as a loading control. Secondary antibodies were also applied: rabbit IgG (Jackson Immunoresearch, 111-035-144; 1:10 000), mouse IgG (Jackson Immunoresearch, 115-035-146; 1:10 000). Biochemically fractionated *Pde11a* wildtype (WT) and knockout (KO) hippocampal samples were run on PDE11A blots as a positive and negative control, respectively, for the PDE11A4 antibody. Cerebellum from a WT was run as a negative control for the PDE2A antibody and the striatum from a WT was run as a positive control for the PDE10A antibody. Multiple film exposures were taken to ensure data were collected within the linear range. Western blots were quantified using ImageJ (NIH). Unadjusted images of uncropped blot can be found in Supplementary Data 1.

*Odor recognition*. Adult C57BL/6J mice were tested in social odor recognition (SOR) and non-social odor recognition (NSOR) as previously described (Hegde et al., 2016a; Hegde et al., 2016b; Pilarzyk et al., 2019). Mice were allowed at least 1 week to habituate to one-inch round wooden beads (Woodworks) placed in their home-cages, during which the beads become scented with the home cage odor. During the assay, the mice are placed in individual cages and first presented three home-cage beads during a 3-min habituation trial. Then, each mouse received two 3-min training periods (with 5 min between trials) where they are presented with two home-cage beads and one novel bead from another mouse strain (BALB/cJ, JAX #00065; 129S6/SvEv, Taconic #129SVE). For NSOR, instead of beads scented with other mouse strains, they are scented with bedding mixed with a household spice (basil, ginger, or thyme). Either 24 h or 7 days after training, recent-long term and remote-long term memory were assessed, respectively. During the 2-min testing trials for both SOR and NSOR, subjects were presented with one home-cage bead, the bead they received during training (i.e., familiar odor), and one novel bead (i.e., novel odor). Social and non-social odors presented as novel/familiar were counterbalanced to eliminate any potential preference bias and to verify that memory phenotypes were not due to one scent. An investigator blind to housing condition and bead recorded the amount of time spent (in seconds) investigating each bead. Mice are disqualified from analysis if they do not spend at least 3 s investigating the beads; however, no animals were disqualified here on this criterion. Preference ratio was calculated by (novel-familiar)/(novel + familiar) for statistical analysis. Spending significantly more time exploring the novel versus familiar scent was considered odor recognition memory.

*Immunofluorescence (IF) and Immunohistochemistry (IHC)*. *Pde11a* WT and KO brains were processed together by embedding in matrix (Epredia M-1 Embedding Matrix 1310, Thermofisher; 10056778) to form a block, cryosectioned in the sagittal plane at 20 μm, and thaw-mounted onto the same slide. Slides were briefly dried at room temperature before being stored at −80 C until processing. Slides were fixed in 4% paraformaldehyde for 20 min at room temperature and then washed 3 times for 1 minute in phosphate-buffered saline (PBS). Slides were blocked with PBS/0.4% BSA/0.3% Triton-for three rounds consisting of 10 min each. Slides were probed overnight at 4 C with an antibody against IBA-1 (Wako-Chemicals, 019-1974; 1:1000) or GFAP (Cell Signaling, 12389P; 1:500) diluted in PBT. The next day, sections were washed 4 × 10 min in PBT. To detect protein expression, immunofluorescence slides were probed with a secondary antibody for 90 min at room temperature, Alexafluor-488 anti-rabbit (Jackson Immunoresearch, 711-545-152; 1:400), and IHC slides were probed with a biotinylated anti-rabbit secondary (Vector Laboratories, BA-1000). Slides were then washed for three rounds of 10 min each in PBT and then rinsed in PBS by quickly dipping in solution 10 times to remove any detergent. Immunofluorescent sections were mounted with a coverslip and DAPI (Southern Biotech, 0100-20). IHC sections were Incubated with ABC (Vector Laboratories, PK-6100) reagent for 90 min at room temperature followed by 3 × 10 min washes with PBS. The detection reagent DAB (Vector Laboratories, SK-4100) was then applied and allowed to develop. The DAB detection was then stopped by dipping the slide in PBS. Slides were then mounted for viewing with Permount mounting media (Fisher, SP15-100) after a brief drying period. Images of hippocampi were taken with MBF Bioscience CX9000 camera and Neurolucida software (MBF Bioscience, Willston, VT). An experimenter blind to genotype collected the microglia and astrocyte data (# cells and intensity of staining/cell) using ImageJ (NIH), being sure to not count cells intersecting with the upper or left sides for stereological best practice.

*2-dimensional gel electrophoresis (2DGE) and mass spectroscopy (MS).* Ventral hippocampi from four cohorts of *Pde11a* WT and KO mice were harvested and homogenized as described above for Western blotting. Lysates for all WTs (Cohort 1: 2F + 3M; Cohort 2: 3M; Cohort 3: 4F; Cohort 4: 6M), and all KOs (Cohort 1: 2F + 3M; Cohort 2: 3M; Cohort 3: 4F; Cohort 4: 6M) within a cohort were then pooled to allow for sufficient starting material. 2DGE profiling were performed by Applied Biomics, Inc. (Hayward, CA), with the WT and KO sample from a given cohort run on the same gel. Importantly, the pooled WT and pooled KO sample from each cohort were labelled with 1 of 2 CyDyes in a counterbalanced manner (i.e., 2 gels labelled the WT sample with Dye1 and KO with Dye2 and 2 gels labelled the KO sample with Dye1 and the WT with Dye 2). Spots differing in expression with FDR-P<0.05 were picked for identification using MALDI-TOF MS and TOF/TOF tandem MS/MS. Proteins identified with protein scores ≥200 as well as a protein score C.I. and total ion C.I. of 100% were entered into String 11.0 (string-db.org; accessed 07/22/2021) for pathway analyses.

**FIGURE 1:**
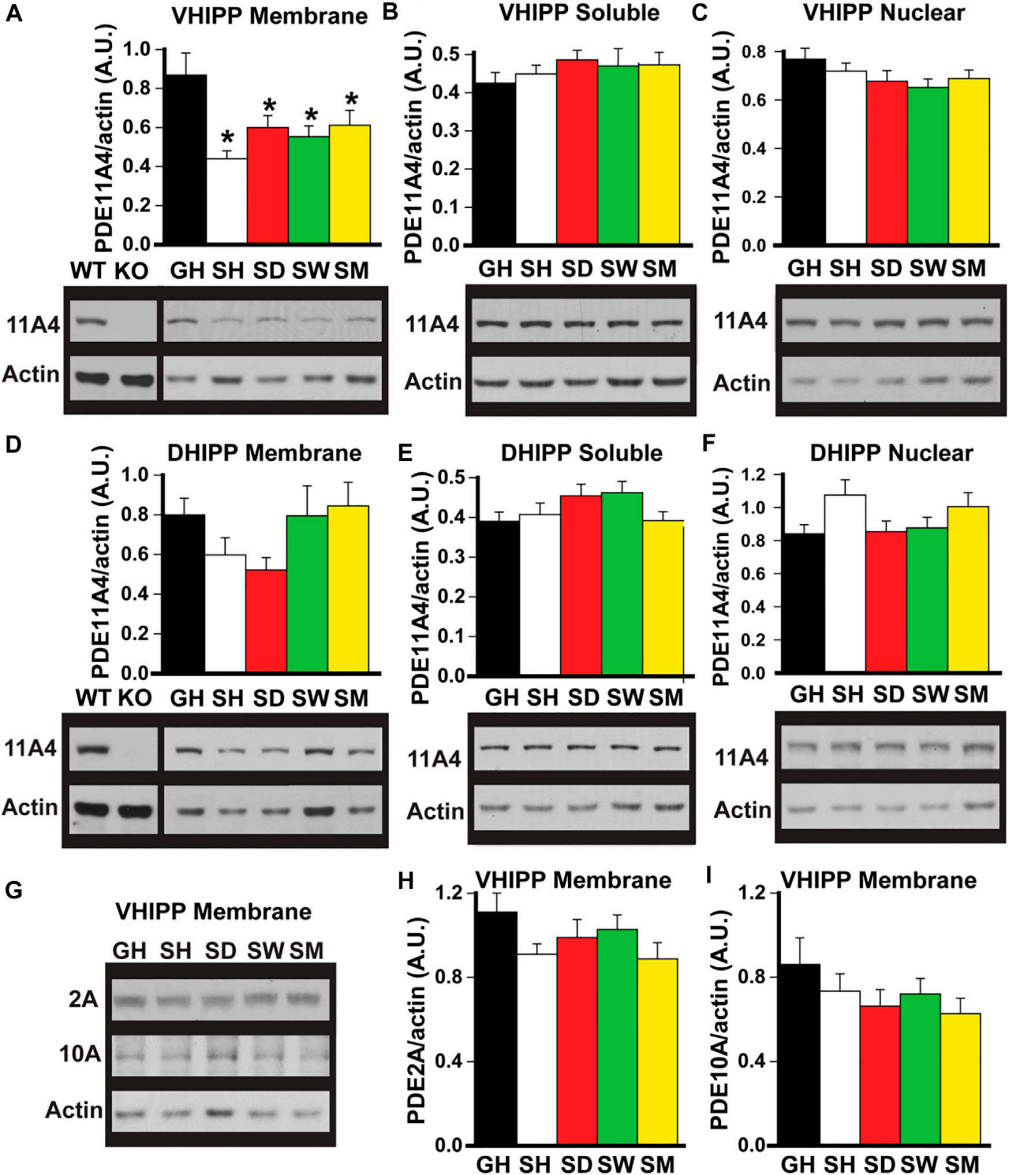
Social isolation selectively decreases PDE11A4 protein expression in ventral hippocampal membrane fractions of adult mice. Western blots were used to measure PDE protein expression in fractionated samples from hippocampi of adult group-housed mice (GH) and adult mice that were single-housed for 1 h (SH), 1 day (SD), 1 week (SW) or 1 month (SM). Samples from the hippocampi of a *Pde11a* wildtype (WT) and knockout (KO) mouse are included as a positive and negative control, respectively, for the PDE11A4 antibody. Cerebellum is run as a negative control on PDE2A blots (not shown—see Supplementary Data 1) and striatum is run as a positive control on PDE10A blots (not shown—see Supplementary Data 1). **(A)** Both acute and chronic isolation decreases PDE11A4 (∼97kD) protein expression in the VHIPP membrane fraction (n = 9–10 samples/time point/sex; effect of group: F (4,65) = 5.95, *p* < 0.001; *Post hoc:* each group vs GH, P = 0.007 to *p* < 0.001), **(B)** but not the VHIPP soluble fraction (effect of group: F (4,66) = 0.991, P = 0.42) **(C)** nor the VHIPP nuclear fraction (effect of group: F (4,66) = 1.50, P = 0.21). Relative to group housing, social isolation does not significantly change PDE11A4 expression in the dorsal hippocampal (DHIPP) membrane fraction. A 2-way RM ANOVA for all groups fails equal variance; however, separate analyses for GH vs SH, SD, and SM as well as GH vs SW each pass normality and equal variance (GH vs SH, SD, and SM effect of group: F (3,51) = 4.76, FDR-P = 0.011 with *Post hoc* for each group vs GH, P = 0.057-0.868; GH vs SW effect of group: F (1,34) = 0.36, FDR-P = 0.552), soluble fraction (effect of group: F (4,64) = 1.74, P = 0.152), or nuclear fraction (effect of group: F (4,69) = 2.85, P = 0.03 with *Post hoc* for each group vs GH, P = 0.057-0.866). **(G)** To determine whether the isolation-induced decreases in VHIPP membrane PDE11A4 are specific to this PDE family, protein expression of both PDE2A (∼98 kDA) and PDE10A (∼80 kDa) were measured in VHIPP membrane samples. Neither **(H)** PDE2A (effect of group: F (4,65) = 1.64, P = 0.18) nor **(I)** PDE10A (effect of group: F (4,67) = 1.54, P = 0.20) differed in expression among the groups. Post hoc *vs GH, P = 0.007 to <0.001. Data are plotted as means ± SEMs. Brightness and contrast of blot images adjusted for graphical clarity.

**FIGURE 2:**
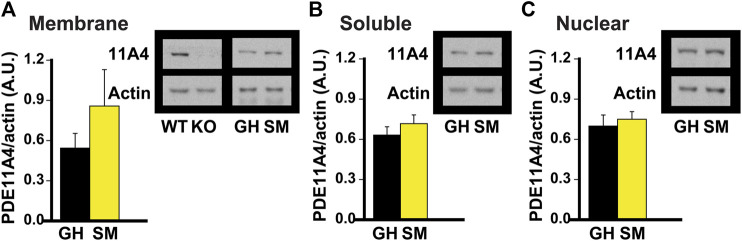
Chronic social isolation during adolescence does not change PDE11A4 protein expression in ventral hippocampal fractions. Adolescent group-housed mice (GH) demonstrate equivalent ventral hippocampal PDE11A4 protein expression (98kD) relative to isolated mice (single-housed for 1 month, SM; n = 6 samples/group/sex) in the **(A)** membrane fraction (effect of group: F (1,9) = 1.08, P = 324), **(B)** soluble fraction (effect of group: F (1,9) = 0.04, P = 0.838), and **(C)** nuclear fraction (effect of group: F (1,9) = 0.73m O = 0.411). Note that samples from the hippocampi of a Pde11a wildtype (WT) and knockout (KO) mouse were included as a positive and negative control, respectively, for the PDE11A4 antibody. Data are plotted as means ± SEMs. Brightness and contrast of blot images adjusted for graphical clarity.

**FIGURE 3:**
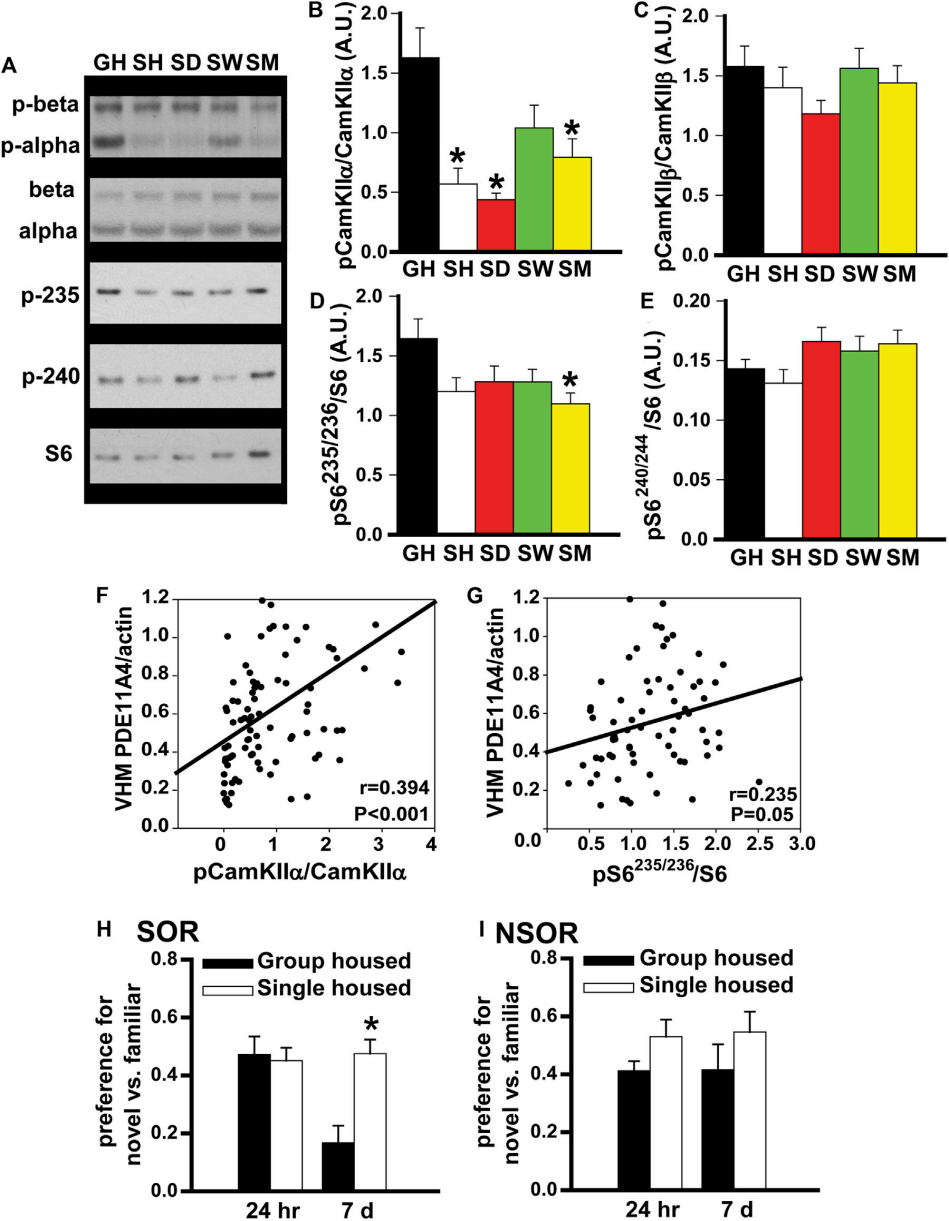
In adult mice, social isolation triggers changes in biochemical and behavioral endpoints that are consistent with reduced PDE11A4 activity. Previously, we reported PDE11A4 expression correlates with phosphorylation of CamKIIα but not CamKIIβ and that deletion of PDE11A4 reduces phosphorylation of S6-235/236 but not S6-240/244 (Kelly et al., 2014; Hegde et al., 2016a; Pilarzyk et al., 2019). Thus, we determined if social deletion of PDE11A4 reduces phosphorylation of S6-235/236 but not S6-240/244 [2; 3; 4]. Thus, we determined if social isolation would similarly affect these endpoints. **(A)** Consistent with the observed isolation-induced decreases in PDE11A4 described above, Western blots of adult group housed mice (GH) and adult mice single-housed for 1 h (SH), 1 day (SD), 1 week (SW), or 1 month (SM) show that social isolation significantly decreases phosphorylation of **(B)** CamKII⍺ (n = 9–10 samples/time point/sex). 2-way RM ANOVA for all groups fails normality; however, separate analyses for GH vs, SH, SD, and SM as well as GH vs SW each pass normality (GH vs SH, SD and SM effect of group: F (3,48) = 11.83, FDR-P <0.001 with *Post hoc*: GH vs SH, SD, SM, FDR-P<0.001; GH vs SW, effect of group: F (1,17) = 3.23, P = 0.09). **(C)** Phosphorylation of CamKIIβ does not change (n = 9–10 samples/time point/sex); 2-way RM ANOVA for all groups fails normality; however, separate analyses for GH vs, SH, SD, and SM as well as GH vs SW each pass normality (GH vs SH, SD and SM effect of group: F (3,48) = 1.60, FDR-P = 0.40; GH vs SW effect of group: F (1,16) = 0.009, FDR-P = 0.92). **(D)** Also consistent with reduced PDE11A4, 1 month of social isolation led to decreased phosphorylation of the ribosomal protein S6 at residues 235/236 (n = 9 samples/time point/sex; effect of group: F (4,83) = 2.86, P = 0.028; *Post Hoc*: GH vs SM, P = 0.018), **(E)** but not residues 240/244 (n = 9–10 samples/time point/sex; effect of group: F (4,67) = 2.16, P = 0.08). **(F)** Further, levels of membrane PDE11A4 in the ventral hippocampus (VHM) of the isolated mice correlates with phosphorylation of CamKII⍺ (r = 0.394, P < 0.001) and **(G)** S6-235/236 (r = 0.235, P = 0.05). **(H)** Social isolation also triggers behavioral changes that are associated with reduced PDE11A signaling (see phenotypes in *Pde11a* heterozygous mice, (Hegde et al., 2016b)). Relative to adult GH mice (n = 19), SM mice (n = 20) demonstrate normal social odor recognition (SOR) memory 24 h (24 h) after training (effect of group: F (2,41) = 1.53, P = 0.229) but significantly improved SOR memory 7 days (7 d) after training (effect of group: F (2,42) = 5.76, *p* = 0.006). **(I)** Importantly, we see that isolation has no effect on 24-h (effect of group: F (1,33) = 3.46, P = 0.072) or 7-d memory for non-social recognition (NSOR) (effect of group: F (1,33) = 1.41, P = 0.244). Post hoc *vs GH, *p* ≤ 0.018-0.0001. Data are plotted as means ± SEMs. Brightness and contrast of blot images adjusted for graphical clarity.

**FIGURE 4:**
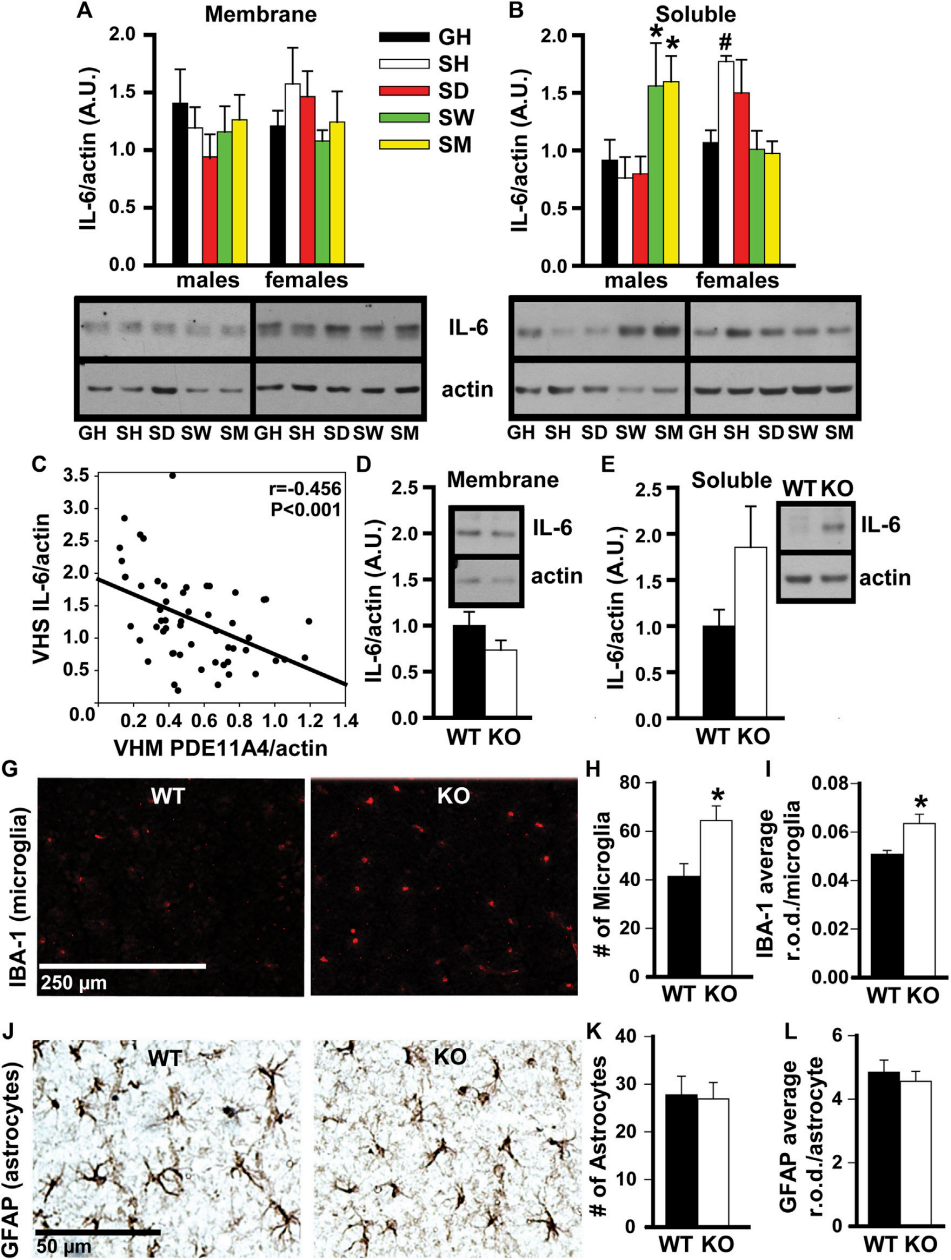
In adult mice, social isolation and decreased PDE11A4 signaling upregulate markers associated with neuroinflammation. Previous studies comparing expression of PDE11A and the cytokine interleukin-6 (IL-6) between mouse strains determined lower expression of PDE11A4 in ventral hippocampal membranes (VHM) correlated with higher expression of IL-6 in ventral hippocampal the soluble fractions (VHS) (Pathak et al., 2017). **(A)** Similarly, western blots of ventral hippocampal fractions from adult group housed mice (GH) versus adult mice single-housed for 1 h (SH), 1 day (SD), 1 week (SW), or 1 month (SM) show that social isolation does not significantly affect IL-6 expression in the membrane compartment of the VHIPP (n = 7–8/group/sex; effect of group x sex: F (4,55) = 1.58, P = 0.192), **(B)** but does increase IL-6 expression in the soluble fraction, albeit at different time points for each sex (n = 7–8/group/sex; effect of group x sex: F (4,51) = 6.52, *p* < 0.001; *Post hoc*: GH-M vs SM-M and SW-M, P = 0.046-0.025 and GH-F vs SH-F, P = 0.062; however, paired *t*-test GH-F vs SH-F: t (6) = 4.404, FDR-P = 0.02). Consistent with our previous work (Pathak et al., 2017), **(C)** expression of VHM PDE11A4 in isolated mice correlated negatively with VHS IL-6 (r = -0.456, *p* < 0.001). To determine if a loss of PDE11A4 signaling is sufficient to increase soluble IL-6, we compared IL-6 expression in VHIPP fractions from *Pde11a* WT vs KO mice (n = 8-9F, 2M/genotype). **(D)** Genetic deletion of PDE11A did not alter IL-6 expression in the membrane compartment (n = 10/genotype, t (8) = 1.45, *p* = 0.186) but **(E)** did increase IL-6 expression in the soluble fraction with an effect size comparable to that observed in the isolated mice (n = 10/genotype, t (8) = 2.033, *p* = 0.076). Next, we determined a potential source of this increased IL-6. **(G)** Immunofluorescence for a marker of microglia shows that, **(H)** relative to *Pde11a* WT mice, *Pde11a* KO mice exhibit more microglia in the VHIPP (n = 5/genotype, t (8) = -2.88, *p* = 0.02) **(I)** and higher levels of IBA-1 expression per microglia (n = 5/genotype, t (8) = -3.03, *p* = 0.02). **(J)** In contrast, we see no change in the number of astrocytes within the VHIPP of *Pde11a* KO mice (n = 4M and 5F/genotype; effect of genotype: t (16) = 0.17, P = 0.867) **(K)** nor in the level of GFAP expressed by each astrocyte (t (16) = 0.59, P = 0.566). Post Hoc *vs GH, P = 0.046-0.025; #vs GH, FDR-P = 0.02. Data are plotted as means ± SEMs. Brightness and contrast of images adjusted for graphical clarity.

*Data Analyses.* Biochemical and behavioral data were analyzed using Sigmaplot 11.0. Western blot samples were counterbalanced across blots. Due to the large number of samples included in the isolation timelines, coupled with the limited throughput of the fractionation process, samples were processed in multiple batches across multiple days. Each batch included 1 sample/time point, and samples from a batch were paired statistically by using repeated measures analyses to account for technical variances among runs. Samples were then distributed across multiple blots with 2 samples/time point in the adult study and six samples/time point in the adolescent study. Optical densities from all blots were normalized using the grand average of the mean optical density to account for any technical differences between blots (e.g., differences in transfer efficiencies, film exposures, etc). Data were analyzed by analysis of variance (ANOVA), Student t-test, or Pearson Product Moment Correlation. Data were analyzed for effect of sex (except in the 2DGE study where there were too few samples), isolation condition, and/or genotype. When there was no effect of sex, data were graphed collapsed across sexes. When analyses failed equal variance (Levene’s test) or normality (Shapiro-Wilk test), groups were broken apart into separate statistical tests that did pass normality and equal variance and a false discovery rate (FDR) was applied to *p*-values to reduce the impact of multiple tests. *Post hoc* analyses were performed on significant ANOVAs using the Student–Newman–Keuls Method. Any outlier greater than two standard deviations from the mean was removed (outliers/total data points: Figure 1A, 5/98; Figure 1B, 5/98; Figure 1C 6/98; Figure 1D, 2/98; Figure 1E, 7/98; Figure 1F, 3/98; Figure 1H, 6/98; Figure 1I, 4/98; Figure 2A 2/24; Figure 2B 1/24; Figure 2C 1/24; Figure 3B, 4/97; Figure 3C, 5/97; Figure 3D, 5/97; Figure 3E, 3/97; Figure 3H, 3/39; Figure 3I, 4/39; Figure 4B, 5/79; Figure 4D, 1/20; Figure 4E, 2/22). Significance was determined as *p* ≤ 0.05. Data are graphed as means ± SEM. All source data can be found in Supplementary Data 2.

## Results

*Social isolation selectively decreases PDE11A4 protein expression in ventral hippocampal membrane fractions of adult mice.* Previously, we showed that PDE11A4 protein was decreased in the membrane compartment of the VHIPP in adult mice following 1 month of social isolation (Hegde et al., 2016b). To determine whether this isolation-induced decrease in PDE11A4 protein required chronic social isolation or if it occurred acutely and was sustained long term, we devised an isolation timeline consisting of group housed (GH) and isolated adult mice that were single-housed for 1 h (SH), 1 day (SD), 1 week (SW), or 1 month (SM). Compared to group-housed controls, both acute and chronic social isolation in adult C57BL6/J was sufficient to decrease PDE11A4 protein expression in the membrane compartment of the VHIPP (Figure 1A). This loss of VHIPP membrane PDE11A4 was specific to this compartment as there was no change in PDE11A4 expression in the VHIPP soluble fraction, VHIPP nuclear fraction (Figures 1B,C) nor any compartments of the dorsal hippocampus (DHIPP) (Figures 1E–G). To determine whether these isolation-induced decreases observed in the VHIPP membrane were selective for PDE11A, we measured protein expression of both PDE2A and PDE10A as they are the closest related PDEs with appreciable expression in the VHIPP (Kelly et al., 2014; Kelly, 2014). Social isolation had no effect on membrane expression of PDE2A or PDE10A in VHIPP, suggesting that the isolation-induced decrease of PDE11A4 is selective (Figures 1H,I).

*Chronic social isolation during adolescence does not decrease PDE11A4 protein expression in ventral hippocampus.* Literature suggests that the adolescent brain is generally more sensitive to the effects of social isolation than is the adult brain in rodents (Lukkes et al., 2009; Burke et al., 2017; Arakawa, 2018; Walker et al., 2019). That said, hippocampal PDE11A4 expression is extremely low during early postnatal development (Hegde et al., 2016a) and the effect of PDE11A deletion on social behaviors during adolescence differs from that during adulthood (Hegde et al., 2016b). Therefore, we next determined whether chronic social isolation during adolescence would similarly affect PDE11A expression as does chronic social isolation during adulthood. We found that 1 month of social isolation during adolescence (starting on postnatal day 28) had no effect on PDE11A4 expression in any subcellular compartment of the VHIPP (Figure 2). This suggests that the regulation and function of PDE11A may differ in the adolescent versus adult brain.

*In adult mice, social isolation triggers changes in biochemical endpoints that are consistent with reduced PDE11A4 expression.* Previously, we showed that PDE11A4 expression within the hippocampus correlates positively with phosphorylation of CamKIIα but not CamKIIβ (Kelly et al., 2014), and that PDE11A deletion decreases phosphorylation of the ribosomal protein S6 at residues 235/236 but not residues 240/244 (Hegde et al., 2016a; Pilarzyk et al., 2019). Thus, we examined the expression of these biochemical endpoints in our group-housed and isolated mice. Consistent with our observation of reduced PDE11A4 expression, we saw a robust decrease in phosphorylation of CamKIIα in the SH, SD, and SM isolated groups, but no change in phosphorylation of CamKIIβ (Figures 3B,C). We also found that chronic social isolation reduced phosphorylation of S6-235/256 in the SM group, but that neither acute nor chronic social isolation had a significant effect on phosphorylation of S6-240/244 (Figures 3D,E). Not only did we see these group effects, we also saw that PDE11A4 expression levels in the membrane compartment of VHIPP correlated with levels of phosphorylation of CamKIIα and pS6-235 across all isolated mice (Figures 3F,G). Taken together, these results suggest that isolation-induced decreases in PDE11A4 expression are sufficient to trigger changes in relevant signal transduction cascades.

*In adult mice, social isolation changes social odor recognition memory in a manner that is consistent with reduced PDE11A4 expression.* Previously we showed that *Pde11a* heterozygous (HT) mice exhibit normal recent long-term memory (24 h) and enhanced remote long-term memory (7 days) for social odor recognition (SOR) (Pilarzyk et al., 2019). In contrast, *Pde11a* KO mice show impaired recent long-term memory and enhanced remote long-term SOR memory. Neither HT nor KO mice exhibit any changes in memory for non-social odor recognition (NSOR) at either time point (Pilarzyk et al., 2019). Since social isolation results in only a partial loss of PDE11A expression, we sought to determine if social isolation would yield a pattern of behavioral phenotypes similar to that observed in *Pde11a* HT mice. Indeed, compared to GH mice, we see here that mice single housed for 1 month exhibit intact SOR memory 24 h after training and significantly improved SOR memory 7 days after training (Figure 3H) with no effects on recent or remote NSOR memory (Figure 3I). These data suggest that social isolation, like PDE11A deletion, preferentially affects social versus non-social memories.

*In adult mice, social isolation and decreased PDE11A4 signaling upregulate markers associated with neuroinflammation.* Social isolation is known to increase markers of neuroinflammation in humans, particularly the cytokine interleukin 6 (IL-6) (Karelina et al., 2009; Häfner et al., 2011; Slavich and Cole, 2013). Previous studies in mice that investigated the relationship between PDE11A expression and IL-6 expression found that lower expression of PDE11A4 in the membrane compartment of the HIPP correlated with higher expression of IL-6 in the soluble fraction (Pathak et al., 2017). We saw here that social isolation increased IL-6 expression in the soluble fraction but not the membrane compartment of the VHIPP (Figure 4A). Surprisingly, the time course of isolation-induced increases in IL-6 expression was sex-dependent. Male mice show increased IL-6 with prolonged isolation, as seen in the SW and SM isolated groups. In contrast, female mice show increased IL-6 acutely, as seen in the SH and SD isolated groups (Figure 4B). These isolation-induced increases in the soluble IL-6 correlated with the reductions in membrane VHIPP PDE11A4 (Figure 4C), consistent with our previous study described above. The isolation-induced loss of membrane PDE11A4 is likely driving the upregulation in the soluble IL-6 (as opposed to vice versa), because pathway analyses of proteomic changes in VHIPP of *Pde11a* KO vs WT mice identified “positive regulation of IL-6 secretion” as the second strongest GO Biological Process (Table 1). Further, genetic deletion of PDE11A increased IL-6 expression in the cytosol—but not membrane-with an effect size comparable to that observed in the isolated mice (Figures 4D,E).

**TABLE 1:** Pathway analyses of proteins that differed in expression in the VHIPP of *Pde11a* KO vs WT mice.

#Term ID	Term description	Strength	FDR-P
Biological Process			
GO:0006108	malate metabolic process	2.63	0.006
GO:2000778	positive regulation of interleukin-6 secretion	2.1	0.022
GO:0006099	tricarboxylic acid cycle	2.1	0.022
GO:0006101	citrate metabolic process	2.04	0.022
GO:0061077	chaperone-mediated protein folding	1.75	0.022
GO:0051702	interaction with symbiont	1.69	0.022
GO:0050715	positive regulation of cytokine secretion	1.59	0.017
GO:0046496	nicotinamide nucleotide metabolic process	1.53	0.034
Cellular Component			
GO:0044291	cell-cell contact zone	1.86	0.001
GO:0005881	cytoplasmic microtubule	1.8	0.008
GO:0014704	intercalated disc	1.78	0.008
GO:0043209	myelin sheath	1.75	0.000
GO:0070062	extracellular exosome	1.63	0.011
GO:0055037	recycling endosome	1.34	0.017
GO:0036464	cytoplasmic ribonucleoprotein granule	1.27	0.021
GO:0045121	membrane raft	1.26	0.002
KEGG Pathways			
mmu00020	Citrate cycle (TCA cycle)	2.03	0.002
mmu00620	Pyruvate metabolism	1.95	0.002
mmu01200	Carbon metabolism	1.63	0.002
mmu04540	Gap junction	1.6	0.009
mmu04145	Phagosome	1.49	0.002
mmu04210	Apoptosis	1.4	0.018
mmu05010	Alzheimer’s disease	1.31	0.022
mmu04530	Tight junction	1.31	0.022

Protein expression changes measured by 2DGE, followed by MALDI-TOF MS, and TOF/TOF, tandem MS/MS. Only the top eight pathways (defined by strength) for each category are shown. See supplement for full list of significant pathways.

To determine a potential source of this increase in IL-6, microglia and astrocytes were compared between *Pde11a* WT vs KO mice. Glial cells produce the majority of IL-6 in the brain (Arisi, 2014). As such, we determined if reducing PDE11A expression is sufficient to alter microglia or astrocytes in the VHIPP. To do so, we labeled the VHIPP of adult *Pde11a* WT and KO mice with antibodies recognizing either ionized calcium-binding adaptor protein-1 expression (IBA-1), a microglial marker (Hoogland et al., 2018), or glial fibrillary acidic protein (GFAP), an astrocyte marker. We then quantified the number of labeled microglia and astrocytes along with the intensity of IBA-1 staining per microglia and GFAP staining per astrocyte. We found a significant increase in the number of microglia present within the VHIPP of *Pde11a* KO relative to WT mice and an increase in IBA-1 expression (Figures 4H,I). In contrast, we saw no change in the number of astrocytes within the VHIPP of *Pde11a* KO nor the levels of GFAP expressed by astrocytes (Figure 4J). Parsimoniously, these data suggest that the loss of PDE11A4 signaling may increase IL-6 expression by activating microglia.

## Discussion

Our studies here suggest PDE11A4 may be a key molecular mechanism by which social isolation increases neuroinflammation and alters social behaviors. We showed that both acute and chronic social isolation in adult mice decreases PDE11A4 expression selectively in the membrane compartment of the VHIPP, suggesting the ability of social isolation to regulate PDE11A4 in the adult hippocampus occurs acutely and is sustained long-term (Figures 1A–C). Our findings add to a growing literature identifying biochemical, structural, and behavioral consequences of acute and/or chronic social isolation (Hegde et al., 2016b; Shahar-Gold et al., 2013; Monteiro et al., 2014; Pereda-Pérez et al., 2013; Zovetti et al., 2021; Liu et al., 2018). In contrast, adolescent mice show no effect of chronic social isolation on PDE11A4 expression in the hippocampus (Figure 2). The lack of effect in adolescent mice may reflect the fact that PDE11A4 is expressed at significantly lower levels during early development than adulthood (Hegde et al., 2016a), and is consistent with the fact that deletion of PDE11A during adolescence does not impact social behaviors to the same extent as does PDE11A deletion during adulthood (Hegde et al., 2016b). This suggests the role of PDE11A in regulating social behaviors and in mediating the effect of social experience on the brain evolves across the lifespan. Importantly, social isolation in adults triggers changes in signal transduction (i.e., decreased phosphorylation of CamKIIα and S6-236/236; Figures 3A–G) and social recognition memory that are consistent with reduced PDE11A expression (Figures 3H,I; (Kelly et al., 2014; Hegde et al., 2016a; Pilarzyk et al., 2019)). Interestingly, isolation-induced decreases in membrane PDE11A4 appear to drive specific increases in soluble interleukin-6 (IL-6; Figures 4A,B), a neuroinflammatory marker associated with social isolation and feelings of loneliness in humans (Karelina et al., 2009; Häfner et al., 2011; Slavich and Cole, 2013). These data, along with our previous work (Hegde et al., 2016b), suggest that social isolation causes alterations in subsequent social behaviors by decreasing membrane-associated PDE11A4 signaling and, thus, increasing markers of neuroinflammation in the ventral hippocampus.

### Acute and Chronic Social Isolation Selectively Decrease PDE11A4 in the Membrane Compartment of the Ventral Hippocampus

Although we found social isolation decreased PDE11A4 expression in the membrane compartment of VHIPP, we found no such effect on PDE2A or PDE10A expression (Figure 1G–I). Inhibiting PDE2A improves social recognition memory (Boess et al., 2004), lessens social withdrawal behavior in a rodent model of schizophrenia (Nakashima et al., 2018), and rescues social deficits in *Fmr1* KO animals (Maurin et al., 2019). Additionally, inhibiting PDE10A restores social behaviors in mice with a partial loss of miR-137, a psychiatric risk gene that impairs social behaviors (Cheng et al., 2018), and increases sociability and social recognition in mice (Grauer et al., 2009b). However, deleting PDE11A alters the consolidation of social memories as well as the dynamics of social interactions (Kelly et al., 2010; Hegde et al., 2016a; Hegde et al., 2016b; Pilarzyk et al., 2019; Smith et al., 2021). The fact that the effects of social isolation are specific to PDE11A is consistent with the fact that no two PDE isoforms share the exact same regional distribution or subcellular compartmentalization in the brain (Bender and Beavo, 2006; 2018 and Brandon, 2009; Lakics et al., 2010; Francis et al., 2011; Xu et al., 2011; Kelly et al., 2014; Kelly, 2018a). This suggests that while PDE2A, PDE10A, and PDE11A each regulate social behaviors, the ability of social experiences to feedback and regulate phosphodiesterase function is specific to the PDE11A family. Taken together, these data may suggest that PDE11A4 is better positioned than PDE2A or PDE10A as a therapeutic target for neuropsychiatric disorders associated with social withdrawal and/or loneliness.

### PDE11A Regulates Cyclic Nucleotide Signaling in the VHIPP and Modulates Social Behaviors

The ventral hippocampal formation plays an important role in affective, motivational, and social behaviors (Tseng et al., 2009; Fanselow and Dong, 2010). Of particular note, lesion studies have shown that this region is required for proper formation of social memories (Kogan et al., 2000). Further, optogenetic studies have shown that ventral CA1 projections to the nucleus accumbens are required for social memory (Okuyama et al., 2016). Interestingly, the effect of a VHIPP lesion on social behaviors is mimicked by knock down of cAMP response element binding protein (CREB) (Brightwell et al., 2005). Given that PDE11A regulates both cAMP and cGMP, is tightly restricted to the hippocampal formation, and is enriched in VHIPP neurons that project to the nucleus accumbens, it may not be surprising that PDE11A plays a role in modulating social behaviors. Indeed, alterations in PDE11A signaling are sufficient to change biochemical pathways important for social behaviors. *Pde11a* KO mice show higher levels of nuclear CREB in the VHIPP relative to WT mice (Smith et al., 2021). *Pde11a* KO mice also shown an increased sensitivity to MK-801, as well as biochemical changes related to the function of AMPA and NMDA receptors, which suggests alterations in glutamatergic signaling (Kelly et al., 2010; Pilarzyk et al., 2019). Lastly, deleting PDE11A triggers changes in the oxytocin signaling pathway, a pathway crucial in regulating social behaviors (Hegde et al., 2016b). Thus, the isolation-induced decreases in PDE11A4 signaling that are described herein are consistent with what is known of cyclic nucleotide signaling and VHIPP regulation of social behaviors.

### Isolation-Induced Decreases in PDE11A May Affect Behavior via Neuroinflammatory Signals

Isolation-induced decreases in membrane PDE11A4 corresponded not only with changes in PDE11A-relevant signal transduction (Figures 3B–G), but also with an increase in soluble IL-6 (Figure 4B). This correlation between lower membrane PDE11A4 and higher soluble IL-6 is consistent with our previous work correlating PDE11A4 and IL-6 protein expression across mouse strains (Pathak et al., 2017), as well as our proteomic experiment described herein where pathway analyses identified both soluble and membrane cellular compartments as well as the biological process of “positive regulation of interleukin-6 secretion” (Table 1). Upon biochemical fractionation of the hippocampus, IL-6 can be found in the soluble, membrane, and nuclear fractions (Porcher et al., 2021). IL-6 promotes proinflammatory signaling via its soluble receptor and anti-inflammatory signaling via its transmembrane receptor (Lin and Levison, 2009; Scheller et al., 2011). These proinflammatory versus anti-inflammatory IL-6 signaling cascades are often referred to as the “*trans*-signaling” versus “classic” cascade, respectively (Lin and Levison, 2009; Scheller et al., 2011). IL-6 in the nuclear fraction may indicate a non-canonical cytokine signaling pathway in which cytokine-bound receptors are transported to the nucleus (Olsnes et al., 2003)**.** The fact that isolation increased soluble IL-6 expression parsimoniously argues for activation of the pro-inflammatory IL-6 pathway within the VHIPP. Given that social isolation is thought to cause aging-like pathology (Hawkley and Cacioppo, 2007), it is interesting to note that increases in VHIPP IL-6 that are associated with aging are also more pronounced in the soluble versus membrane or nuclear fractions (Porcher et al., 2021). Others have similarly found increased soluble IL-6 expression in the HIPP and changes in rodent behavior following social isolation in male rats (Peric et al., 2017). In our hands, IL-6 increased in female VHIPPs only following acute isolation (i.e., 1 h); whereas, IL-6 increased in male VHIPPs only with prolonged isolation (i.e., 1 week and 1 month). Others have similarly observed sex-specific protein expression changes following social isolation (Wall et al., 2012), with females showing acute effects only (Snyder et al., 2015) and males less able to habituate to environmental changes (Weiss et al., 2001; Weiss and Feldon, 2001; Pietropaolo et al., 2008). It is interesting to speculate that this sex-specific pattern of isolation-induced regulation of IL-6 may help explain why it is possible to return isolated female mice on a C57BL/6J background—but not male mice—to a group housing environment without risk of fighting. Together, these data suggest that biologics that inhibit IL-6 *trans*-signaling [c.f., (Rothaug et al., 2016)] may hold promise in treating social deficits. Indeed, inhibiting IL-6 *trans*-signaling improves social behavior in a mouse model of autism (Wei et al., 2016).

It remains to be determined how lower levels of PDE11A4 could drive higher expression of IL-6, but results here suggest the mechanism may be related to an increase in microglial activity. In the brain, the majority of IL-6 is released into the extracellular space by microglia and astrocytes, with neurons able to synthesize and release much smaller pools (Arisi, 2014; Tsakiri et al., 2008). While PDE11A4 is only expressed in neurons (Hegde et al., 2016a), we find that *Pde11a* KO mice exhibit more microglia and higher expression of IBA-1 in VHIPP than do WT mice, with no change in the number of astrocytes or GFAP expression (Figure 4G–L). Interestingly, hippocampal changes in microglial markers, including IBA-1, have been associated with various types of psychosocial stress (Calcia et al., 2016) as well as neuropsychiatric disorders that alter social behaviors (Morgan et al., 2012; Réus et al., 2015). Therefore, our data parsimoniously argue that reductions in neuronal PDE11A signaling may ultimately activate microglia to increase production and/or release of IL-6. Indeed, microglia are highly responsive to their environments and, much like PDE11A4, signal via discrete microdomains (Umpierre et al., 2020).

Microglia respond to a number of neuronally-released molecules that either activate (i.e., “on signals”) or deactivate/inhibit microglia (i.e., “off signals”) (Biber et al., 2007; Marinelli et al., 2019). The strongest candidate on/off signals by which PDE11A4 in neurons could regulate microglial activation and subsequent IL-6 release are adenosine, glutamate, and CX3CL1. cAMP can be extruded to the extracellular space via multi-drug resistance proteins, where it is catabolized in a 2-step process to adenosine (Jackson et al., 2007). Thus, by increasing cAMP levels intracellularly, PDE11A deletion could increase extracellular adenosine. In so doing, deletion of PDE11A may trigger inflammation-associated microglial remodeling by increasing adenosine signaling at microglia A2A receptors (Orr et al., 2009). Alternatively, glutamate is known to activate microglia (Biber et al., 2007). Previously, we showed that deletion of PDE11A alters glutamatergic signaling in the hippocampus (Pilarzyk et al., 2019; Kelly et al., 2010). Dysregulation of glutamatergic signaling is thought to contribute to the pathophysiology of schizophrenia, a neuropsychiatric disorder associated with deficits in social behaviors (Parellada and Gassó, 2021). Social isolation has also been shown to decrease the release of glutamate in the hippocampus (Almeida-Santos et al., 2019). Therefore, isolation-induced decreases in PDE11A4 could be sufficient to trigger alterations in glutamatergic signaling that activate microglial release of cytokines. Lastly, CX3CL1 is a chemokine ligand expressed specifically by neurons that targets a receptor (CX3CR1) expressed solely on microglia (Wolf et al., 2013), thereby preventing cytokine release (Biber et al., 2007). As described above, pathway analysis of protein expression changes in the VHIPP of *Pde11a* WT vs KO mice did show enrichment for “regulation of cytokine secretion” (Table 1). Deletion of PDE11A phenocopies the loss of CX3CR1 in mice in terms of reducing social interactions with WT mice and altering learning and memory (Chamera et al., 2020). The fact that CX3CR1 loss reduces social interactions with WT mice is consistent with reports showing disrupted CX3CL1-CX3CR1 signaling is associated with schizophrenia and depression-two diseases characterized by social withdrawal/isolation (Chamera et al., 2020). That said, social isolation in C57BL6/J mice increased CX3CR1 expression in several brain regions, including the hippocampus (Zhou et al., 2020). Thus, it will be of interest to future studies to determine if PDE11A, a neuron-specific protein, regulates microglial activation and cytokine release via adenosine, glutamate, and/or CX3CL1.

Although the early stages of an inflammatory response can be neuroprotective, chronic activation can be detrimental (Wyss-Coray, 2016; von Bernhardi et al., 2010; Weber et al., 2015; Bodles and Barger, 2004). Thus, it remains to be determined if the increases in neuroinflammatory markers described herein represent a deleterious effect of lost PDE11A signaling or a protective compensatory mechanism. The increased microglia activation described above is consistent with the measured increase in IL-6, both of which are often characterized as impairing neuroinflammatory events. Increases in inflammatory cytokines, including IL-6, in humans and rodents correlate with impairments in cognitive function (Wei et al., 2012b; Sparkman et al., 2006; Godbout and Johnson, 2004; Balschun et al., 2004). Increases in other cytokines that promote anti-inflammatory pathways, however, could also be changed with isolation. The fact that anti-inflammatory cytokines were not measured here is a limitation of the current study. As discussed above, microglia can be activated or inhibited by neuronally-released cytokines (Biber et al., 2007; Marinelli et al., 2019), and we show here that the deletion of PDE11A appears to increase microglial activation and cytokine release in the VHIPP. That said, microglia activation does have a positive role to play in normal brain development and functioning, particularly with regard to maintaining neuronal networks (Chung et al., 2015) and synaptic pruning (Favuzzi et al., 2021). Indeed, the role of PDE11A signaling itself in regulating social behaviors is not straight forward (Pilarzyk et al., 2019; Hegde et al., 2016a; Kelly et al., 2010; Hegde et al., 2016b; Smith et al., 2021). For example, *Pde11a* KO mice demonstrate a transient amnesia for social memories, with normal short-term memory, no recent long-term memory, and spontaneously improved remote LTM relative to WT littermates (Pilarzyk et al., 2019). *Pde11a* HT mice show a shifted transient amnesia phenotype with disruption of short-term memory, normal recent short-term memory, and enhanced remote long-term memory (Pilarzyk et al., 2019). Consistent with these phenotypes observed in *Pde11a* mutant mice, we show here that isolated mice show stronger remote long-term social odor recognition memory (Figure 3H). In terms of social interactions, we also see a complex phenotype associated with PDE11A deletion. For example, *Pde11a* WT prefer to spend time with *Pde11a* WT over KO mice, but *Pde11a* KO prefer to interact with *Pde11a* KO versus WT mice (Hegde et al., 2016b; Smith et al., 2021). As described above, disrupting membrane-associated PDE11A4 in CA1 is sufficient to alter this social preference (Smith et al., 2021). Together, these data suggest isolation-induced decreases in membrane PDE11A4 may not cause social deficits per se, but rather differences in social behaviors.

## Data Availability

The original contributions presented in the study are included in the article/Supplementary Material, further inquiries can be directed to the corresponding authors.
