# Supplementary material for: The Role of PDE11A4 in Social Isolation-Induced Changes in Intracellular Signaling and Neuroinflammation
Source: Front Pharmacol. 2021 Nov 23;12:749628. doi: 10.3389/fphar.2021.749628 (PMC8650591; doi:10.3389/fphar.2021.749628)
Supplement: Supplementary file 1 [file DataSheet2.PDF]

Fig1A VHM PDE11/Actin

squads	id	group	sex	e11/actin	n no outs
6	GH11	GH	male	0.16515	0.16515
7	GH13	GH	male	0.18335	0.18335
3	GH5	GH	male	0.30972	0.30972
11	GH6	GH	female	0.36549	0.36549
19	GH19	GH	male	0.51938	0.51938
10	GH4	GH	female	0.56732	0.56732
4	GH7	GH	male	0.64856	0.64856
17	GH17	GH	male	0.75934	0.75934
2	GH3	GH	male	0.76222	0.76222
15	GH14	GH	female	0.76632	0.76632
16	GH16	GH	female	0.92487	0.92487
12	GH8	GH	female	1.00585	1.00585
18	GH18	GH	female	1.05468	1.05468
14	GH12	GH	female	1.05947	1.05947
20	GH20	GH	female	1.06635	1.06635
9	GH2	GH	female	1.20031	1.20031
1	GH1	GH	male	1.38436	1.38436
8	GH15	GH	male	1.83378	1.83378
5	GH9	GH	male	1.93373	1.93373
13	GH10	GH	female	2.06597	
13	SH10	SH	female	0.15392	0.15392
20	SH20	SH	female	0.28104	0.28104
14	SH12	SH	female	0.33714	0.33714
17	SH17	SH	male	0.34713	0.34713
5	SH9	SH	male	0.36353	0.36353
15	SH14	SH	female	0.37431	0.37431
6	SH11	SH	male	0.38085	0.38085
1	SH1	SH	male	0.38358	0.38358
11	SH6	SH	female	0.38495	0.38495
2	SH3	SH	male	0.42611	0.42611
7	SH13	SH	male	0.43128	0.43128
3	SH5	SH	male	0.45201	0.45201
10	SH4	SH	female	0.49926	0.49926
18	SH18	SH	female	0.55483	0.55483
9	SH2	SH	female	0.56613	0.56613
16	SH16	SH	female	0.6311	0.6311
19	SH19	SH	male	0.91023	0.91023
12	SH8	SH	female	1.0494	
4	SH7	SH	male	1.23693	
12	SD8	SD	female	0.23788	0.23788
14	SD12	SD	female	0.24346	0.24346
10	SD4	SD	female	0.25681	0.25681
11	SD6	SD	female	0.28211	0.28211
13	SD10	SD	female	0.36907	0.36907
2	SD3	SD	male	0.46589	0.46589
20	SD20	SD	female	0.48351	0.48351
18	SD18	SD	female	0.5133	0.5133
9	SD2	SD	female	0.58459	0.58459
16	SD16	SD	female	0.61517	0.61517
6	SD11	SD	male	0.62356	0.62356
7	SD13	SD	male	0.67815	0.67815
4	SD7	SD	male	0.71086	0.71086
1	SD1	SD	male	0.737	0.737
8	SD15	SD	male	0.74047	0.74047
17	SD17	SD	male	0.76551	0.76551
15	SD14	SD	female	0.85379	0.85379
19	SD19	SD	male	1.04657	1.04657
5	SD9	SD	male	1.19375	1.19375
3	SD5	SD	male	1.36771	
3	SW5	SW	male	0.14858	0.14858
7	SW13	SW	male	0.18402	0.18402
10	SW4	SW	female	0.33028	0.33028
6	SW11	SW	male	0.35673	0.35673
18	SW18	SW	female	0.38853	0.38853
15	SW14	SW	female	0.42092	0.42092
1	SW1	SW	male	0.42133	0.42133
2	SW3	SW	male	0.46307	0.46307
5	SW9	SW	male	0.46957	0.46957
12	SW8	SW	female	0.51224	0.51224
14	SW12	SW	female	0.5142	0.5142
20	SW20	SW	female	0.59987	0.59987
19	SW19	SW	male	0.61142	0.61142
4	SW7	SW	male	0.76057	0.76057
16	SW16	SW	female	0.77695	0.77695
17	SW17	SW	male	0.81522	0.81522
13	SW10	SW	female	0.83695	0.83695
8	SW15	SW	male	0.8916	0.8916
9	SW2	SW	female	1.00626	1.00626
11	SW6	SW	female	1.59587	
3	SM5	SM	male	0.12238	0.12238
7	SM13	SM	male	0.1336	0.1336
6	SM11	SM	male	0.15255	0.15255
10	SM4	SM	female	0.23564	0.23564
17	SM17	SM	male	0.34392	0.34392
14	SM12	SM	female	0.35059	0.35059
2	SM3	SM	male	0.48146	0.48146
12	SM8	SM	female	0.52729	0.52729
19	SM19	SM	male	0.57724	0.57724
15	SM14	SM	female	0.66385	0.66385
16	SM16	SM	female	0.66866	0.66866
11	SM6	SM	female	0.73487	0.73487
13	SM10	SM	female	0.76518	0.76518
18	SM18	SM	female	0.76793	0.76793
4	SM7	SM	male	0.93862	0.93862
8	SM15	SM	male	0.95011	0.95011
20	SM20	SM	female	0.98591	0.98591
5	SM9	SM	male	1.05653	1.05653
1	SM1	SM	male	1.17093	1.17093
9	SM2	SM	female	1.44936	

Fig1B **VH C PDE11/Actin**

squads	id	group	sex	e11/actin	n no outs
8	SW15	SW	male	0.07385	0.07385
4	SW7	SW	male	0.28049	0.28049
20	SW20	SW	female	0.29222	0.29222
2	SW3	SW	male	0.35927	0.35927
19	SW19	SW	male	0.37067	0.37067
11	SW6	SW	female	0.37563	0.37563
12	SW8	SW	female	0.41037	0.41037
9	SW2	SW	female	0.42009	0.42009
16	SW16	SW	female	0.43801	0.43801
5	SW9	SW	male	0.43955	0.43955
13	SW10	SW	female	0.44406	0.44406
14	SW12	SW	female	0.44626	0.44626
15	SW14	SW	female	0.54656	0.54656
6	SW11	SW	male	0.55917	0.55917
10	SW4	SW	female	0.60541	0.60541
17	SW17	SW	male	0.78426	0.78426
7	SW13	SW	male	0.79344	0.79344
18	SW18	SW	female	0.82843	0.82843
3	SW5	SW	male	1.02686	
1	SW1	SW	male	1.04655	
8	SM15	SM	male	0.17424	0.17424
2	SM3	SM	male	0.19464	0.19464
4	SM7	SM	male	0.35587	0.35587
20	SM20	SM	female	0.37372	0.37372
15	SM14	SM	female	0.37856	0.37856
6	SM11	SM	male	0.3814	0.3814
12	SM8	SM	female	0.41143	0.41143
19	SM19	SM	male	0.47761	0.47761
11	SM6	SM	female	0.47901	0.47901
9	SM2	SM	female	0.48347	0.48347
16	SM16	SM	female	0.52341	0.52341
5	SM9	SM	male	0.53111	0.53111
1	SM1	SM	male	0.53128	0.53128
10	SM4	SM	female	0.57254	0.57254
17	SM17	SM	male	0.57676	0.57676
13	SM10	SM	female	0.58019	0.58019
14	SM12	SM	female	0.59199	0.59199
18	SM18	SM	female	0.61632	0.61632
3	SM5	SM	male	0.74948	0.74948
7	SM13	SM	male	0.91896	

Fig 1C **VHN PDE11/Actin**

squads	id	group	sex	e11/actin n	no outs
7	GH13	GH	male	0.44274	0.44274
17	GH17	GH	male	0.48829	0.48829
10	GH4	GH	female	0.55991	0.55991
1	GH1	GH	male	0.60291	0.60291
11	GH6	GH	female	0.63227	0.63227
13	GH10	GH	female	0.65434	0.65434
12	GH8	GH	female	0.65738	0.65738
5	GH9	GH	male	0.70859	0.70859
16	GH16	GH	female	0.72215	0.72215
3	GH5	GH	male	0.74074	0.74074
4	GH7	GH	male	0.76725	0.76725
20	GH20	GH	female	0.81996	0.81996
9	GH2	GH	female	0.83862	0.83862
8	GH15	GH	male	0.89137	0.89137
18	GH18	GH	female	0.89205	0.89205
15	GH14	GH	female	0.95034	0.95034
2	GH3	GH	male	0.999	0.999
14	GH12	GH	female	1.07924	1.07924
19	GH19	GH	male	1.16955	1.16955
6	GH11	GH	male	1.23376	
19	SH19	SH	male	0.44134	0.44134
20	SH20	SH	female	0.45937	0.45937
1	SH1	SH	male	0.51669	0.51669
15	SH14	SH	female	0.62917	0.62917
5	SH9	SH	male	0.63223	0.63223
9	SH2	SH	female	0.6675	0.6675
14	SH12	SH	female	0.68406	0.68406
10	SH4	SH	female	0.68742	0.68742
17	SH17	SH	male	0.74824	0.74824
11	SH6	SH	female	0.7574	0.7574
16	SH16	SH	female	0.76452	0.76452
13	SH10	SH	female	0.77036	0.77036
12	SH8	SH	female	0.80926	0.80926
7	SH13	SH	male	0.84134	0.84134
6	SH11	SH	male	0.85245	0.85245
4	SH7	SH	male	0.86083	0.86083
2	SH3	SH	male	0.88859	0.88859
18	SH18	SH	female	0.93678	0.93678
3	SH5	SH	male	1.07476	
10	SD4	SD	female	0.33277	0.33277
14	SD12	SD	female	0.43488	0.43488
4	SD7	SD	male	0.47262	0.47262
20	SD20	SD	female	0.47796	0.47796
11	SD6	SD	female	0.5212	0.5212
5	SD9	SD	male	0.55086	0.55086
1	SD1	SD	male	0.58995	0.58995
19	SD19	SD	male	0.59748	0.59748
3	SD5	SD	male	0.61669	0.61669
6	SD11	SD	male	0.6822	0.6822
15	SD14	SD	female	0.73673	0.73673
16	SD16	SD	female	0.75905	0.75905
7	SD13	SD	male	0.76008	0.76008
2	SD3	SD	male	0.76372	0.76372
13	SD10	SD	female	0.79259	0.79259
17	SD17	SD	male	0.82595	0.82595
8	SD15	SD	male	0.93426	0.93426
18	SD18	SD	female	0.98395	0.98395
12	SD8	SD	female	1.02501	1.02501
9	SD2	SD	female	1.12161	
2	SW3	SW	male	0.43757	0.43757
4	SW7	SW	male	0.44849	0.44849
5	SW9	SW	male	0.49849	0.49849
9	SW2	SW	female	0.52393	0.52393
10	SW4	SW	female	0.53763	0.53763
16	SW16	SW	female	0.55844	0.55844
19	SW19	SW	male	0.59334	0.59334
6	SW11	SW	male	0.60273	0.60273
3	SW5	SW	male	0.62397	0.62397
8	SW15	SW	male	0.63795	0.63795
14	SW12	SW	female	0.65118	0.65118
18	SW18	SW	female	0.67218	0.67218
20	SW20	SW	female	0.67519	0.67519
11	SW6	SW	female	0.67917	0.67917
15	SW14	SW	female	0.72269	0.72269
7	SW13	SW	male	0.76694	0.76694
13	SW10	SW	female	0.77856	0.77856
17	SW17	SW	male	0.96955	0.96955
12	SW8	SW	female	1.00772	1.00772
1	SW1	SW	male	1.3163	
11	SM6	SM	female	0.43039	0.43039
18	SM18	SM	female	0.51322	0.51322
13	SM10	SM	female	0.54243	0.54243
1	SM1	SM	male	0.55674	0.55674
2	SM3	SM	male	0.5726	0.5726
3	SM5	SM	male	0.57459	0.57459
8	SM15	SM	male	0.60732	0.60732
7	SM13	SM	male	0.63767	0.63767
15	SM14	SM	female	0.65852	0.65852
5	SM9	SM	male	0.67811	0.67811
17	SM17	SM	male	0.68678	0.68678
19	SM19	SM	male	0.72354	0.72354
12	SM8	SM	female	0.72425	0.72425
16	SM16	SM	female	0.74245	0.74245
6	SM11	SM	male	0.80465	0.80465
20	SM20	SM	female	0.8134	0.8134
14	SM12	SM	female	0.85642	0.85642
10	SM4	SM	female	0.94823	0.94823
9	SM2	SM	female	1.02643	1.02643
4	SM7	SM	male	1.06411	

Fig1D **DHM PDE11/Actin**

squads	id	group	sex	e11/actin	n no outs	VAR7
14	GH12	GH	female	0.1101	0.1101	
2	GH3	GH	male	0.33053	0.33053	
10	GH4	GH	female	0.4516	0.4516	
5	GH9	GH	male	0.53123	0.53123	
9	GH2	GH	female	0.57303	0.57303	
20	GH20	GH	female	0.62017	0.62017	
12	GH8	GH	female	0.64363	0.64363	
18	GH18	GH	female	0.66485	0.66485	
11	GH6	GH	female	0.6933	0.6933	
8	GH15	GH	male	0.70271	0.70271	
16	GH16	GH	female	0.73265	0.73265	
15	GH14	GH	female	0.75334	0.75334	
7	GH13	GH	male	0.85916	0.85916	
13	GH10	GH	female	0.87453	0.87453	
17	GH17	GH	male	0.93066	0.93066	
1	GH1	GH	male	1.01825	1.01825	
4	GH7	GH	male	1.08352	1.08352	
6	GH11	GH	male	1.30979	1.30979	
3	GH5	GH	male	1.3878	1.3878	
19	GH19	GH	male	1.72579		
4	SH7	SH	male	0.07392	0.07392	
14	SH12	SH	female	0.11478	0.11478	
6	SH11	SH	male	0.15539	0.15539	
2	SH3	SH	male	0.3036	0.3036	
3	SH5	SH	male	0.32032	0.32032	
5	SH9	SH	male	0.42738	0.42738	
19	SH19	SH	male	0.46363	0.46363	
18	SH18	SH	female	0.46937	0.46937	
20	SH20	SH	female	0.53325	0.53325	
12	SH8	SH	female	0.61126	0.61126	
10	SH4	SH	female	0.66627	0.66627	
7	SH13	SH	male	0.66862	0.66862	
15	SH14	SH	female	0.72915	0.72915	
13	SH10	SH	female	0.75716	0.75716	
1	SH1	SH	male	0.75817	0.75817	
16	SH16	SH	female	0.77057	0.77057	
17	SH17	SH	male	0.80015	0.80015	
11	SH6	SH	female	1.00313	1.00313	
9	SH2	SH	female	1.74364		
14	SD12	SD	female	0.09128	0.09128	
6	SD11	SD	male	0.10031	0.10031	
4	SD7	SD	male	0.10138	0.10138	
19	SD19	SD	male	0.21902	0.21902	
2	SD3	SD	male	0.28955	0.28955	
13	SD10	SD	female	0.37024	0.37024	
7	SD13	SD	male	0.39775	0.39775	
16	SD16	SD	female	0.4744	0.4744	
20	SD20	SD	female	0.48035	0.48035	
15	SD14	SD	female	0.4946	0.4946	
12	SD8	SD	female	0.51359	0.51359	
11	SD6	SD	female	0.52842	0.52842	
10	SD4	SD	female	0.60513	0.60513	
1	SD1	SD	male	0.6532	0.6532	
8	SD15	SD	male	0.72104	0.72104	
9	SD2	SD	female	0.75573	0.75573	
17	SD17	SD	male	0.81525	0.81525	
18	SD18	SD	female	0.86472	0.86472	
5	SD9	SD	male	0.92211	0.92211	
3	SD5	SD	male	1.03227	1.03227	
14	SM12	SM	female	0.11028	0.11028	
6	SM11	SM	male	0.19363	0.19363	
3	SM5	SM	male	0.21671	0.21671	
2	SM3	SM	male	0.25794	0.25794	
12	SM8	SM	female	0.4186	0.4186	
19	SM19	SM	male	0.51621	0.51621	
18	SM18	SM	female	0.55404	0.55404	
9	SM2	SM	female	0.55616	0.55616	
4	SM7	SM	male	0.57634	0.57634	
10	SM4	SM	female	0.68787	0.68787	
16	SM16	SM	female	0.77926	0.77926	
15	SM14	SM	female	0.77974	0.77974	
17	SM17	SM	male	0.91283	0.91283	
8	SM15	SM	male	1.15062	1.15062	
7	SM13	SM	male	1.25204	1.25204	
20	SM20	SM	female	1.36103	1.36103	
5	SM9	SM	male	1.58639	1.58639	
1	SM1	SM	male	1.60164	1.60164	
11	SM6	SM	female	1.66595	1.66595	
13	SM10	SM	female	1.73158	1.73158	
8	Shnonexist	SH	male			

squad	ids	groups	sexes	11/actins	n no outs
14	GH12	GH	female	0.1101	0.1101
2	GH3	GH	male	0.33053	0.33053
10	GH4	GH	female	0.4516	0.4516
5	GH9	GH	male	0.53123	0.53123
9	GH2	GH	female	0.57303	0.57303
20	GH20	GH	female	0.62017	0.62017
12	GH8	GH	female	0.64363	0.64363
18	GH18	GH	female	0.66485	0.66485
11	GH6	GH	female	0.6933	0.6933
8	GH15	GH	male	0.70271	0.70271
16	GH16	GH	female	0.73265	0.73265
15	GH14	GH	female	0.75334	0.75334
7	GH13	GH	male	0.85916	0.85916
13	GH10	GH	female	0.87453	0.87453
17	GH17	GH	male	0.93066	0.93066
1	GH1	GH	male	1.01825	1.01825
4	GH7	GH	male	1.08352	1.08352
6	GH11	GH	male	1.30979	1.30979
3	GH5	GH	male	1.3878	1.3878
19	GH19	GH	male	1.72579	
6	SW11	SW	male	0.06066	0.06066
14	SW12	SW	female	0.09594	0.09594
8	SW15	SW	male	0.27384	0.27384
19	SW19	SW	male	0.35109	0.35109
7	SW13	SW	male	0.39321	0.39321
17	SW17	SW	male	0.39757	0.39757
11	SW6	SW	female	0.49921	0.49921
9	SW2	SW	female	0.50659	0.50659
18	SW18	SW	female	0.55132	0.55132
12	SW8	SW	female	0.55512	0.55512
10	SW4	SW	female	0.5862	0.5862
16	SW16	SW	female	0.59124	0.59124
2	SW3	SW	male	0.69345	0.69345
3	SW5	SW	male	0.6939	0.6939
15	SW14	SW	female	1.02725	1.02725
20	SW20	SW	female	1.0331	1.0331
1	SW1	SW	male	1.22588	1.22588
4	SW7	SW	male	1.64605	1.64605
5	SW9	SW	male	1.84532	1.84532
13	SW10	SW	female	2.87632	

Fig1G **DHC PDE11/Actin**

squads	id	group	sex	e11/actin n	no outs
9	GH2	GH	female	0.20007	0.20007
18	GH18	GH	female	0.24256	0.24256
16	GH16	GH	female	0.26289	0.26289
15	GH14	GH	female	0.27684	0.27684
6	GH11	GH	male	0.28244	0.28244
20	GH20	GH	female	0.28835	0.28835
12	GH8	GH	female	0.31566	0.31566
10	GH4	GH	female	0.41263	0.41263
4	GH7	GH	male	0.42642	0.42642
17	GH17	GH	male	0.43099	0.43099
1	GH1	GH	male	0.43671	0.43671
5	GH9	GH	male	0.44248	0.44248
14	GH12	GH	female	0.45363	0.45363
2	GH3	GH	male	0.45417	0.45417
8	GH15	GH	male	0.45552	0.45552
19	GH19	GH	male	0.47059	0.47059
3	GH5	GH	male	0.49178	0.49178
7	GH13	GH	male	0.50309	0.50309
11	GH6	GH	female	0.55407	0.55407
13	GH10	GH	female	0.67068	
12	SH8	SH	female	0.19072	0.19072
17	SH17	SH	male	0.2535	0.2535
11	SH6	SH	female	0.2931	0.2931
1	SH1	SH	male	0.32147	0.32147
6	SH11	SH	male	0.32243	0.32243
4	SH7	SH	male	0.35282	0.35282
3	SH5	SH	male	0.35315	0.35315
14	SH12	SH	female	0.38115	0.38115
20	SH20	SH	female	0.40063	0.40063
9	SH2	SH	female	0.40103	0.40103
15	SH14	SH	female	0.41149	0.41149
2	SH3	SH	male	0.43415	0.43415
18	SH18	SH	female	0.51612	0.51612
13	SH10	SH	female	0.53032	0.53032
7	SH13	SH	male	0.57186	0.57186
10	SH4	SH	female	0.57746	0.57746
5	SH9	SH	male	0.61309	0.61309
16	SH16	SH	female	0.95455	
19	SH19	SH	male	0.9731	
17	SD17	SD	male	0.24262	0.24262
15	SD14	SD	female	0.26343	0.26343
14	SD12	SD	female	0.31502	0.31502
8	SD15	SD	male	0.33195	0.33195
9	SD2	SD	female	0.36404	0.36404
20	SD20	SD	female	0.36751	0.36751
1	SD1	SD	male	0.37883	0.37883
13	SD10	SD	female	0.41947	0.41947
11	SD6	SD	female	0.43427	0.43427
4	SD7	SD	male	0.46263	0.46263
10	SD4	SD	female	0.46805	0.46805
19	SD19	SD	male	0.4886	0.4886
7	SD13	SD	male	0.48909	0.48909
3	SD5	SD	male	0.51036	0.51036
6	GD11	SD	male	0.52591	0.52591
2	SD3	SD	male	0.57133	0.57133
18	SD18	SD	female	0.61458	0.61458
12	SD8	SD	female	0.66303	0.66303
5	SD9	SD	male	0.71148	0.71148
16	SD16	SD	female	0.90618	
4	SW7	SW	male	0.17939	
14	SW12	SW	female	0.20751	0.20751
20	SW20	SW	female	0.28544	0.28544
15	SW14	SW	female	0.29965	0.29965
6	SW11	SW	male	0.30534	0.30534
19	SW19	SW	male	0.37118	0.37118
8	SW15	SW	male	0.39357	0.39357
7	SW13	SW	male	0.43109	0.43109
1	SW1	SW	male	0.45236	0.45236
5	SW9	SW	male	0.48336	0.48336
12	SW8	SW	female	0.50102	0.50102
16	SW16	SW	female	0.50795	0.50795
2	SW3	SW	male	0.51408	0.51408
17	SW17	SW	male	0.52098	0.52098
13	SW10	SW	female	0.53032	0.53032
10	SW4	SW	female	0.53931	0.53931
9	SW2	SW	female	0.55677	0.55677
11	SW6	SW	female	0.58437	0.58437
3	SW5	SW	male	0.63093	0.63093
18	SW18	SW	female	0.66268	0.66268
17	SM17	SM	male	0.26187	0.26187
15	SM14	SM	female	0.26672	0.26672
16	SM16	SM	female	0.28484	0.28484
14	SM12	SM	female	0.29808	0.29808
4	SM7	SM	male	0.30823	0.30823
20	SM20	SM	female	0.33521	0.33521
6	SM11	SM	male	0.34099	0.34099
2	SM3	SM	male	0.38607	0.38607
12	SM8	SM	female	0.39495	0.39495
7	SM13	SM	male	0.39948	0.39948
5	SM9	SM	male	0.40704	0.40704
8	SM15	SM	male	0.41546	0.41546
19	SM19	SM	male	0.42111	0.42111
10	SM4	SM	female	0.45535	0.45535
9	SM2	SM	female	0.45984	0.45984
1	SM1	SM	male	0.48539	0.48539
11	SM6	SM	female	0.50337	0.50337
13	SM10	SM	female	0.62838	0.62838
3	SM5	SM	male	0.71883	
18	SM18	SM	female	0.72146	

Fig1F **DHN PDE11/Actin**

squads	id	group	sex	e11/actin	e11/actin
7	GH13	GH	male	0.451	0.451
8	GH15	GH	male	0.6033	0.6033
3	GH5	GH	male	0.6799	0.6799
17	GH17	GH	male	0.9193	0.9193
19	GH19	GH	male	1.0038	1.0038
5	GH9	GH	male	1.0894	1.0894
1	GH1	GH	male	1.108	1.108
4	GH7	GH	male	1.1663	1.1663
2	GH3	GH	male	1.1832	1.1832
6	GH11	GH	male	1.4989	
17	SH17	SH	male	0.5407	0.5407
3	SH5	SH	male	0.637	0.637
2	SH3	SH	male	1.0485	1.0485
7	SH13	SH	male	1.1147	1.1147
5	SH9	SH	male	1.182	1.182
6	SH11	SH	male	1.2616	1.2616
4	SH7	SH	male	1.8201	1.8201
1	SH1	SH	male	1.8361	1.8361
19	SH19	SH	male	2.0757	
17	SD17	SD	male	0.5175	0.5175
6	SD11	SD	male	0.5283	0.5283
5	SD9	SD	male	0.5975	0.5975
3	SD5	SD	male	0.7256	0.7256
2	SD3	SD	male	0.7447	0.7447
1	SD1	SD	male	0.9307	0.9307
4	SD7	SD	male	1.0356	1.0356
19	SD19	SD	male	1.0422	1.0422
7	SD13	SD	male	1.2266	1.2266
8	SD15	SD	male	1.4104	1.4104
6	SW11	SW	male	0.2532	0.2532
3	SW5	SW	male	0.5504	0.5504
2	SW3	SW	male	0.5592	0.5592
1	SW1	SW	male	0.708	0.708
5	SW9	SW	male	0.7608	0.7608
19	SW19	SW	male	0.7917	0.7917
8	SW15	SW	male	0.8391	0.8391
7	SW13	SW	male	0.896	0.896
4	SW7	SW	male	0.9687	0.9687
17	SW17	SW	male	1.1113	1.1113
8	SM15	SM	male	0.5329	0.5329
17	SM17	SM	male	0.5586	0.5586
3	SM5	SM	male	0.5621	0.5621
6	SM11	SM	male	0.6188	0.6188
2	SM3	SM	male	0.6229	0.6229
1	SM1	SM	male	0.718	0.718
19	SM19	SM	male	0.8983	0.8983
4	SM7	SM	male	1.3134	1.3134
7	SM13	SM	male	1.4393	1.4393
5	SM9	SM	male	1.6688	1.6688

Fig1H **VHM PDE2/Actin**

squads	id	group	sex	de2/actin	n no outs
6	GH11	GH	male	0.43721	0.43721
12	GH8	GH	female	0.57738	0.57738
10	GH4	GH	female	0.6243	0.6243
2	GH3	GH	male	0.76599	0.76599
3	GH5	GH	male	0.85815	0.85815
16	GH16	GH	female	0.87648	0.87648
4	GH7	GH	male	0.92136	0.92136
19	GH19	GH	male	1.06823	1.06823
20	GH20	GH	female	1.11566	1.11566
7	GH13	GH	male	1.17735	1.17735
17	GH17	GH	male	1.22386	1.22386
13	GH10	GH	female	1.22514	1.22514
15	GH14	GH	female	1.26345	1.26345
18	GH18	GH	female	1.38085	1.38085
9	GH2	GH	female	1.40097	1.40097
8	GH15	GH	male	1.55495	1.55495
1	GH1	GH	male	1.6294	1.6294
11	GH6	GH	female	1.88055	1.88055
14	GH12	GH	female	2.22519	
5	GH9	GH	male	2.47033	
18	SH18	SH	female	0.60105	0.60105
1	SH1	SH	male	0.65558	0.65558
12	SH8	SH	female	0.70569	0.70569
2	SH3	SH	male	0.73424	0.73424
14	SH12	SH	female	0.74191	0.74191
10	SH4	SH	female	0.75479	0.75479
19	SH19	SH	male	0.79228	0.79228
6	SH11	SH	male	0.84288	0.84288
13	SH10	SH	female	0.84654	0.84654
9	SH2	SH	female	0.8587	0.8587
15	SH14	SH	female	0.94895	0.94895
16	SH16	SH	female	0.94898	0.94898
20	SH20	SH	female	1.04716	1.04716
3	SH5	SH	male	1.08954	1.08954
4	SH7	SH	male	1.09025	1.09025
7	SH13	SH	male	1.18376	1.18376
5	SH9	SH	male	1.20911	1.20911
17	SH17	SH	male	1.33604	1.33604
11	SH6	SH	female	2.43777	
2	SD3	SD	male	0.47882	0.47882
4	SD7	SD	male	0.54339	0.54339
12	SD8	SD	female	0.62384	0.62384
11	SD6	SD	female	0.62762	0.62762
10	SD4	SD	female	0.65976	0.65976
14	SD12	SD	female	0.67621	0.67621
19	SD19	SD	male	0.80908	0.80908
20	SD20	SD	female	0.81718	0.81718
6	SD11	SD	male	0.87301	0.87301
13	SD10	SD	female	0.92036	0.92036
18	SD18	SD	female	1.03052	1.03052
16	SD16	SD	female	1.06262	1.06262
15	SD14	SD	female	1.08747	1.08747
8	SD15	SD	male	1.15123	1.15123
7	SD13	SD	male	1.21509	1.21509
5	SD9	SD	male	1.35314	1.35314
17	SD17	SD	male	1.44316	1.44316
9	SD2	SD	female	1.57755	1.57755
1	SD1	SD	male	1.837	1.837
3	SD5	SD	male	2.36347	
3	SW5	SW	male	0.49044	0.49044
2	SW3	SW	male	0.56936	0.56936
10	SW4	SW	female	0.65653	0.65653
5	SW9	SW	male	0.75159	0.75159
11	SW6	SW	female	0.78086	0.78086
6	SW11	SW	male	0.79545	0.79545
8	SW15	SW	male	0.84138	0.84138
19	SW19	SW	male	0.96828	0.96828
16	SW16	SW	female	1.00714	1.00714
14	SW12	SW	female	1.02677	1.02677
7	SW13	SW	male	1.07823	1.07823
15	SW14	SW	female	1.09064	1.09064
13	SW10	SW	female	1.09188	1.09188
1	SW1	SW	male	1.11185	1.11185
18	SW18	SW	female	1.13706	1.13706
12	SW8	SW	female	1.33085	1.33085
17	SW17	SW	male	1.33782	1.33782
4	SW7	SW	male	1.38075	1.38075
20	SW20	SW	female	1.51935	1.51935
9	SW2	SW	female	1.59668	1.59668
8	SM15	SM	male	0.44723	0.44723
10	SM4	SM	female	0.45441	0.45441
2	SM3	SM	male	0.52372	0.52372
12	SM8	SM	female	0.60245	0.60245
6	SM11	SM	male	0.62304	0.62304
3	SM5	SM	male	0.63577	0.63577
19	SM19	SM	male	0.6361	0.6361
16	SM16	SM	female	0.7808	0.7808
14	SM12	SM	female	0.87789	0.87789
18	SM18	SM	female	0.91786	0.91786
7	SM13	SM	male	0.95515	0.95515
13	SM10	SM	female	1.03961	1.03961
17	SM17	SM	male	1.05667	1.05667
11	SM6	SM	female	1.1045	1.1045
20	SM20	SM	female	1.10483	1.10483
4	SM7	SM	male	1.29838	1.29838
5	SM9	SM	male	1.31576	1.31576
15	SM14	SM	female	1.60498	1.60498
9	SM2	SM	female	2.08783	
1	SM1	SM	male	2.36554	

Fig1I **VHM PDE10/Actin**

squads	id	group	sex	e10/actin n	no outs
6	GH11	GH	male	0.05777	0.05777
8	GH15	GH	male	0.13071	0.13071
2	GH3	GH	male	0.19085	0.19085
4	GH7	GH	male	0.19358	0.19358
14	GH12	GH	female	0.43832	0.43832
16	GH16	GH	female	0.52889	0.52889
12	GH8	GH	female	0.58456	0.58456
17	GH17	GH	male	0.59737	0.59737
20	GH20	GH	female	0.73034	0.73034
19	GH19	GH	male	0.77254	0.77254
10	GH4	GH	female	1.01846	1.01846
18	GH18	GH	female	1.05448	1.05448
1	GH1	GH	male	1.08149	1.08149
9	GH2	GH	female	1.10211	1.10211
5	GH9	GH	male	1.34533	1.34533
15	GH14	GH	female	1.36373	1.36373
3	GH5	GH	male	1.62726	1.62726
13	GH10	GH	female	1.75384	1.75384
7	GH13	GH	male	1.77999	1.77999
11	GH6	GH	female	2.40304	
4	SH7	SH	male	-0.0693	-0.0693
14	SH12	SH	female	0.33182	0.33182
12	SH8	SH	female	0.38611	0.38611
3	SH5	SH	male	0.4273	0.4273
17	SH17	SH	male	0.51884	0.51884
2	SH3	SH	male	0.60451	0.60451
9	SH2	SH	female	0.62983	0.62983
18	SH18	SH	female	0.63336	0.63336
16	SH16	SH	female	0.71899	0.71899
13	SH10	SH	female	0.78529	0.78529
10	SH4	SH	female	0.81997	0.81997
19	SH19	SH	male	0.8375	0.8375
15	SH14	SH	female	0.9173	0.9173
20	SH20	SH	female	0.92819	0.92819
7	SH13	SH	male	1.05122	1.05122
6	SH11	SH	male	1.11405	1.11405
1	SH1	SH	male	1.20031	1.20031
11	SH6	SH	female	1.37146	1.37146
5	SH9	SH	male	1.9686	
4	SD7	SD	male	0.09488	0.09488
2	SD3	SD	male	0.11388	0.11388
8	SD15	SD	male	0.20425	0.20425
9	SD2	SD	female	0.25384	0.25384
14	SD12	SD	female	0.37432	0.37432
3	SD5	SD	male	0.54939	0.54939
11	SD6	SD	female	0.55828	0.55828
12	SD8	SD	female	0.55991	0.55991
10	SD4	SD	female	0.64069	0.64069
18	SD18	SD	female	0.7639	0.7639
5	SD9	SD	male	0.77484	0.77484
15	SD14	SD	female	0.82517	0.82517
17	SD17	SD	male	0.8253	0.8253
16	SD16	SD	female	0.8486	0.8486
19	SD19	SD	male	0.90996	0.90996
7	SD13	SD	male	0.91075	0.91075
20	SD20	SD	female	0.92749	0.92749
6	SD11	SD	male	1.14237	1.14237
13	SD10	SD	female	1.31639	1.31639
1	SD1	SD	male	3.12563	
5	SW9	SW	male	0.14262	0.14262
8	SW15	SW	male	0.24275	0.24275
14	SW12	SW	female	0.32167	0.32167
6	SW11	SW	male	0.37565	0.37565
2	SW3	SW	male	0.42188	0.42188
18	SW18	SW	female	0.47866	0.47866
12	SW8	SW	female	0.50817	0.50817
11	SW6	SW	female	0.53164	0.53164
10	SW4	SW	female	0.68147	0.68147
17	SW17	SW	male	0.74444	0.74444
16	SW16	SW	female	0.77412	0.77412
1	SW1	SW	male	0.79481	0.79481
15	SW14	SW	female	0.8663	0.8663
4	SW7	SW	male	0.90789	0.90789
20	SW20	SW	female	1.00496	1.00496
13	SW10	SW	female	1.05647	1.05647
3	SW5	SW	male	1.06674	1.06674
19	SW19	SW	male	1.0913	1.0913
9	SW2	SW	female	1.17784	1.17784
7	SW13	SW	male	1.2246	1.2246
1	SM1	SM	male	0.15717	0.15717
2	SM3	SM	male	0.26227	0.26227
6	SM11	SM	male	0.28919	0.28919
8	SM15	SM	male	0.34918	0.34918
14	SM12	SM	female	0.35512	0.35512
4	SM7	SM	male	0.40529	0.40529
12	SM8	SM	female	0.47969	0.47969
16	SM16	SM	female	0.50831	0.50831
10	SM4	SM	female	0.54108	0.54108
11	SM6	SM	female	0.56994	0.56994
15	SM14	SM	female	0.6014	0.6014
18	SM18	SM	female	0.60241	0.60241
5	SM9	SM	male	0.74238	0.74238
17	SM17	SM	male	0.82216	0.82216
20	SM20	SM	female	0.82901	0.82901
19	SM19	SM	male	0.8334	0.8334
9	SM2	SM	female	1.08752	1.08752
13	SM10	SM	female	1.21957	1.21957
7	SM13	SM	male	1.26406	1.26406
3	SM5	SM	male	2.74974	

Fig2 **VH M PDE11/Actin**

squads	ID	Housing	Sex	hm Actin	hm actin	11/Actin	11/actin	n no outs	n no outs	VAR11	ID	Region	Housing	Sex	cd no gh out no s
1	GH1	GH	Male	1.84946	0.54621	0.40969	0.26741	0.40969	0.26741		GH1	VH M	GH	Male	0.214343189
2	GH2	GH	Female	1.20072	0.35461	0.6062	0.39568	0.6062	0.39568		GH8	VH M	GH	Female	0.285071256
3	GH3	GH	Male	0.10394	0.0307	2.18114	1.42367	2.18114	1.42367		GH2	VH M	GH	Female	0.317154364
4	GH4	GH	Female	1.71685	0.50704	0.81316	0.53077	0.81316	0.53077		GH10	VH M	GH	Female	0.321474869
5	GH5	GH	Male	0.74552	0.22018	1.33644	0.87232	1.33644	0.87232		GH6	VH M	GH	Female	0.341838663
6	GH6	GH	Female	0.38351	0.11326	0.65338	0.42647	0.65338	0.42647		GH7	VH M	GH	Male	0.403927339
7	GH7	GH	Male	0.69947	0.20658	0.48822	0.31867	0.48822	0.31867		GH4	VH M	GH	Female	0.425437739
8	GH8	GH	Female	2.56364	0.75713	0.34456	0.2249	0.34456	0.2249		GH9	VH M	GH	Male	0.552844499
9	GH9	GH	Male	0.66096	0.1952	0.66821	0.43615	0.66821	0.43615		GH5	VH M	GH	Male	0.699211889
10	GH10	GH	Female	1.61711	0.47759	0.38856	0.25362	0.38856	0.25362		GH11	VH M	GH	Male	1.052625359
11	GH11	GH	Male	0.37219	0.10992	1.27228	0.83045	1.27228	0.83045		GH3	VH M	GH	Male	1.141146143
12	GH12	GH	Female	0.08663	0.02559	2.83817	1.85253				GH12	VH M	GH	Female	
1	SM1	SM	Male	1.24731	0.36837	0.96142	0.62754	0.96142	0.62754		SM10	VH M	SM	Female	0.195680355
2	SM2	SM	Female	1.7491	0.51657	0.67878	0.44306	0.67878	0.44306		SM9	VH M	SM	Male	0.217324458
3	SM3	SM	Male	0.12186	0.03599	4.9447	3.22751	4.9447	3.22751		SM8	VH M	SM	Female	0.345318274
4	SM4	SM	Female	0.49104	0.14502	1.0692	0.69789	1.0692	0.69789		SM2	VH M	SM	Female	0.355131276
5	SM5	SM	Male	0.8853	0.26146	0.88282	0.57623	0.88282	0.57623		SM5	VH M	SM	Male	0.461880907
6	SM6	SM	Female	0.82796	0.24452	2.41396	1.57564	2.41396	1.57564		SM1	VH M	SM	Male	0.503005208
7	SM7	SM	Male	0.33048	0.0976	1.92885	1.259	1.92885	1.259		SM11	VH M	SM	Male	0.541416112
8	SM8	SM	Female	1.09091	0.32218	0.41738	0.27243	0.41738	0.27243		SM4	VH M	SM	Female	0.55939396
9	SM9	SM	Male	1.42139	0.41978	0.26267	0.17145	0.26267	0.17145		SM6	VH M	SM	Female	1.262956798
10	SM10	SM	Female	1.96364	0.57993	0.23651	0.15438	0.23651	0.15438		SM7	VH M	SM	Male	1.59583979
11	SM11	SM	Male	1.2385	0.36577	0.6544	0.42714	0.6544	0.42714		SM3	VH M	SM	Male	2.587010102
12	SM12	SM	Female	0.04171	0.01232	8.07787	5.27259				SM12	VH M	SM	Female	

Fig2 **VH C PDE11/Actin**

squad	ID	Region	Housing	Sex	vhc Actin	vhc actin	11/Actin	11/actin	n no outs	11/actin no outs	VAR23
12	GH12	VH C	GH	Female	0.84054	0.12405	0.58943	0.37218	0.58943	0.372179453	
5	GH5	VH C	GH	Male	1.14545	0.16905	0.60745	0.38355	0.60745	0.383554523	
1	GH1	VH C	GH	Male	0.97091	0.14329	0.68549	0.43283	0.68549	0.432833878	
4	GH4	VH C	GH	Female	1.15091	0.16986	0.74476	0.47026	0.74476	0.470255391	
10	GH10	VH C	GH	Female	1.27869	0.18871	0.77492	0.4893	0.77492	0.489298861	
9	GH9	VH C	GH	Male	1.37705	0.20323	0.95342	0.60201	0.95342	0.602012349	
2	GH2	VH C	GH	Female	1.04182	0.15376	0.96793	0.61117	0.96793	0.611172698	
11	GH11	VH C	GH	Male	1.07303	0.15836	0.98116	0.61952	0.98116	0.619523714	
8	GH8	VH C	GH	Female	0.71535	0.10557	1.28128	0.80903	1.28128	0.809025086	
7	GH7	VH C	GH	Male	0.71535	0.10557	1.41979	0.89649	1.41979	0.896487257	
6	GH6	VH C	GH	Female	0.62182	0.09177	1.47576	0.93182	1.47576	0.931823919	
3	GH3	VH C	GH	Male	1.06909	0.15778	1.51862	0.95889	1.51862	0.958886309	
4	SM4	VH C	SM	Female	2.02909	0.29946	0.65601	0.41422	0.65601	0.414217368	
5	SM5	VH C	SM	Male	1.03091	0.15215	0.76298	0.48176	0.76298	0.481759305	
10	SM10	VH C	SM	Female	0.81371	0.12009	0.89807	0.56706	0.89807	0.56706243	
1	SM1	VH C	SM	Male	1.16727	0.17227	1.00213	0.63276	1.00213	0.632763659	
3	SM3	VH C	SM	Male	0.80727	0.11914	1.0368	0.65466	1.0368	0.65465681	
2	SM2	VH C	SM	Female	1.13455	0.16744	1.06659	0.67347	1.06659	0.6734653	
7	SM7	VH C	SM	Male	0.84948	0.12537	1.10813	0.6997	1.10813	0.699697371	
8	SM8	VH C	SM	Female	1.37705	0.20323	1.24125	0.78375	1.24125	0.783751926	
11	SM11	VH C	SM	Male	0.8763	0.12933	1.34276	0.84785	1.34276	0.84784758	
9	SM9	VH C	SM	Male	0.65276	0.09634	1.49901	0.94651	1.49901	0.94650843	
12	SM12	VH C	SM	Female	0.48286	0.07126	1.87254	1.18236	1.87254	1.182358984	
6	SM6	VH C	SM	Female	0.71455	0.10546	2.41325	1.52378			

Fig2 **VH N PDE11/Actin**

squands	ID	Region	Housing	Sex	vhn Actin	vhn actin	11/Actin	11/actin	n n oouts	11/actin no outs	VAR35
1	GH1	VH N	GH	Male	2.09002	0.41644	0.40711	0.28441	0.40711	0.284407771	
7	GH7	VH N	GH	Male	0.75383	0.1502	0.56921	0.39765	0.56921	0.397645272	
6	GH6	VH N	GH	Female	1.26223	0.2515	0.59979	0.41901	0.59979	0.419013214	
10	GH10	VH N	GH	Female	2.86194	0.57024	0.62617	0.43744	0.62617	0.437442038	
12	GH12	VH N	GH	Female	0.43389	0.08645	0.85319	0.59603	0.85319	0.596034743	
3	GH3	VH N	GH	Male	0.44031	0.08773	0.98904	0.69094	0.98904	0.690939194	
4	GH4	VH N	GH	Female	1.13307	0.22576	1.05842	0.73941	1.05842	0.73940683	
8	GH8	VH N	GH	Female	0.93353	0.18601	1.0725	0.74924	1.0725	0.749240857	
11	GH11	VH N	GH	Male	0.58729	0.11702	1.17473	0.82066	1.17473	0.820659777	
2	GH2	VH N	GH	Female	0.58121	0.11581	1.38327	0.96635	1.38327	0.966348523	
5	GH5	VH N	GH	Male	0.49315	0.09826	1.56236	1.09146	1.56236	1.091456145	
9	GH9	VH N	GH	Male	0.42951	0.08558	1.7042	1.19055	1.7042	1.19054899	
6	SM6	VH N	SM	Female	1.29746	0.25852	0.62998	0.4401	0.62998	0.440103977	
11	SM11	VH N	SM	Male	0.71877	0.14322	0.66721	0.46611	0.66721	0.466106438	
3	SM3	VH N	SM	Male	0.87476	0.1743	0.94972	0.66347	0.94972	0.663472845	
12	SM12	VH N	SM	Female	0.2542	0.05065	0.95984	0.67054	0.95984	0.670539086	
10	SM10	VH N	SM	Female	0.73192	0.14584	0.98858	0.69062	0.98858	0.690615107	
2	SM2	VH N	SM	Female	0.66928	0.13335	1.08114	0.75528	1.08114	0.755277662	
5	SM5	VH N	SM	Male	0.92172	0.18365	1.13393	0.79216	1.13393	0.792159586	
4	SM4	VH N	SM	Female	0.51076	0.10177	1.28549	0.89804	1.28549	0.898037679	
7	SM7	VH N	SM	Male	0.80205	0.15981	1.32175	0.92337	1.32175	0.923365299	
9	SM9	VH N	SM	Male	0.63112	0.12575	1.38643	0.96856	1.38643	0.968556458	
1	SM1	VH N	SM	Male	0.52838	0.10528	1.40748	0.98326	1.40748	0.983259622	
8	SM8	VH N	SM	Female	0.36377	0.07248	1.87342	1.30876			

Fig 3B 3C pCamkii

squads	id	group	sex	kiia/actin	n no outs	kiib/actin	n no outs	kiia/actin	n no outs	iia/cama	a no outs	VAR13
5	GH9	GH	male	1.79692	1.79692	0.91994	0.91994	1.35251	1.35251	0.90809	0.90809	
12	GH8	GH	female	2.3339	2.3339	1.079	1.079	0.14216	0.14216	0.07322	0.07322	
4	GH7	GH	male	2.16496	2.16496	1.12268	1.12268	2.65052	2.65052	1.59585	1.59585	
11	GH6	GH	female	1.49159	1.49159	0.68393	0.68393	2.15377	2.15377	1.73588	1.73588	
3	GH5	GH	male	1.80912	1.80912	0.72159	0.72159	0.94579	0.94579	0.68145	0.68145	
10	GH4	GH	female	2.6769	2.6769	0.98248	0.98248	0.7889	0.7889	0.325	0.325	
2	GH3	GH	male	1.06896		0.78587	0.78587	3.44913	3.44913	3.29886	3.29886	
20	GH20	GH	female	2.35353	2.35353	0.93266	0.93266	5.21244	5.21244	2.88074	2.88074	
9	GH2	GH	female	1.75755	1.75755	1.37188	1.37188	4.52408	4.52408	2.83868	2.83868	
19	GH19	GH	male	2.4601	2.4601	1.43696		3.74844	3.74844	1.8978	1.8978	
18	GH18	GH	female	1.59676	1.59676	1.00788	1.00788	1.91405	1.91405	1.55918	1.55918	
17	GH17	GH	male	2.59307	2.59307	1.38696	1.38696	1.30909	1.30909	0.62879	0.62879	
16	GH16	GH	female	1.77066	1.77066	1.15196	1.15196	5.60938	5.60938	3.37063	3.37063	
8	GH15	GH	male	1.79518	1.79518	0.95218	0.95218	4.42503	4.42503	3.10004	3.10004	
15	GH14	GH	female	1.64037	1.64037	0.65933	0.65933	6.60994		4.28734		
7	GH13	GH	male	2.1204	2.1204	0.89185	0.89185	0	0	0	0	
14	GH12	GH	female	1.59141	1.59141	0.96795	0.96795	1.20456	1.20456	0.9408	0.9408	
6	GH11	GH	male	1.8446	1.8446	0.99463	0.99463	2.42061	2.42061	1.58322	1.58322	
13	GH10	GH	female	1.45474	1.45474	0.75099	0.75099	2.69468	2.69468	2.30236	2.30236	
1	GH1	GH	male	1.36277	1.36277	1.09149	1.09149	1.60689	1.60689	1.20554	1.20554	
5	SH9	SH	male	1.81904	1.81904	0.94104	0.94104	0.04904	0.04904	0.03253	0.03253	
12	SH8	SH	female	2.04167	2.04167	1.14078	1.14078	0.86411	0.86411	0.5088	0.5088	
4	SH7	SH	male	1.22834	1.22834	1.33278	1.33278	0.36122	0.36122	0.38332	0.38332	
11	SH6	SH	female	1.77684	1.77684	0.89403	0.89403	2.66725	2.66725	1.80461	1.80461	
3	SH5	SH	male	1.88589	1.88589	0.83115	0.83115	3.94508		2.72678		
10	SH4	SH	female	1.63148	1.63148	0.82953	0.82953	2.33389	2.33389	1.57759	1.57759	
2	SH3	SH	male	4.19762		1.44158	1.44158	2.70266	2.70266	0.65827	0.65827	
20	SH20	SH	female	2.08036	2.08036	1.1106	1.1106	1.41664	1.41664	0.88574	0.88574	
9	SH2	SH	female	2.8777	2.8777	1.25532	1.25532	1.26003	1.26003	0.48287	0.48287	
19	SH19	SH	male	2.38045	2.38045	1.381	1.381	2.23657	2.23657	1.17024	1.17024	
18	SH18	SH	female	1.53153	1.53153	0.77899	0.77899	0.20806	0.20806	0.1767	0.1767	
17	SH17	SH	male	1.96039	1.96039	0.91951	0.91951	1.55423	1.55423	0.98747	0.98747	
16	SH16	SH	female	3.88328		1.1656	1.1656	0.15265	0.15265	0.04183	0.04183	
15	SH14	SH	female	1.19377	1.19377	0.64374	0.64374	0.15293	0.15293	0.1363	0.1363	
7	SH13	SH	male	1.93671	1.93671	0.70702	0.70702	0.12944	0.12944	0.08405	0.08405	
14	SH12	SH	female									
6	SH11	SH	male	2.78553	2.78553	1.14433	1.14433	0.38825	0.38825	0.16816	0.16816	
13	SH10	SH	female	2.30095	2.30095	1.19734	1.19734	0.10565	0.10565	0.05707	0.05707	
1	SH1	SH	male	1.78493	1.78493	1.10297	1.10297	0.91065	0.91065	0.52161	0.52161	
5	SD9	SD	male	1.90641	1.90641	1.36738	1.36738	1.15091	1.15091	0.72836	0.72836	
12	SD8	SD	female	2.06075	2.06075	1.09377	1.09377	0.27416	0.27416	0.15994	0.15994	
4	SD7	SD	male	2.04535	2.04535	1.15247	1.15247	0.85489	0.85489	0.54482	0.54482	
11	SD6	SD	female	2.06746	2.06746	0.90328	0.90328	0.01142	0.01142	0.00664	0.00664	
3	SD5	SD	male	1.92458	1.92458	0.90629	0.90629	0.7121	0.7121	0.4823	0.4823	
10	SD4	SD	female	1.98669	1.98669	0.81418	0.81418	0.3941	0.3941	0.21876	0.21876	
2	SD3	SD	male	2.82937		1.60916		1.31287	1.31287	0.4744	0.4744	
20	SD20	SD	female	1.80668	1.80668	1.00204	1.00204	0.86704	0.86704	0.62422	0.62422	
9	SD2	SD	female	2.18343	2.18343	0.9987	0.9987	1.02915	1.02915	0.5198	0.5198	
19	SD19	SD	male	1.52227	1.52227	0.73155	0.73155	1.06796	1.06796	0.8738	0.8738	
18	SD18	SD	female	2.13289	2.13289	0.9289	0.9289	0.8253	0.8253	0.5033	0.5033	
17	SD17	SD	male	2.06134	2.06134	0.86995	0.86995	1.86232		1.12527		
16	SD16	SD	female	2.33052	2.33052	0.69012	0.69012	0.1271	0.1271	0.05803	0.05803	
8	SD15	SD	male	1.89076	1.89076	1.44951	1.44951	1.06006	1.06006	0.7051	0.7051	
15	SD14	SD	female	1.45508	1.45508	1.21788	1.21788	0.57111	0.57111	0.41761	0.41761	
7	SD13	SD	male	1.97701	1.97701	0.94707	0.94707	0.91508	0.91508	0.58212	0.58212	
14	SD12	SD	female	3.18746		1.4798	1.4798	0.74086	0.74086	0.2889	0.2889	
6	SD11	SD	male	1.68459	1.68459	0.54174	0.54174	0.63063	0.63063	0.45165	0.45165	
13	SD10	SD	female	1.67132	1.67132	1.08306	1.08306	0.13769	0.13769	0.1024	0.1024	
1	SD1	SD	male	1.62271	1.62271	0.56647	0.56647	0.93352	0.93352	0.58816	0.58816	
5	SW9	SW	male	2.17241	2.17241	1.08927	1.08927	2.28794	2.28794	1.27064	1.27064	
12	SW8	SW	female	2.59204	2.59204	1.44042	1.44042	4.6333	4.6333	2.1489	2.1489	
4	SW7	SW	male	2.66031	2.66031	1.6362	1.6362	2.40969	2.40969	1.1807	1.1807	
11	SW6	SW	female	1.61846	1.61846	0.82474	0.82474	1.11601	1.11601	0.82896	0.82896	
3	SW5	SW	male	2.08159	2.08159	0.74236	0.74236	0.04452	0.04452	0.02788	0.02788	
10	SW4	SW	female	1.93096	1.93096	1.01559	1.01559	0.12411	0.12411	0.07088	0.07088	
2	SW3	SW	male	2.56348	2.56348	1.30015	1.30015	1.13667	1.13667	0.45334	0.45334	
20	SW20	SW	female	2.75017	2.75017	1.81698		1.4194	1.4194	0.67132	0.67132	
9	SW2	SW	female	1.9628	1.9628	1.04463	1.04463	1.25892	1.25892	0.70732	0.70732	
19	SW19	SW	male	1.5604	1.5604	0.77665	0.77665	1.97245	1.97245	1.57442	1.57442	
18	SW18	SW	female	1.51885	1.51885	0.49852	0.49852	0.63711	0.63711	0.54561	0.54561	
17	SW17	SW	male	2.02596	2.02596	0.9633	0.9633	0.80563	0.80563	0.49529	0.49529	
16	SW16	SW	female	2.16147	2.16147	0.83219	0.83219	2.08358	2.08358	1.02564	1.02564	
8	SW15	SW	male	1.44376	1.44376	1.05864	1.05864	2.46383	2.46383	2.14622	2.14622	
15	SW14	SW	female	1.95004	1.95004	1.65793	1.65793	0.03644	0.03644	0.01988	0.01988	
7	SW13	SW	male	2.59028	2.59028	1.13788	1.13788	0.29694	0.29694	0.14417	0.14417	
14	SW12	SW	female	2.6214	2.6214	0.99025	0.99025	4.73903	4.73903	2.24702	2.24702	
6	SW11	SW	male	1.83515	1.83515	0.91025	0.91025	3.34369	3.34369	2.19823	2.19823	
13	SW10	SW	female	2.15758	2.15758	1.1535	1.1535	4.63011	4.63011	2.66732	2.66732	
1	SW1	SW	male	1.64724	1.64724	0.80798	0.80798	0.65386	0.65386	0.40583	0.40583	
5	SM9	SM	male	1.88132	1.88132	1.02471	1.02471	1.83633	1.83633	1.17762	1.17762	
12	SM8	SM	female	2.47528	2.47528	1.21178	1.21178	1.81538	1.81538	0.88168	0.88168	
4	SM7	SM	male	2.55322	2.55322	1.16963	1.16963	4.06522	4.06522	2.07542	2.07542	
11	SM6	SM	female	1.79021	1.79021	1.0213	1.0213	2.45342	2.45342	1.64754	1.64754	
3	SM5	SM	male	1.89486	1.89486	0.67792	0.67792	0.14197	0.14197	0.09766	0.09766	
10	SM4	SM	female	1.64633	1.64633	0.83289	0.83289	0.01936	0.01936	0.01297	0.01297	
2	SM3	SM	male	1.65719	1.65719	0.75519	0.75519	2.10051	2.10051	1.29589	1.29589	
20	SM20	SM	female	2.67055	2.67055	1.49807		2.854	2.854	1.39007	1.39007	
9	SM2	SM	female	1.59437	1.59437	1.14785	1.14785	4.39841	4.39841	3.04229		
19	SM19	SM	male	1.68648	1.68648	0.78871	0.78871	0.53503	0.53503	0.39514	0.39514	
18	SM18	SM	female	1.80689	1.80689	0.7184	0.7184	0.77693	0.77693	0.55928	0.55928	
17	SM17	SM	male	1.99774	1.99774	1.03845	1.03845	1.03925	1.03925	0.64794	0.64794	
16	SM16	SM	female	2.0319	2.0319	1.19829	1.19829	0.32847	0.32847	0.172	0.172	
8	SM15	SM	male	3.25609		1.33835	1.33835	5.17257		1.99788	1.99788	
15	SM14	SM	female	1.83112	1.83112	1.076	1.076	0.45937	0.45937	0.26692	0.26692	
7	SM13	SM	male	1.2132	1.2132	0.78124	0.78124	0.05494	0.05494	0.05696	0.05696	
14	SM12	SM	female	1.69484	1.69484	0.83758	0.83758	0.04087	0.04087	0.02997	0.02997	
6	SM11	SM	male	2.52224	2.52224	1.35978	1.35978	2.67106	2.67106	1.27766	1.27766	
13	SM10	SM	female	1.54368	1.54368	0.80328	0.80328	0.22444	0.22444	0.18072	0.18072	
1	SM1	SM	male	1.51394	1.51394	0.83218	0.83218	1.3242	1.3242	0.89426	0.89426	
8		SH										

squads	id	group	sex	kiib/actin	in n oout	iib/camb	nb no out
1	GH1	GH	male	0.82481	0.82481	0.87642	0.87642
13	GH10	GH	female	1.03477	1.03477	1.52686	1.52686
6	GH11	GH	male	1.20804	1.20804	1.37326	1.37326
14	GH12	GH	female	2.29067	2.29067	2.62244	2.62244
7	GH13	GH	male	0.64942	0.64942	0.8484	0.8484
15	GH14	GH	female	2.67427	2.67427	3.92348	
8	GH15	GH	male	1.72012	1.72012	2.10478	2.10478
16	GH16	GH	female	1.51427	1.51427	1.27157	1.27157
17	GH17	GH	male	1.19839	1.19839	0.9747	0.9747
18	GH18	GH	female	0.89566	0.89566	0.93575	0.93575
19	GH19	GH	male	2.81437	2.81437	2.20939	2.20939
9	GH2	GH	female	1.82642	1.82642	1.46392	1.46392
20	GH20	GH	female	2.29841	2.29841	2.59493	2.59493
2	GH3	GH	male	2.28642	2.28642	3.37426	3.37426
10	GH4	GH	female	0.73395	0.73395	0.82145	0.82145
3	GH5	GH	male	1.55538	1.55538	2.19511	2.19511
11	GH6	GH	female	0.92159	0.92159	1.52025	1.52025
4	GH7	GH	male	1.70592	1.70592	1.54743	1.54743
12	GH8	GH	female	0.95495	0.95495	0.9985	0.9985
5	GH9	GH	male	0.58711	0.58711	0.72159	0.72159
1	SH1	SH	male	1.02606	1.02606	1.0789	1.0789
13	SH10	SH	female	1.34675	1.34675	1.24642	1.24642
6	SH11	SH	male	0.86139	0.86139	0.8511	0.8511
14	SH12	SH	female				
7	SH13	SH	male	0.64707	0.64707	1.06632	1.06632
15	SH14	SH	female	1.69255	1.69255	2.54332	2.54332
16	SH16	SH	female	1.4625	1.4625	1.21372	1.21372
17	SH17	SH	male	0.83416	0.83416	1.02336	1.02336
18	SH18	SH	female	0.68077	0.68077	0.92022	0.92022
19	SH19	SH	male	1.68139	1.68139	1.37345	1.37345
9	SH2	SH	female	0.72475	0.72475	0.63485	0.63485
20	SH20	SH	female	1.52462	1.52462	1.44553	1.44553
2	SH3	SH	male	2.49983	2.49983	2.01115	2.01115
10	SH4	SH	female	2.68182	2.68182	3.55493	
3	SH5	SH	male	2.49824	2.49824	3.061	3.061
11	SH6	SH	female	1.67791	1.67791	2.11741	2.11741
4	SH7	SH	male	0.72276	0.72276	0.55226	0.55226
12	SH8	SH	female	1.9999	1.9999	1.97786	1.97786
5	SH9	SH	male	0.55467	0.55467	0.66644	0.66644
1	SD1	SD	male	0.44323	0.44323	0.90747	0.90747
13	SD10	SD	female	0.92148	0.92148	0.94281	0.94281
6	SD11	SD	male	0.73689	0.73689	1.53796	1.53796
14	SD12	SD	female	1.28725	1.28725	0.96395	0.96395
7	SD13	SD	male	0.74956	0.74956	0.92213	0.92213
15	SD14	SD	female	0.59152	0.59152	0.46983	0.46983
8	SD15	SD	male	0.77474	0.77474	0.62274	0.62274
16	SD16	SD	female	1.2419	1.2419	1.74073	1.74073
17	SD17	SD	female	1.19288	1.19288	1.54682	1.54682
18	SD18	SD	female	1.45851	1.45851	1.65334	1.65334
19	SD19	SD	male	0.97006	0.97006	1.49588	1.49588
9	SD2	SD	female	0.66924	0.66924	0.73686	0.73686
20	SD20	SD	female	1.52117	1.52117	1.59851	1.59851
2	SD3	SD	male	2.58493		1.86304	1.86304
10	SD4	SD	female	1.92573	1.92573	2.60082	
3	SD5	SD	male	0.63064	0.63064	0.70864	0.70864
11	SD6	SD	female	0.82422	0.82422	1.02945	1.02945
4	SD7	SD	male	0.6784	0.6784	0.59947	0.59947
12	SD8	SD	female	1.96922	1.96922	2.03122	2.03122
5	SD9	SD	male	1.32653	1.32653	1.09688	1.09688
1	SW1	SW	male	0.56536	0.56536	0.81152	0.81152
13	SW10	SW	female	1.59571	1.59571	1.53296	1.53296
6	SW11	SW	male	2.67735	2.67735	3.32566	
14	SW12	SW	female	2.2422	2.2422	2.50913	2.50913
7	SW13	SW	male	1.03416	1.03416	1.05891	1.05891
15	SW14	SW	female	1.17602	1.17602	0.68615	0.68615
8	SW15	SW	male	2.5774	2.5774	2.83663	2.83663
16	SW16	SW	female	1.49997	1.49997	1.74355	1.74355
17	SW17	SW	male	1.10349	1.10349	1.29225	1.29225
18	SW18	SW	female	1.15346	1.15346	2.43639	2.43639
19	SW19	SW	male	1.67786	1.67786	2.43708	2.43708
9	SW2	SW	female	0.84908	0.84908	0.89376	0.89376
20	SW20	SW	female	1.4049	1.4049	0.81418	0.81418
2	SW3	SW	male	1.79531	1.79531	1.60147	1.60147
10	SW4	SW	female	1.16898	1.16898	1.26569	1.26569
3	SW5	SW	male	1.01166	1.01166	1.38782	1.38782
11	SW6	SW	female	0.929	0.929	1.27084	1.27084
4	SW7	SW	male	1.54221	1.54221	0.95988	0.95988
12	SW8	SW	female	1.53475	1.53475	1.20208	1.20208
5	SW9	SW	male	2.84153		2.94951	2.94951
1	SM1	SM	male	0.66925	0.66925	0.93271	0.93271
13	SM10	SM	female	1.03905	1.03905	1.43338	1.43338
6	SM11	SM	male	2.39329	2.39329	1.99003	1.99003
14	SM12	SM	female	0.73592	0.73592	0.97365	0.97365
7	SM13	SM	male	0.82521	0.82521	1.2307	1.2307
15	SM14	SM	female	0.53713	0.53713	0.48287	0.48287
8	SM15	SM	male	3.51613		3.06099	
16	SM16	SM	female	1.49187	1.49187	1.20431	1.20431
17	SM17	SM	male	1.5103	1.5103	1.64064	1.64064
18	SM18	SM	female	1.06316	1.06316	1.55831	1.55831
19	SM19	SM	male	0.8991	0.8991	1.28597	1.28597
9	SM2	SM	female	2.90438	2.90438	2.78229	2.78229
20	SM20	SM	female	1.88134	1.88134	1.32238	1.32238
2	SM3	SM	male	1.18679	1.18679	1.8226	1.8226
10	SM4	SM	female	0.39764	0.39764	0.52498	0.52498
3	SM5	SM	male	1.88191	1.88191	2.82704	2.82704
11	SM6	SM	female	1.5735	1.5735	1.73821	1.73821
4	SM7	SM	male	1.65488	1.65488	1.44088	1.44088
12	SM8	SM	female	1.49695	1.49695	1.39372	1.39372
5	SM9	SM	male	0.69521	0.69521	0.76709	0.76709

Fig 3D 3E pS6

squads	id	group	sex	l S6/actin	n no outs	235/actin	n no outs	56235/S6	6 no outs
5	GH9	GH	male	1.02439	1.02439	0.62137	0.62137	0.80785	0.80785
12	GH8	GH	female	0.72657	0.72657	0.56879	0.56879	0.84907	0.84907
4	GH7	GH	male	1.37232	1.37232	1.1097	1.1097	1.17444	1.17444
11	GH6	GH	female	0.19961	0.19961	0.8283	0.8283	4.50058	
3	GH5	GH	male	0.4593	0.4593	0.72682	0.72682	2.29834	2.29834
10	GH4	GH	female	0.33213	0.33213	0.82916	0.82916	2.79905	2.79905
2	GH3	GH	male	0.96991	0.96991	1.10241	1.10241	1.25429	1.25429
20	GH20	GH	female	0.85863	0.85863	1.22928	1.22928	2.04986	2.04986
9	GH2	GH	female	1.56826	1.56826	1.78583	1.78583	1.27672	1.27672
19	GH19	GH	male	1.34246	1.34246	1.33188	1.33188	1.37618	1.37618
18	GH18	GH	female	0.60587	0.60587	0.98144	0.98144	2.31935	2.31935
17	GH17	GH	male	0.78862	0.78862	0.9704	0.9704	1.70684	1.70684
16	GH16	GH	female	0.5716	0.5716	1.02421	1.02421	2.46666	2.46666
8	GH15	GH	male	1.05852	1.05852	1.60985	1.60985	2.24226	2.24226
15	GH14	GH	female	0.74649	0.74649	1.57482	1.57482	2.90412	2.90412
7	GH13	GH	male	0.66916	0.66916	0.28906	0.28906	0.63688	0.63688
14	GH12	GH	female	2.1222		1.75796	1.75796	1.1165	1.1165
6	GH11	GH	male	0.93614	0.93614	0.94477	0.94477	1.34409	1.34409
13	GH10	GH	female	1.42978	1.42978	0.74706	0.74706	0.70424	0.70424
1	GH1	GH	male	0.35498	0.35498	0.61712	0.61712	1.91843	1.91843
5	SH9	SH	male	1.56193	1.56193	0.75279	0.75279	0.64188	0.64188
12	SH8	SH	female	1.91957		1.10491	1.10491	0.62429	0.62429
4	SH7	SH	male	1.46323	1.46323	1.24552	1.24552	1.23629	1.23629
11	SH6	SH	female	0.86622	0.86622	1.20092	1.20092	1.50367	1.50367
3	SH5	SH	male	1.28778	1.28778	1.67413	1.67413	1.88812	1.88812
10	SH4	SH	female	0.5132	0.5132	0.93174	0.93174	2.03553	2.03553
2	SH3	SH	male	1.48446	1.48446	1.31323	1.31323	0.97625	0.97625
20	SH20	SH	female	1.42317	1.42317	1.21087	1.21087	1.21821	1.21821
9	SH2	SH	female	1.51826	1.51826	1.36107	1.36107	1.00509	1.00509
19	SH19	SH	male	1.05173	1.05173	1.36353	1.36353	1.79834	1.79834
18	SH18	SH	female	0.80633	0.80633	0.44319	0.44319	0.78697	0.78697
17	SH17	SH	male	0.61404	0.61404	0.72043	0.72043	1.62745	1.62745
16	SH16	SH	female	0.65859	0.65859	0.24723	0.24723	0.51676	0.51676
15	SH14	SH	female	0.77025	0.77025	0.43416	0.43416	0.77593	0.77593
7	SH13	SH	male	0.77977	0.77977	0.76079	0.76079	1.43846	1.43846
14	SH12	SH	female						
6	SH11	SH	male	0.90266	0.90266	1.28653	1.28653	1.89818	1.89818
13	SH10	SH	female	0.80844	0.80844	0.53543	0.53543	0.89267	0.89267
1	SH1	SH	male	1.51153	1.51153	1.01784	1.01784	0.7431	0.7431
5	SD9	SD	male	2.04281	2.04281	1.50502	1.50502	0.98119	0.98119
12	SD8	SD	female	2.01108	2.01108	0.95704	0.95704	0.51614	0.51614
4	SD7	SD	male	1.28077	1.28077	1.06992	1.06992	1.21329	1.21329
11	SD6	SD	female	1.24707	1.24707	0.67803	0.67803	0.58969	0.58969
3	SD5	SD	male	1.46861	1.46861	1.51182	1.51182	1.49512	1.49512
10	SD4	SD	female	1.121	1.121	0.84009	0.84009	0.84022	0.84022
2	SD3	SD	male	2.53042		1.84714	1.84714	0.80555	0.80555
20	SD20	SD	female	1.28105	1.28105	1.18333	1.18333	1.32258	1.32258
9	SD2	SD	female	0.80448	0.80448	1.11808	1.11808	1.55823	1.55823
19	SD19	SD	male	1.23758	1.23758	1.20973	1.20973	1.3559	1.3559
18	SD18	SD	female	1.41983	1.41983	0.94179	0.94179	0.94972	0.94972
17	SD17	SD	male	0.79677	0.79677	1.03814	1.03814	1.80733	1.80733
16	SD16	SD	female	0.92896	0.92896	0.34598	0.34598	0.5127	0.5127
8	SD15	SD	male	0.90564	0.90564	1.0667	1.0667	1.73653	1.73653
15	SD14	SD	female	1.15951	1.15951	1.75747	1.75747	2.08652	2.08652
7	SD13	SD	male	0.94547	0.94547	1.19257	1.19257	1.85965	1.85965
14	SD12	SD	female	1.15819	1.15819	2.15741		2.51068	2.51068
6	SD11	SD	male	0.51557	0.51557	0.63304	0.63304	1.63523	1.63523
13	SD10	SD	female	1.20908	1.20908	0.52218	0.52218	0.5821	0.5821
1	SD1	SD	male	0.35918	0.35918	1.05842	1.05842	3.25189	
5	SW9	SW	male	1.45297	1.45297	0.85426	0.85426	0.78302	0.78302
12	SW8	SW	female	1.27201	1.27201	1.74714	1.74714	1.48972	1.48972
4	SW7	SW	male	1.0394	1.0394	1.42661	1.42661	1.99345	1.99345
11	SW6	SW	female	0.70905	0.70905	0.84846	0.84846	1.29783	1.29783
3	SW5	SW	male	0.74139	0.74139	0.48228	0.48228	0.94478	0.94478
10	SW4	SW	female	1.57927	1.57927	0.60031	0.60031	0.42618	0.42618
2	SW3	SW	male	1.86512	1.86512	1.3258	1.3258	0.78444	0.78444
20	SW20	SW	female	1.29285	1.29285	1.51359	1.51359	1.67626	1.67626
9	SW2	SW	female	0.81995	0.81995	1.08941	1.08941	1.48963	1.48963
19	SW19	SW	male	1.42822	1.42822	1.15703	1.15703	1.12373	1.12373
18	SW18	SW	female	0.889	0.889	0.63078	0.63078	1.01591	1.01591
17	SW17	SW	male	1.04892	1.04892	1.19452	1.19452	1.57965	1.57965
16	SW16	SW	female	0.7289	0.7289	0.69975	0.69975	1.32155	1.32155
8	SW15	SW	male	1.52339	1.52339	1.00443	1.00443	0.97209	0.97209
15	SW14	SW	female	2.24732		1.5953	1.5953	0.9772	0.9772
7	SW13	SW	male	1.13881	1.13881	1.00264	1.00264	1.29806	1.29806
14	SW12	SW	female	0.92841	0.92841	1.26907	1.26907	1.84239	1.84239
6	SW11	SW	male	0.74671	0.74671	1.59197	1.59197	2.83939	
13	SW10	SW	female	0.62137	0.62137	1.23624	1.23624	2.68158	
1	SW1	SW	male	0.48576	0.48576	0.89785	0.89785	2.03971	2.03971
5	SM9	SM	male	0.8059	0.8059	0.78083	0.78083	1.29038	1.29038
12	SM8	SM	female	1.33194	1.33194	1.36408	1.36408	1.11076	1.11076
4	SM7	SM	male	1.44138	1.44138	1.05506	1.05506	1.06312	1.06312
11	SM6	SM	female	0.97395	0.97395	1.31176	1.31176	1.46078	1.46078
3	SM5	SM	male	0.70288	0.70288	0.30759	0.30759	0.63558	0.63558
10	SM4	SM	female	2.23233		0.50733	0.50733	0.25481	0.25481
2	SM3	SM	male	1.3014	1.3014	0.9393	0.9393	0.79649	0.79649
20	SM20	SM	female	1.57282	1.57282	1.55882	1.55882	1.41905	1.41905
9	SM2	SM	female	0.76817	0.76817	1.54642	1.54642	2.25708	
19	SM19	SM	male	1.59418	1.59418	0.62192	0.62192	0.54114	0.54114
18	SM18	SM	female	1.10752	1.10752	0.91634	0.91634	1.18463	1.18463
17	SM17	SM	male	1.35454	1.35454	1.00188	1.00188	1.02597	1.02597
16	SM16	SM	female	1.8302	1.8302	1.16353	1.16353	0.87516	0.87516
8	SM15	SM	male	2.03595	2.03595	1.90414	1.90414	1.37889	1.37889
15	SM14	SM	female	1.61524	1.61524	1.76699	1.76699	1.50593	1.50593
7	SM13	SM	male	1.07466	1.07466	0.71831	0.71831	0.98545	0.98545
14	SM12	SM	female	0.63577	0.63577	0.74401	0.74401	1.57731	1.57731
6	SM11	SM	male	1.268	1.268	1.63886	1.63886	1.72133	1.72133
13	SM10	SM	female	1.21812	1.21812	0.57914	0.57914	0.64081	0.64081
1	SM1	SM	male	0.3943	0.3943	0.49035	0.49035	1.37237	1.37237

squads	id	group	sex	56240/S6	S6 no out	VAR27
16	GH16	GH	female	0.07348	0.07348	
12	GH8	GH	female	0.08299	0.08299	
3	GH5	GH	male	0.09942	0.09942	
4	GH7	GH	male	0.1216	0.1216	
15	GH14	GH	female	0.12504	0.12504	
17	GH17	GH	male	0.12718	0.12718	
13	GH10	GH	female	0.13064	0.13064	
18	GH18	GH	female	0.13084	0.13084	
20	GH20	GH	female	0.1378	0.1378	
7	GH13	GH	male	0.13978	0.13978	
10	GH4	GH	female	0.14809	0.14809	
6	GH11	GH	male	0.15482	0.15482	
1	GH1	GH	male	0.15584	0.15584	
19	GH19	GH	male	0.16534	0.16534	
9	GH2	GH	female	0.16561	0.16561	
5	GH9	GH	male	0.17811	0.17811	
8	GH15	GH	male	0.18147	0.18147	
11	GH6	GH	female	0.18636	0.18636	
2	GH3	GH	male	0.20501	0.20501	
14	GH12	GH	female	0.25273		
16	SH16	SH	female	0.06059	0.06059	
13	SH10	SH	female	0.07448	0.07448	
2	SH3	SH	male	0.10084	0.10084	
10	SH4	SH	female	0.10351	0.10351	
18	SH18	SH	female	0.1044	0.1044	
6	SH11	SH	male	0.10824	0.10824	
9	SH2	SH	female	0.10828	0.10828	
7	SH13	SH	male	0.11219	0.11219	
5	SH9	SH	male	0.12331	0.12331	
1	SH1	SH	male	0.12418	0.12418	
12	SH8	SH	female	0.12977	0.12977	
15	SH14	SH	female	0.13407	0.13407	
17	SH17	SH	male	0.14684	0.14684	
11	SH6	SH	female	0.1592	0.1592	
19	SH19	SH	male	0.16765	0.16765	
20	SH20	SH	female	0.2241	0.2241	
3	SH5	SH	male	0.24244	0.24244	
4	SH7	SH	male	0.36404		
14	SH12	SH	female			missing s6
6	SD11	SD	male	0.0822	0.0822	
4	SD7	SD	male	0.08719	0.08719	
16	SD16	SD	female	0.10571	0.10571	
8	SD15	SD	male	0.11526	0.11526	
3	SD5	SD	male	0.11931	0.11931	
11	SD6	SD	female	0.12298	0.12298	
10	SD4	SD	female	0.13217	0.13217	
9	SD2	SD	female	0.13535	0.13535	
12	SD8	SD	female	0.13958	0.13958	
18	SD18	SD	female	0.15033	0.15033	
19	SD19	SD	male	0.17343	0.17343	
2	SD3	SD	male	0.19085	0.19085	
5	SD9	SD	male	0.19318	0.19318	
7	SD13	SD	male	0.19802	0.19802	
13	SD10	SD	female	0.21488	0.21488	
1	SD1	SD	male	0.22168	0.22168	
14	SD12	SD	female	0.22465	0.22465	
20	SD20	SD	female	0.22549	0.22549	
17	SD17	SD	male	0.22661	0.22661	
15	SD14	SD	female	0.2604	0.2604	
14	SW12	SW	female	0.07326	0.07326	
4	SW7	SW	male	0.07801	0.07801	
7	SW13	SW	male	0.08276	0.08276	
16	SW16	SW	female	0.10828	0.10828	
18	SW18	SW	female	0.11971	0.11971	
1	SW1	SW	male	0.12584	0.12584	
2	SW3	SW	male	0.12918	0.12918	
20	SW20	SW	female	0.13502	0.13502	
10	SW4	SW	female	0.13695	0.13695	
12	SW8	SW	female	0.14486	0.14486	
8	SW15	SW	male	0.14591	0.14591	
17	SW17	SW	male	0.15146	0.15146	
9	SW2	SW	female	0.17198	0.17198	
19	SW19	SW	male	0.18499	0.18499	
13	SW10	SW	female	0.19413	0.19413	
11	SW6	SW	female	0.21805	0.21805	
15	SW14	SW	female	0.22946	0.22946	
3	SW5	SW	male	0.23403	0.23403	
5	SW9	SW	male	0.23976	0.23976	
6	SW11	SW	male	0.24798	0.24798	
19	SM19	SM	male	0.08787	0.08787	
3	SM5	SM	male	0.1086	0.1086	
5	SM9	SM	male	0.10961	0.10961	
4	SM7	SM	male	0.11548	0.11548	
1	SM1	SM	male	0.11751	0.11751	
6	SM11	SM	male	0.1329	0.1329	
13	SM10	SM	female	0.13308	0.13308	
17	SM17	SM	male	0.13874	0.13874	
12	SM8	SM	female	0.14185	0.14185	
18	SM18	SM	female	0.15033	0.15033	
14	SM12	SM	female	0.15384	0.15384	
10	SM4	SM	female	0.17835	0.17835	
20	SM20	SM	female	0.19209	0.19209	
2	SM3	SM	male	0.19918	0.19918	
8	SM15	SM	male	0.21641	0.21641	
7	SM13	SM	male	0.22131	0.22131	
15	SM14	SM	female	0.2359	0.2359	
16	SM16	SM	female	0.23719	0.23719	
11	SM6	SM	female	0.24446	0.24446	
9	SM2	SM	female	0.28982		

Fig 3F **PDE11 Camkii Correlation**

VAR1	VAR2	VAR3	VAR4	e11/actin	e11/actin	nA/camA	nB/camB
1	GH1	GH	male	1.3844	0.4513	1.2055	0.9023
13	GH10	GH	female		0.4911	2.3024	1.572
6	GH11	GH	male	0.1652	0.3577	1.5832	1.4139
14	GH12	GH	female	1.0595	0.4565	0.9408	2.7
7	GH13	GH	male	0.1834		0	0.8735
15	GH14	GH	female	0.7663	0.4537		
8	GH15	GH	male	1.8338	0.2988	3.1	2.167
16	GH16	GH	female	0.9249	0.2586	3.3706	1.3092
17	GH17	GH	male	0.7593	0.4827	0.6288	0.9586
18	GH18	GH	female	1.0547	0.6389	1.5592	1.022
19	GH19	GH	male	0.5194	0.5422	1.8978	1.3317
9	GH2	GH	female	1.2003	0.4751	2.8387	1.5072
20	GH20	GH	female	1.0664	0.3698	2.8807	1.675
2	GH3	GH	male	0.7622	0.2744	3.2989	
10	GH4	GH	female	0.5673	0.2952	0.325	0.8457
3	GH5	GH	male	0.3097	0.6172	0.6815	2.26
11	GH6	GH	female	0.3655	0.4765	1.7359	1.5652
4	GH7	GH	male	0.6486	0.2083	1.5959	1.5932
12	GH8	GH	female	1.0059	0.4151	0.0732	1.028
5	GH9	GH	male	1.9337	0.5205	0.9081	0.7429
1	SD1	SD	male	0.737	0.5426	0.5882	0.9343
13	SD10	SD	female	0.3691	0.5812	0.1024	0.9707
6	SD11	SD	male	0.6236	0.5422	0.4516	1.5834
14	SD12	SD	female	0.2435	0.4176	0.2889	0.9924
7	SD13	SD	male	0.6781	0.4455	0.5821	0.9494
15	SD14	SD	female	0.8538	0.3904	0.4176	0.4837
8	SD15	SD	male	0.7405	0.2571	0.7051	0.6411
16	SD16	SD	female	0.6152	0.6735	0.058	1.7922
17	SD17	SD	male	0.7655	0.5522		1.1902
18	SD18	SD	female	0.5133	0.4532	0.5033	1.7654
19	SD19	SD	male	1.0466	0.4869	0.8738	2.2849
9	SD2	SD	female	0.5846	0.5211	0.5198	0.7586
20	SD20	SD	female	0.4835	0.3887	0.6242	1.5663
2	SD3	SD	male	0.4659	0.3751	0.4744	1.9181
10	SD4	SD	female	0.2568	0.6497	0.2188	
3	SD5	SD	male		0.48	0.4823	0.7296
11	SD6	SD	female	0.2821	0.6035	0.00664	1.0599
4	SD7	SD	male	0.7109	0.3451	0.5448	0.6172
12	SD8	SD	female	0.2379		0.1599	2.0913
5	SD9	SD	male	1.1938	0.5312	0.7284	1.1293
1	SH1	SH	male	0.3836	0.5354	0.5216	1.1108
13	SH10	SH	female	0.1539	0.4714	0.0571	1.2833
6	SH11	SH	male	0.3808	0.3889	0.1682	0.8763
14	SH12	SH	female	0.3371	0.3606		
7	SH13	SH	male	0.4313	0.5192	0.0841	1.0978
15	SH14	SH	female	0.3743	0.577	0.1363	2.6185
16	SH16	SH	female	0.6311	0.6013	0.0418	1.2496
17	SH17	SH	male	0.3471	0.2111	0.9875	0.9694
18	SH18	SH	female	0.5548	0.4683	0.1767	1.2192
19	SH19	SH	male	0.9102	0.3567	1.1702	1.2288
9	SH2	SH	female	0.5661	0.4351	0.4829	0.6536
20	SH20	SH	female	0.281	0.4114	0.8857	1.4181
2	SH3	SH	male	0.4261	0.5305	0.6583	2.0706
10	SH4	SH	female	0.4993	0.3834	1.5776	
3	SH5	SH	male	0.452	0.3446		3.1515
11	SH6	SH	female	0.3849	0.4053	1.8046	2.18
4	SH7	SH	male		0.4333	0.3833	0.5686
12	SH8	SH	female		0.5074	0.5088	2.0363
5	SH9	SH	male	0.3635	0.5894	0.0325	0.6861
1	SM1	SM	male	1.1709	0.5313	0.8943	0.9603
13	SM10	SM	female	0.7652	0.5802	0.1807	1.4758
6	SM11	SM	male	0.1525	0.3814	1.2777	2.0489
14	SM12	SM	female	0.3506	0.592	0.03	1.0024
7	SM13	SM	male	0.1336		0.057	1.2671
15	SM14	SM	female	0.6638	0.3786	0.2669	0.4972
8	SM15	SM	male	0.9501	0.1742	1.9979	
16	SM16	SM	female	0.6687	0.5234	0.172	1.2399
17	SM17	SM	male	0.3439	0.5768	0.6479	1.6635
18	SM18	SM	female	0.7679	0.6163	0.5593	2.1965
19	SM19	SM	male	0.5772	0.4776	0.3951	2.4227
9	SM2	SM	female		0.4835		2.8646
20	SM20	SM	female	0.9859	0.3737	1.3901	1.3552
2	SM3	SM	male	0.4815	0.1946	1.2959	1.8765
10	SM4	SM	female	0.2356	0.5725	0.013	0.5405
3	SM5	SM	male	0.1224	0.7495	0.0977	2.9106
11	SM6	SM	female	0.7349	0.479	1.6475	1.7896
4	SM7	SM	male	0.9386	0.3559	2.0754	1.4835
12	SM8	SM	female	0.5273	0.4114	0.8817	1.4349
5	SM9	SM	male	1.0565	0.5311	1.1776	0.7898
1	SW1	SW	male	0.4213		0.4058	0.8355
13	SW10	SW	female	0.837	0.4441	2.6673	1.5783
6	SW11	SW	male	0.3567	0.5592	2.1982	
14	SW12	SW	female	0.5142	0.4463	2.247	2.5833
7	SW13	SW	male	0.184	0.7934	0.1442	1.0902
15	SW14	SW	female	0.4209	0.5466	0.0199	0.7064
8	SW15	SW	male	0.8916	0.0739	2.1462	2.9205
16	SW16	SW	female	0.777	0.438	1.0256	1.7951
17	SW17	SW	male	0.8152	0.7843	0.4953	1.6537
18	SW18	SW	female	0.3885	0.8284	0.5456	2.3661
19	SW19	SW	male	0.6114	0.3707	1.5744	2.0279
9	SW2	SW	female	1.0063	0.4201	0.7073	0.9202
20	SW20	SW	female	0.5999	0.2922	0.6713	1.1476
2	SW3	SW	male	0.4631	0.3593	0.4533	1.6488
10	SW4	SW	female	0.3303	0.6054	0.0709	1.3031
3	SW5	SW	male	0.1486		0.0279	1.4289
11	SW6	SW	female		0.3756	0.829	1.3084
4	SW7	SW	male	0.7606	0.2805	1.1807	0.9883
12	SW8	SW	female	0.5122	0.4104	2.1489	1.2376
5	SW9	SW	male	0.4696	0.4395	1.2706	3.0367

Fig3G PDE11/S6 Correlation

VAR1	VAR2	VAR3	VAR4	e11/actin	e11/actin	6 no outs
1	GH1	GH	male	1.3844	0.4513	1.91843
13	GH10	GH	female		0.4911	0.70424
6	GH11	GH	male	0.1652	0.3577	1.34409
14	GH12	GH	female	1.0595	0.4565	1.1165
7	GH13	GH	male	0.1834		0.63688
15	GH14	GH	female	0.7663	0.4537	2.90412
8	GH15	GH	male	1.8338	0.2988	2.24226
16	GH16	GH	female	0.9249	0.2586	2.46666
17	GH17	GH	male	0.7593	0.4827	1.70684
18	GH18	GH	female	1.0547	0.6389	2.31935
19	GH19	GH	male	0.5194	0.5422	1.37618
9	GH2	GH	female	1.2003	0.4751	1.27672
20	GH20	GH	female	1.0664	0.3698	2.04986
2	GH3	GH	male	0.7622	0.2744	1.25429
10	GH4	GH	female	0.5673	0.2952	2.79905
3	GH5	GH	male	0.3097	0.6172	2.29834
11	GH6	GH	female	0.3655	0.4765	
4	GH7	GH	male	0.6486	0.2083	1.17444
12	GH8	GH	female	1.0059	0.4151	0.84907
5	GH9	GH	male	1.9337	0.5205	0.80785
1	SD1	SD	male	0.737	0.5426	
13	SD10	SD	female	0.3691	0.5812	0.5821
6	SD11	SD	male	0.6236	0.5422	1.63523
14	SD12	SD	female	0.2435	0.4176	2.51068
7	SD13	SD	male	0.6781	0.4455	1.85965
15	SD14	SD	female	0.8538	0.3904	2.08652
8	SD15	SD	male	0.7405	0.2571	1.73653
16	SD16	SD	female	0.6152	0.6735	0.5127
17	SD17	SD	male	0.7655	0.5522	1.80733
18	SD18	SD	female	0.5133	0.4532	0.94972
19	SD19	SD	male	1.0466	0.4869	1.3559
9	SD2	SD	female	0.5846	0.5211	1.55823
20	SD20	SD	female	0.4835	0.3887	1.32258
2	SD3	SD	male	0.4659	0.3751	0.80555
10	SD4	SD	female	0.2568	0.6497	0.84022
3	SD5	SD	male		0.48	1.49512
11	SD6	SD	female	0.2821	0.6035	0.58969
4	SD7	SD	male	0.7109	0.3451	1.21329
12	SD8	SD	female	0.2379		0.51614
5	SD9	SD	male	1.1938	0.5312	0.98119
1	SH1	SH	male	0.3836	0.5354	0.7431
13	SH10	SH	female	0.1539	0.4714	0.89267
6	SH11	SH	male	0.3808	0.3889	1.89818
14	SH12	SH	female	0.3371	0.3606	
7	SH13	SH	male	0.4313	0.5192	1.43846
15	SH14	SH	female	0.3743	0.577	0.77593
16	SH16	SH	female	0.6311	0.6013	0.51676
17	SH17	SH	male	0.3471	0.2111	1.62745
18	SH18	SH	female	0.5548	0.4683	0.78697
19	SH19	SH	male	0.9102	0.3567	1.79834
9	SH2	SH	female	0.5661	0.4351	1.00509
20	SH20	SH	female	0.281	0.4114	1.21821
2	SH3	SH	male	0.4261	0.5305	0.97625
10	SH4	SH	female	0.4993	0.3834	2.03553
3	SH5	SH	male	0.452	0.3446	1.88812
11	SH6	SH	female	0.3849	0.4053	1.50367
4	SH7	SH	male		0.4333	1.23629
12	SH8	SH	female		0.5074	0.62429
5	SH9	SH	male	0.3635	0.5894	0.64188
1	SM1	SM	male	1.1709	0.5313	1.37237
13	SM10	SM	female	0.7652	0.5802	0.64081
6	SM11	SM	male	0.1525	0.3814	1.72133
14	SM12	SM	female	0.3506	0.592	1.57731
7	SM13	SM	male	0.1336		0.98545
15	SM14	SM	female	0.6638	0.3786	1.50593
8	SM15	SM	male	0.9501	0.1742	1.37889
16	SM16	SM	female	0.6687	0.5234	0.87516
17	SM17	SM	male	0.3439	0.5768	1.02597
18	SM18	SM	female	0.7679	0.6163	1.18463
19	SM19	SM	male	0.5772	0.4776	0.54114
9	SM2	SM	female		0.4835	
20	SM20	SM	female	0.9859	0.3737	1.41905
2	SM3	SM	male	0.4815	0.1946	0.79649
10	SM4	SM	female	0.2356	0.5725	0.25481
3	SM5	SM	male	0.1224	0.7495	0.63558
11	SM6	SM	female	0.7349	0.479	1.46078
4	SM7	SM	male	0.9386	0.3559	1.06312
12	SM8	SM	female	0.5273	0.4114	1.11076
5	SM9	SM	male	1.0565	0.5311	1.29038
1	SW1	SW	male	0.4213		2.03971
13	SW10	SW	female	0.837	0.4441	
6	SW11	SW	male	0.3567	0.5592	
14	SW12	SW	female	0.5142	0.4463	1.84239
7	SW13	SW	male	0.184	0.7934	1.29806
15	SW14	SW	female	0.4209	0.5466	0.9772
8	SW15	SW	male	0.8916	0.0739	0.97209
16	SW16	SW	female	0.777	0.438	1.32155
17	SW17	SW	male	0.8152	0.7843	1.57965
18	SW18	SW	female	0.3885	0.8284	1.01591
19	SW19	SW	male	0.6114	0.3707	1.12373
9	SW2	SW	female	1.0063	0.4201	1.48963
20	SW20	SW	female	0.5999	0.2922	1.67626
2	SW3	SW	male	0.4631	0.3593	0.78444
10	SW4	SW	female	0.3303	0.6054	0.42618
3	SW5	SW	male	0.1486		0.94478
11	SW6	SW	female		0.3756	1.29783
4	SW7	SW	male	0.7606	0.2805	1.99345
12	SW8	SW	female	0.5122	0.4104	1.48972
5	SW9	SW	male	0.4696	0.4395	0.78302

Fig 3H SOR

mouse	SEX	Condition	24h total	7d total	24hr ratio	7dratio	24hr ratio no out	7dratio no outs
1109	FEMALE	GH	39	21	0.666666667	-0.3	0.666666667	-0.3
1107	FEMALE	GH	34	23	0.225806452	-0.238095238	0.225806452	-0.238095238
1894	FEMALE	GH	37	18	0.454545455	-0.176470588	0.454545455	-0.176470588
1901	FEMALE	GH	26	11	0.52	0	0.52	0
945	MALE	GH	22	26	0.727272727	0	0.727272727	0
946	FEMALE	GH	44	31	0.590909091	0.066666667	0.590909091	0.066666667
1170	MALE	GH	34	28	0.6875	0.076923077	0.6875	0.076923077
1896	MALE	GH	8	9	0.428571429	0.111111111	0.428571429	0.111111111
1897	MALE	GH	26	9	-0.142857143	0.142857143	-0.142857143	0.142857143
1173	MALE	GH	27	39	0.333333333	0.179487179	0.333333333	0.179487179
1895	FEMALE	GH	61	38	-0.5	0.2		0.2
1174	MALE	GH	26	15	0.826086957	0.2	0.826086957	0.2
944	FEMALE	GH	31	36	0.35483871	0.257142857	0.35483871	0.257142857
1106	FEMALE	GH	29	29	0.5	0.285714286	0.5	0.285714286
943	MALE	GH	27	39	0.851851852	0.351351351	0.851851852	0.351351351
1110	FEMALE	GH	20	23	0.375	0.363636364	0.375	0.363636364
1108	FEMALE	GH	27	33	0.28	0.4375	0.28	0.4375
1905	MALE	GH	16	11	0.066666667	0.454545455	0.066666667	0.454545455
1904	MALE	GH	25	27	0.75	0.769230769	0.75	0.769230769
1898	FEMALE	SH	37	28	-0.454545455	-0.44		
1968	FEMALE	SH	19	16	0.444444444	0.142857143	0.444444444	0.142857143
1909	MALE	SH	43	18	0.487179487	0.222222222	0.487179487	0.222222222
941	MALE	SH	49	43	0.458333333	0.238095238	0.458333333	0.238095238
1899	FEMALE	SH	34	46	0.032258065	0.244444444	0.032258065	0.244444444
924	FEMALE	SH	35	32	0.454545455	0.25	0.454545455	0.25
1115	FEMALE	SH	37	39	0.777777778	0.315789474	0.777777778	0.315789474
1907	MALE	SH	48	41	0.288888889	0.35	0.288888889	0.35
1908	MALE	SH	28	35	0.5	0.352941176	0.5	0.352941176
1112	FEMALE	SH	50	44	0.375	0.476190476	0.375	0.476190476
1114	MALE	SH	40	40	0.230769231	0.487179487	0.230769231	0.487179487
1906	MALE	SH	54	30	0.346153846	0.5	0.346153846	0.5
942	FEMALE	SH	49	54	0.510204082	0.538461538	0.510204082	0.538461538
1900	MALE	SH	42	49	0.384615385	0.551020408	0.384615385	0.551020408
1176	MALE	SH	36	32	0.352941176	0.625	0.352941176	0.625
1111	FEMALE	SH	33	30	0.333333333	0.714285714	0.333333333	0.714285714
925	MALE	SH	36	41	0.666666667	0.722222222	0.666666667	0.722222222
1113	FEMALE	SH	67	38	0.8125	0.722222222	0.8125	0.722222222
1171	MALE	SH	30	33	0.379310345	0.75	0.379310345	0.75
1105	MALE	SH	62	47	0.737704918	0.826086957	0.737704918	0.826086957
1116	FEMALE	HT	37	34	0.166666667	-0.161290323	0.166666667	-0.161290323
1124	MALE	HT	17	39	0.058823529	-0.111111111	0.058823529	-0.111111111
1025	FEMALE	HT	44	30	0.142857143	-0.066666667	0.142857143	-0.066666667
1151	FEMALE	HT	38	32	0.166666667	0.133333333	0.166666667	0.133333333
1022	FEMALE	HT	50	40	0.361702128	0.45	0.361702128	0.45
1153	MALE	HT	31	52	0.24137931	0.461538462	0.24137931	0.461538462
1117	FEMALE	HT	30	21	0.466666667	0.6	0.466666667	0.6
1120	FEMALE	HT	49	23	0.644444444	0.619047619	0.644444444	0.619047619
1118	MALE	HT	59	38	0.5	0.631578947	0.5	0.631578947
1152	FEMALE	HT	30	41	0.517241379	0.853658537	0.517241379	0.853658537

Fig 3I NSOR

mouse	SEX	Condition	24hr ratio	7dratio	24hr ratio	7dratio no outs
1905	MALE	GH	-0.2	0.333333		0.333333
946	FEMALE	GH	0	0.25	0	0.25
1109	FEMALE	GH	0.14285714	0.888889	0.142857	0.888889
1107	FEMALE	GH	0.18518519	0.125	0.185185	0.125
1894	FEMALE	GH	0.2	0	0.2	0
1904	MALE	GH	0.2	0.142857	0.2	0.142857
943	MALE	GH	0.25	0.444444	0.25	0.444444
1895	FEMALE	GH	0.25	0.555556	0.25	0.555556
1901	FEMALE	GH	0.33333333	0.333333	0.333333	0.333333
1173	MALE	GH	0.375	0.909091	0.375	0.909091
944	FEMALE	GH	0.3877551	0.647059	0.387755	0.647059
1174	MALE	GH	0.42857143	0.428571	0.428571	0.428571
1896	MALE	GH	0.45454545	0.454545	0.454545	0.454545
1106	FEMALE	GH	0.47826087	0.333333	0.478261	0.333333
945	MALE	GH	0.5	1	0.5	1
1897	MALE	GH	0.6	0.142857	0.6	0.142857
1110	FEMALE	GH	0.7	0.818182	0.7	0.818182
1170	MALE	GH	0.75	-0.33333	0.75	
1108	FEMALE	GH	0.92857143	0.578947	0.928571	0.578947
1114	MALE	SH	0.11111111	1		1
1908	MALE	SH	0.16666667	0.043478	0.166667	0.043478
1111	FEMALE	SH	0.28571429	0.5	0.285714	0.5
1899	FEMALE	SH	0.29411765	0.636364	0.294118	0.636364
1900	MALE	SH	0.42857143	0.6	0.428571	0.6
1115	FEMALE	SH	0.43396226	-0.37931	0.433962	
1907	MALE	SH	0.5	0.8	0.5	0.8
1112	FEMALE	SH	0.52380952	0.6	0.52381	0.6
942	FEMALE	SH	0.53846154	-0.37931	0.538462	-0.37931
1176	MALE	SH	0.55555556	0.727273	0.555556	0.727273
1898	FEMALE	SH	0.58823529	0.8	0.588235	0.8
1105	MALE	SH	0.6	0.793103	0.6	0.793103
1171	MALE	SH	0.6	0.368421	0.6	0.368421
1906	MALE	SH	0.6	0.375	0.6	0.375
1968	FEMALE	SH	0.61538462	-0.33333	0.615385	-0.33333
924	FEMALE	SH	0.64705882	0.636364	0.647059	0.636364
1909	MALE	SH	0.67741935	0.866667	0.677419	0.866667
1113	FEMALE	SH	0.7037037	0.411765	0.703704	0.411765
925	MALE	SH	0.77272727	0.176471	0.772727	0.176471
941	MALE	SH	0.78947368	0.846154	0.789474	0.846154

Fig4A **VHM IL6**

squads	id	group	sex	cd actin	cdpde11	cd il6	VAR8	VAR9
1	GH1	GH	male	0.07451	1.42987		2.65174	
1	SM1	SM	male	0.07096	1.20943	1.15657		
1	SW1	SW	male	0.38081	0.43518	1.7294		
1	SD1	SD	male	0.09816	0.76123	2.26049		
1	SH1	SH	male	0.46123	0.39619	1.62117		
2	GH3	GH	male	0.17385	0.78728	0.81009		
2	SM3	SM	male	0.42102	0.49729	0.8519		
2	SW3	SW	male	0.56767	0.4783	0.46405		
2	SD3	SD	male	0.53811	0.48121	0.62324		
2	SH3	SH	male	0.56649	0.44012	0.46145		
3	GH5	GH	male	0.30886	0.3199	1.46355		
3	SM5	SM	male	0.60322	0.12641	2.01133		
3	SW5	SW	male	0.73479	0.15347	1.74759		
3	SD5	SD	male	0.0669		0.94123	1.41268	
3	SH5	SH	male	0.25534	0.46687	1.56334		
4	GH7	GH	male	0.29659	0.66989	0.82269		
4	SM7	SM	male	0.1706	0.96948	1.25234		
4	SW7	SW	male	0.15722	0.78558	1.23733		
4	SD7	SD	male	0.54412	0.73423	0.44637		
4	SH7	SH	male	0.2152		1.14432	1.2776	
5	GH9	GH	male	0.09931	1.99731	1.38116		
5	SM9	SM	male	0.12378	1.09127	0.8912		
5	SW9	SW	male	0.37134	0.48501	0.9811		
5	SD9	SD	male	0.20726	1.233	1.01196		
5	SH9	SH	male	0.46993	0.37548	1.76211		
6	GH11	GH	male	0.32816	0.17058	0.36048		
6	SM11	SM	male	0.43251	0.15756	0.84234		
6	SW11	SW	male	0.3699	0.36846	1.00454		
6	SD11	SD	male	0.47425	0.64406	0.91052		
6	SH11	SH	male	0.4764	0.39337	0.79519		
7	GH13	GH	male	0.50092	0.18938		2.61978	
7	SM13	SM	male	0.42108	0.13799	2.40615		
7	SW13	SW	male	0.44295	0.19007	1.94727		
7	SD13	SD	male	0.31827	0.70044	0.93537		
7	SH13	SH	male	0.50311	0.44546	1.03093		
8	GH15	GH	male	0.09078	1.89407	1.17402		
8	SM15	SM	male	0.27671	0.98135	0.72666		
8	SW15	SW	male	0.18156	0.92091	0.19958		
8	SD15	SD	male	0.28218	0.76482	0.32733		
9	GH2	GH	female	0.10571	1.23977	1.42757		
9	SM2	SM	female	0.05857		2.3784	1.49701	
9	SW2	SW	female	0.09857	1.03934	1.18841		
9	SD2	SD	female	0.15286	0.60381	0.86992		
9	SH2	SH	female	0.22571	0.58474	1.66451		
10	GH4	GH	female	0.49142	0.58598	1.10382		
10	SM4	SM	female	0.62571	0.24339	1.0086		
10	SW4	SW	female	0.48428	0.34114	0.98935		
10	SD4	SD	female	0.48714	0.26525	1.27601		
10	SH4	SH	female	0.62285	0.51568	0.72349		
11	GH6	GH	female	0.55139	0.3775	1.60977		
11	SM6	SM	female	0.38242	0.75903	1.05925		
11	SW6	SW	female	0.16799		0.8262	1.64834	
11	SD6	SD	female	0.57017	0.29139	2.48517		
11	SH6	SH	female	0.41305	0.3976	1.37141		
12	GH8	GH	female	0.13142	1.03892	1.8051		
12	SM8	SM	female	0.21739	0.54463	0.36376		
12	SW8	SW	female	0.34289	0.52908	1.12488		
12	SD8	SD	female	0.46246	0.2457	1.23187		
12	SH8	SH	female	0.11364	1.0839	0.75269		
13	GH10	GH	female	0.1283	2.1339	1.07178		
13	SM10	SM	female	0.36986	0.79033	2.40331		
13	SW10	SW	female	0.33979	0.86447	1.01653		
13	SD10	SD	female	0.5162	0.3812	2.10888		
13	SH10	SH	female	0.52021	0.15898		3.23491	
14	GH12	GH	female	0.05212	1.09431	1.16208		
14	SM12	SM	female	0.27564	0.36212	1.27686		
14	SW12	SW	female	0.22553	0.5311	1.00895		
14	SD12	SD	female	0.39693	0.25147	0.86196		
14	SH12	SH	female	0.52823	0.34823	0.70038		
15	GH14	GH	female	0.23954	0.79152	0.82317		
15	SM14	SM	female	0.27652	0.68567	0.73049		
15	SW14	SW	female	0.33118	0.43476	1.67584		
15	SD14	SD	female	0.2926	0.88186	0.98293		
15	SH14	SH	female	0.40192	0.38662	2.26878		
16	GH16	GH	female	0.33279	0.95528	0.70236		
16	SM16	SM	female	0.41076	0.69065	0.7634		
16	SW16	SW	female	0.32154	0.8025	0.83165		
16	SD16	SD	female	0.35449	0.63539	1.93738		
16	SH16	SH	female	0.39147	0.65185	1.91409		

squads	id	group	sex	cd actin	cdpde11	cd il6	VAR17	VAR18
1	GH1	GH	male	0.0745	1.4299		2.6517	
2	GH3	GH	male	0.1738	0.7873	0.8101		
3	GH5	GH	male	0.3089	0.3199	1.4635		
4	GH7	GH	male	0.2966	0.6699	0.8227		
5	GH9	GH	male	0.0993	1.9973	1.3812		
6	GH11	GH	male	0.3282	0.1706	0.3605		
7	GH13	GH	male	0.5009	0.1894		2.6198	
8	GH15	GH	male	0.0908	1.8941	1.174		
9	GH2	GH	female	0.1057	1.2398	1.4276		
10	GH4	GH	female	0.4914	0.586	1.1038		
11	GH6	GH	female	0.5514	0.3775	1.6098		
12	GH8	GH	female	0.1314	1.0389	1.8051		
13	GH10	GH	female	0.1283	2.1339	1.0718		
14	GH12	GH	female	0.0521	1.0943	1.1621		
15	GH14	GH	female	0.2395	0.7915	0.8232		
16	GH16	GH	female	0.3328	0.9553	0.7024		
1	SD1	SD	male	0.0982	0.7612	2.2605		
2	SD3	SD	male	0.5381	0.4812	0.6232		
3	SD5	SD	male	0.0669		0.9412	1.4127	
4	SD7	SD	male	0.5441	0.7342	0.4464		
5	SD9	SD	male	0.2073	1.233	1.012		
6	SD11	SD	male	0.4742	0.6441	0.9105		
7	SD13	SD	male	0.3183	0.7004	0.9354		
8	SD15	SD	male	0.2822	0.7648	0.3273		
9	SD2	SD	female	0.1529	0.6038	0.8699		
10	SD4	SD	female	0.4871	0.2653	1.276		
11	SD6	SD	female	0.5702	0.2914	2.4852		
12	SD8	SD	female	0.4625	0.2457	1.2319		
13	SD10	SD	female	0.5162	0.3812	2.1089		
14	SD12	SD	female	0.3969	0.2515	0.862		
15	SD14	SD	female	0.2926	0.8819	0.9829		
16	SD16	SD	female	0.3545	0.6354	1.9374		
1	SW1	SW	male	0.3808	0.4352	1.7294		
2	SW3	SW	male	0.5677	0.4783	0.4641		
3	SW5	SW	male	0.7348	0.1535	1.7476		
4	SW7	SW	male	0.1572	0.7856	1.2373		
5	SW9	SW	male	0.3713	0.485	0.9811		
6	SW11	SW	male	0.3699	0.3685	1.0045		
7	SW13	SW	male	0.443	0.1901	1.9473		
8	SW15	SW	male	0.1816	0.9209	0.1996		
9	SW2	SW	female	0.0986	1.0393	1.1884		
10	SW4	SW	female	0.4843	0.3411	0.9894		
11	SW6	SW	female	0.168		0.8262	1.6483	
12	SW8	SW	female	0.3429	0.5291	1.1249		
13	SW10	SW	female	0.3398	0.8645	1.0165		
14	SW12	SW	female	0.2255	0.5311	1.0089		
15	SW14	SW	female	0.3312	0.4348	1.6758		
16	SW16	SW	female	0.3215	0.8025	0.8316		
1	SM1	SM	male	0.071	1.2094	1.1566		
2	SM3	SM	male	0.421	0.4973	0.8519		
3	SM5	SM	male	0.6032	0.1264	2.0113		
4	SM7	SM	male	0.1706	0.9695	1.2523		
5	SM9	SM	male	0.1238	1.0913	0.8912		
6	SM11	SM	male	0.4325	0.1576	0.8423		
7	SM13	SM	male	0.4211	0.138	2.4061		
8	SM15	SM	male	0.2767	0.9813	0.7267	1.497	
9	SM2	SM	female	0.0586		2.3784		
10	SM4	SM	female	0.6257	0.2434	1.0086		
11	SM6	SM	female	0.3824	0.759	1.0593		
12	SM8	SM	female	0.2174	0.5446	0.3638		
13	SM10	SM	female	0.3699	0.7903	2.4033		
14	SM12	SM	female	0.2756	0.3621	1.2769		
15	SM14	SM	female	0.2765	0.6857	0.7305		
16	SM16	SM	female	0.4108	0.6906	0.7634		

squads	id	group	sex	cd actin	cdpde11	cd il6	VAR26
1	GH1	GH	male	0.0745	1.4299		2.6517
2	GH3	GH	male	0.1738	0.7873	0.8101	
3	GH5	GH	male	0.3089	0.3199	1.4635	
4	GH7	GH	male	0.2966	0.6699	0.8227	
5	GH9	GH	male	0.0993	1.9973	1.3812	
6	GH11	GH	male	0.3282	0.1706	0.3605	
7	GH13	GH	male	0.5009	0.1894		2.6198
8	GH15	GH	male	0.0908	1.8941	1.174	
9	GH2	GH	female	0.1057	1.2398	1.4276	
10	GH4	GH	female	0.4914	0.586	1.1038	
11	GH6	GH	female	0.5514	0.3775	1.6098	
12	GH8	GH	female	0.1314	1.0389	1.8051	
13	GH10	GH	female	0.1283	2.1339	1.0718	
14	GH12	GH	female	0.0521	1.0943	1.1621	
15	GH14	GH	female	0.2395	0.7915	0.8232	
16	GH16	GH	female	0.3328	0.9553	0.7024	
1	SM1	SM	male	0.071	1.2094	1.1566	
2	SM3	SM	male	0.421	0.4973	0.8519	
3	SM5	SM	male	0.6032	0.1264	2.0113	
4	SM7	SM	male	0.1706	0.9695	1.2523	
5	SM9	SM	male	0.1238	1.0913	0.8912	
6	SM11	SM	male	0.4325	0.1576	0.8423	
7	SM13	SM	male	0.4211	0.138	2.4061	
8	SM15	SM	male	0.2767	0.9813	0.7267	1.497
9	SM2	SM	female	0.0586		2.3784	
10	SM4	SM	female	0.6257	0.2434	1.0086	
11	SM6	SM	female	0.3824	0.759	1.0593	
12	SM8	SM	female	0.2174	0.5446	0.3638	
13	SM10	SM	female	0.3699	0.7903	2.4033	
14	SM12	SM	female	0.2756	0.3621	1.2769	
15	SM14	SM	female	0.2765	0.6857	0.7305	
16	SM16	SM	female	0.4108	0.6906	0.7634	

Fig4B **VHC IL6**

squads	id	group	sex	ale pde11 I	male il6	VAR7
7	GH13	GH	males			
6	GH11	GH	males	0.4046	0.7552	
5	GH9	GH	males	0.5888	0.7189	
2	GH3	GH	males	0.3104	0.4796	
3	GH5	GH	males	0.6981	1.6816	
1	GH1	GH	males	0.5105	1.4891	
4	GH7	GH	males	0.2356	0.7282	
8	GH15	GH	males	0.338	0.5517	
7	SM13	SM	males	1.0395	2.1896	
3	SM5	SM	males	0.8478	2.3931	
1	SM1	SM	males	0.601	0.6948	
6	SM11	SM	males	0.4314	1.9407	
2	SM3	SM	males	0.2202	1.6993	
4	SM7	SM	males	0.4026	1.5928	
5	SM9	SM	males	0.6008	0.6682	
8	SM15	SM	males	0.1971	1.5966	
3	SW5	SW	males	1.1616	2.8483	
1	SW1	SW	males	1.1838	3.5073	
7	SW13	SW	males	0.8975	1.1811	
6	SW11	SW	males	0.6325	1.2671	
5	SW9	SW	males	0.4972	1.2547	
4	SW7	SW	males	0.3173	1.2294	
8	SW15	SW	males	0.0835	0.4436	
2	SW3	SW	males	0.4064	0.7413	
1	SD1	SD	males	0.6138	0.8543	
3	SD5	SD	males	0.5429	0.4162	
7	SD13	SD	males	0.5039	0.2771	
6	SD11	SD	males	0.6133	1.3579	
5	SD9	SD	males	0.6009	1.2579	
4	SD7	SD	males	0.3903	0.5831	
2	SD3	SD	males	0.4243	1.1942	
8	SD15	SD	males	0.2909	0.4373	
5	SH9	SH	males			
4	SH7	SH	males	0.4901	0.9112	
1	SH1	SH	males	0.6056	1.149	
7	SH13	SH	males	0.5873	0.2774	
2	SH3	SH	males	0.6001	0.7649	
6	SH11	SH	males	0.4399	1.2715	
3	SH5	SH	males	0.3898	0.19	

squad	id	group	sex	:s per sex	VAR13	VAR14
13	GH10	GH	females	0.5193		
15	GH14	GH	females	0.7677		
14	GH12	GH	females	1.0562		
12	GH8	GH	females	1.1212		
10	GH4	GH	females	1.1528		
16	GH16	GH	females	1.1927		
9	GH2	GH	females	1.2145		
11	GH6	GH	females	1.5211		
15	SM14	SM	females	0.6235		
11	SM6	SM	females	0.6299		
13	SM10	SM	females	0.8392		
12	SM8	SM	females	0.9004		
10	SM4	SM	females	0.9646		
16	SM16	SM	females	1.1124		
9	SM2	SM	females	1.2946		
14	SM12	SM	females	1.4379		
11	SW6	SW	females	0.574		
9	SW2	SW	females	0.6482		
15	SW14	SW	females	0.761		
13	SW10	SW	females	0.8119		
10	SW4	SW	females	1.1709		
12	SW8	SW	females	1.4059		
16	SW16	SW	females	1.7009		
14	SW12	SW	females		2.9459	
9	SD2	SD	females	0.5116		
11	SD6	SD	females	0.6371		
15	SD14	SD	females	1.0046		
13	SD10	SD	females	1.1023		
14	SD12	SD	females	1.804		
16	SD16	SD	females	1.8043		
10	SD4	SD	females	2.5369		
12	SD8	SD	females	2.5956		
9	SH2	SH	females		1.2979	
11	SH6	SH	females	1.5624		
12	SH8	SH	females	1.6263		
10	SH4	SH	females	1.7818		
16	SH16	SH	females	1.8008		
15	SH14	SH	females	1.8062		
14	SH12	SH	females	1.8768		
13	SH10	SH	females	1.943		
2	GH3	GH	males	0.4796		
8	GH15	GH	males	0.5517		
5	GH9	GH	males	0.7189		
4	GH7	GH	males	0.7282		
6	GH11	GH	males	0.7552		
1	GH1	GH	males	1.4891		
3	GH5	GH	males	1.6816		
7	GH13	GH	males			
5	SM9	SM	males	0.6682		
1	SM1	SM	males	0.6948		
4	SM7	SM	males	1.5928		
8	SM15	SM	males	1.5966		
2	SM3	SM	males	1.6993		
6	SM11	SM	males	1.9407		
7	SM13	SM	males	2.1896		
3	SM5	SM	males	2.3931		
8	SW15	SW	males	0.4436		
2	SW3	SW	males	0.7413		
7	SW13	SW	males	1.1811		
4	SW7	SW	males	1.2294		
5	SW9	SW	males	1.2547		
6	SW11	SW	males	1.2671		
3	SW5	SW	males	2.8483		
1	SW1	SW	males	3.5073		
7	SD13	SD	males	0.2771		
3	SD5	SD	males	0.4162		
8	SD15	SD	males	0.4373		
4	SD7	SD	males	0.5831		
1	SD1	SD	males	0.8543		
2	SD3	SD	males	1.1942		
5	SD9	SD	males	1.2579		
6	SD11	SD	males	1.3579		
3	SH5	SH	males	0.19		
7	SH13	SH	males	0.2774		
2	SH3	SH	males	0.7649		
4	SH7	SH	males	0.9112		
1	SH1	SH	males	1.149		
6	SH11	SH	males	1.2715		
5	SH9	SH	males			

le squads	VAR16	female groups	VAR18	ale cd il6	VAR20
13	GH10	GH	females	0.5193	
15	GH14	GH	females	0.7677	
14	GH12	GH	females	1.0562	
12	GH8	GH	females	1.1212	
10	GH4	GH	females	1.1528	
16	GH16	GH	females	1.1927	
9	GH2	GH	females	1.2145	
11	GH6	GH	females	1.5211	
15	SM14	SM	females	0.6235	
11	SM6	SM	females	0.6299	
13	SM10	SM	females	0.8392	
12	SM8	SM	females	0.9004	
10	SM4	SM	females	0.9646	
16	SM16	SM	females	1.1124	
9	SM2	SM	females	1.2946	
14	SM12	SM	females	1.4379	
11	SW6	SW	females	0.574	
9	SW2	SW	females	0.6482	
15	SW14	SW	females	0.761	
13	SW10	SW	females	0.8119	
10	SW4	SW	females	1.1709	
12	SW8	SW	females	1.4059	
16	SW16	SW	females	1.7009	2.9459
14	SW12	SW	females		
9	SD2	SD	females	0.5116	
11	SD6	SD	females	0.6371	
15	SD14	SD	females	1.0046	
13	SD10	SD	females	1.1023	
14	SD12	SD	females	1.804	
16	SD16	SD	females	1.8043	
10	SD4	SD	females	2.5369	
12	SD8	SD	females	2.5956	1.2979
9	SH2	SH	females		
11	SH6	SH	females	1.5624	
12	SH8	SH	females	1.6263	
10	SH4	SH	females	1.7818	
16	SH16	SH	females	1.8008	
15	SH14	SH	females	1.8062	
14	SH12	SH	females	1.8768	
13	SH10	SH	females	1.943	

Fig 4C **PDE11 IL6 Correlation**

squad	id	group	sex	c il6 fem	de11 fem	de11 fem	VAR8
13	GH10	GH	females	0.5193	0.5556	2.1339	
14	GH12	GH	females	1.0562	0.5164	1.0943	
15	GH14	GH	females	0.7677	0.5132	0.7915	
16	GH16	GH	females	1.1927	0.2925	0.9553	
9	GH2	GH	females	1.2145	0.5374	1.2398	
10	GH4	GH	females	1.1528	0.3339	0.586	
11	GH6	GH	females	1.5211	0.539	0.3775	
12	GH8	GH	females	1.1212	0.4695	1.0389	
13	SD10	SD	females	1.1023	0.6575	0.3812	
14	SD12	SD	females	1.804	0.4723	0.2515	
15	SD14	SD	females	1.0046	0.4416	0.8819	
16	SD16	SD	females	1.8043	0.7618	0.6354	
9	SD2	SD	females	0.5116	0.5894	0.6038	
10	SD4	SD	females	2.5369	0.7349	0.2653	
11	SD6	SD	females	0.6371	0.6826	0.2914	
12	SD8	SD	females	2.5956	0.8561	0.2457	
13	SH10	SH	females	1.943	0.5333	0.159	
14	SH12	SH	females	1.8768	0.408	0.3482	
15	SH14	SH	females	1.8062	0.6527	0.3866	
16	SH16	SH	females	1.8008	0.6802	0.6519	
9	SH2	SH	females		0.4922	0.5847	
10	SH4	SH	females	1.7818	0.4337	0.5157	
11	SH6	SH	females	1.5624	0.4585	0.3976	
12	SH8	SH	females	1.6263	0.5739		
13	SM10	SM	females	0.8392	0.6563	0.7903	
14	SM12	SM	females	1.4379	0.6697	0.3621	
15	SM14	SM	females	0.6235	0.4282	0.6857	
16	SM16	SM	females	1.1124	0.5921	0.6906	
9	SM2	SM	females	1.2946	0.5469		
10	SM4	SM	females	0.9646	0.6477	0.2434	
11	SM6	SM	females	0.6299	0.5418	0.759	
12	SM8	SM	females	0.9004	0.4654	0.5446	
13	SW10	SW	females	0.8119	0.5023	0.8645	
14	SW12	SW	females		0.5048	0.5311	
15	SW14	SW	females	0.761	0.6183	0.4348	
16	SW16	SW	females	1.7009	0.4955	0.8025	
9	SW2	SW	females	0.6482	0.4752	1.0393	
10	SW4	SW	females	1.1709	0.6848	0.3411	
11	SW6	SW	females	0.574	0.4249		
12	SW8	SW	females	1.4059	0.4642	0.5291	

squad	id	group	sex	il6 male	e11 male	e11 male	VAR16
1	GH1	GH	males	1.4891	0.5105	1.4299	
6	GH11	GH	males	0.7552	0.4046	0.1706	
7	GH13	GH	males			0.1894	
8	GH15	GH	males	0.5517	0.338	1.8941	
2	GH3	GH	males	0.4796	0.3104	0.7873	
3	GH5	GH	males	1.6816	0.6981	0.3199	
4	GH7	GH	males	0.7282	0.2356	0.6699	
5	GH9	GH	males	0.7189	0.5888	1.9973	
1	SD1	SD	males	0.8543	0.6138	0.7612	
6	SD11	SD	males	1.3579	0.6133	0.6441	
7	SD13	SD	males	0.2771	0.5039	0.7004	
8	SD15	SD	males	0.4373	0.2909	0.7648	
2	SD3	SD	males	1.1942	0.4243	0.4812	
3	SD5	SD	males	0.4162	0.5429		
4	SD7	SD	males	0.5831	0.3903	0.7342	
5	SD9	SD	males	1.2579	0.6009	1.233	
1	SH1	SH	males	1.149	0.6056	0.3962	
6	SH11	SH	males	1.2715	0.4399	0.3934	
7	SH13	SH	males	0.2774	0.5873	0.4455	
2	SH3	SH	males	0.7649	0.6001	0.4401	
3	SH5	SH	males	0.19	0.3898	0.4669	
4	SH7	SH	males	0.9112	0.4901		
5	SH9	SH	males			0.3755	
1	SM1	SM	males	0.6948	0.601	1.2094	
6	SM11	SM	males	1.9407	0.4314	0.1576	
7	SM13	SM	males	2.1896		0.138	
8	SM15	SM	males	1.5966	0.1971	0.9813	
2	SM3	SM	males	1.6993	0.2202	0.4973	
3	SM5	SM	males	2.3931	0.8478	0.1264	
4	SM7	SM	males	1.5928	0.4026	0.9695	
5	SM9	SM	males	0.6682	0.6008	1.0913	
1	SW1	SW	males	3.5073		0.4352	
6	SW11	SW	males	1.2671	0.6325	0.3685	
7	SW13	SW	males	1.1811	0.8975	0.1901	
8	SW15	SW	males	0.4436	0.0835	0.9209	
2	SW3	SW	males	0.7413	0.4064	0.4783	
3	SW5	SW	males	2.8483			
4	SW7	SW	males	1.2294	0.3173	0.7856	
5	SW9	SW	males	1.2547	0.4972	0.485	

squad	id	group	sex	c il6 both	le11 both	le11 both
13	SD10	SD	females	1.1023	0.6575	0.3812
14	SD12	SD	females	1.804	0.4723	0.2515
15	SD14	SD	females	1.0046	0.4416	0.8819
16	SD16	SD	females	1.8043	0.7618	0.6354
9	SD2	SD	females	0.5116	0.5894	0.6038
10	SD4	SD	females	2.5369	0.7349	0.2653
11	SD6	SD	females	0.6371	0.6826	0.2914
12	SD8	SD	females	2.5956	0.8561	0.2457
1	SD1	SD	males	0.8543	0.6138	0.7612
6	SD11	SD	males	1.3579	0.6133	0.6441
7	SD13	SD	males	0.2771	0.5039	0.7004
8	SD15	SD	males	0.4373	0.2909	0.7648
2	SD3	SD	males	1.1942	0.4243	0.4812
3	SD5	SD	males	0.4162	0.5429	
4	SD7	SD	males	0.5831	0.3903	0.7342
5	SD9	SD	males	1.2579	0.6009	1.233
13	SH10	SH	females	1.943	0.5333	0.159
14	SH12	SH	females	1.8768	0.408	0.3482
15	SH14	SH	females	1.8062	0.6527	0.3866
16	SH16	SH	females	1.8008	0.6802	0.6519
9	SH2	SH	females		0.4922	0.5847
10	SH4	SH	females	1.7818	0.4337	0.5157
11	SH6	SH	females	1.5624	0.4585	0.3976
12	SH8	SH	females	1.6263	0.5739	
1	SH1	SH	males	1.149	0.6056	0.3962
6	SH11	SH	males	1.2715	0.4399	0.3934
7	SH13	SH	males	0.2774	0.5873	0.4455
2	SH3	SH	males	0.7649	0.6001	0.4401
3	SH5	SH	males	0.19	0.3898	0.4669
4	SH7	SH	males	0.9112	0.4901	
5	SH9	SH	males			0.3755
13	SM10	SM	females	0.8392	0.6563	0.7903
14	SM12	SM	females	1.4379	0.6697	0.3621
15	SM14	SM	females	0.6235	0.4282	0.6857
16	SM16	SM	females	1.1124	0.5921	0.6906
9	SM2	SM	females	1.2946	0.5469	
10	SM4	SM	females	0.9646	0.6477	0.2434
11	SM6	SM	females	0.6299	0.5418	0.759
12	SM8	SM	females	0.9004	0.4654	0.5446
1	SM1	SM	males	0.6948	0.601	1.2094
6	SM11	SM	males	1.9407	0.4314	0.1576
7	SM13	SM	males	2.1896		0.138
8	SM15	SM	males	1.5966	0.1971	0.9813
2	SM3	SM	males	1.6993	0.2202	0.4973
3	SM5	SM	males	2.3931	0.8478	0.1264
4	SM7	SM	males	1.5928	0.4026	0.9695
5	SM9	SM	males	0.6682	0.6008	1.0913
13	SW10	SW	females	0.8119	0.5023	0.8645
14	SW12	SW	females		0.5048	0.5311
15	SW14	SW	females	0.761	0.6183	0.4348
16	SW16	SW	females	1.7009	0.4955	0.8025
9	SW2	SW	females	0.6482	0.4752	1.0393
10	SW4	SW	females	1.1709	0.6848	0.3411
11	SW6	SW	females	0.574	0.4249	
12	SW8	SW	females	1.4059	0.4642	0.5291
1	SW1	SW	males	3.5073		0.4352
6	SW11	SW	males	1.2671	0.6325	0.3685
7	SW13	SW	males	1.1811	0.8975	0.1901
8	SW15	SW	males	0.4436	0.0835	0.9209
2	SW3	SW	males	0.7413	0.4064	0.4783
3	SW5	SW	males	2.8483		
4	SW7	SW	males	1.2294	0.3173	0.7856
5	SW9	SW	males	1.2547	0.4972	0.485

VAR24	VAR25	VAR26	VAR27	VAR28	VAR29	VAR30
13	GH10	GH	females	0.5193	0.5556	2.1339
14	GH12	GH	females	1.0562	0.5164	1.0943
15	GH14	GH	females	0.7677	0.5132	0.7915
16	GH16	GH	females	1.1927	0.2925	0.9553
9	GH2	GH	females	1.2145	0.5374	1.2398
10	GH4	GH	females	1.1528	0.3339	0.586
11	GH6	GH	females	1.5211	0.539	0.3775
12	GH8	GH	females	1.1212	0.4695	1.0389
1	GH1	GH	males	1.4891	0.5105	1.4299
6	GH11	GH	males	0.7552	0.4046	0.1706
7	GH13	GH	males			0.1894
8	GH15	GH	males	0.5517	0.338	1.8941
2	GH3	GH	males	0.4796	0.3104	0.7873
3	GH5	GH	males	1.6816	0.6981	0.3199
4	GH7	GH	males	0.7282	0.2356	0.6699
5	GH9	GH	males	0.7189	0.5888	1.9973

Fig4D **IL6 in WT KO mem fraction**

pairs	ID	Region	Housing	Geno	Frxn	nd Actin	L6/ Actin	no wt out
2	2.724000000000	GH WT M	GH	WT	M	0.86922	0.8484	0.8484
2	2.721000000000	GH KO M	GH	KO	M	1.08054	0.62353	0.62353
3	2.354000000000	SH KO M	SH	KO	M	0.45056		
3	2.746000000000	SH WT M	SH	WT	M	0.54625	1.71205	1.71205
4	2.750000000000	GH WT M	GH	WT	M	0.42663	1.58709	1.58709
4	2.749000000000	GH KO M	GH	KO	M	0.48644	0.86136	0.86136
5	2735*	SH WT M	SH	WT	M	1.2799	0.43213	0.43213
5	2729*	SH KO M	SH	KO	M	1.25598	0.35229	0.35229
6	2744*	SH WT M	SH	WT	M	1.66667	0.42034	0.42034
6	2747*	SH KO M	SH	KO	M	0.88517	0.60969	0.60969
7	2.779000000000	SH WT M	SH	WT	M	0.68239	1.54259	1.54259
7	2.725000000000	GH KO M	GH	KO	M	1.76074	1.00368	1.00368
8	2.692000000000	SH KO M	SH	KO	M	0.70767	0.62974	0.62974
8	2.763000000000	SH WT M	SH	WT	M	1.01516	0.63578	0.63578
9	2.778000000000	GH WT M	GH	WT	M	2.17776	0.81854	0.81854
9	2.781000000000	GH KO M	GH	KO	M	1.47852	0.54567	0.54567
10	2.693000000000	SH KO M	SH	KO	M	1.60067	0.56643	0.56643
10	2.759000000000	SH WT M	SH	WT	M	0.45072	1.09104	1.09104
11	2.739000000000	GH WT M	GH	WT	M	0.67397	0.91204	0.91204
11	2.738000000000	GH KO M	GH	KO	M	0.82561	1.4146	1.4146

Fig4E **Cyto IL6 in WT KO**

pairs	ID	Region	Housing	Geno	sex	Frxn	nd Actin	L6/ Actin	VAR10	no wt out	VAR12	VAR13	VAR14	VAR15	VAR16	VAR17	VAR18
1	2.708000000000	SH KO C	SH	KO	f	C	0.92626	1.77789		3.16593							
1	2.745000000000	SH WT C	SH	WT	f	C	1.11989	0.85779		1.52749							
2	2.724000000000	GH WT C	GH	WT	f	C	1.06755	1.04007		1.85209							
2	2.721000000000	GH KO C	GH	KO	f	C	1.30566	2.74231		4.88329							
3	2.354000000000	SH KO C	SH	KO	f	C	1.11727		8.95532		1.5946953811521						
3	2.746000000000	SH WT C	SH	WT	f	C	1.00737	0.34676		0.61749							
4	2.750000000000	GH WT C	GH	WT	f	C	0.84253	0.14807		0.26368							
4	2.749000000000	GH KO C	GH	KO	f	C	0.78235	1.30761		2.32849							
5	2735*	SH WT C	SH	WT	m	C	0.58873	0.57216		1.01886							
5	2729*	SH KO C	SH	KO	m	C	0.31399	0.31787		0.56603							
6	2744*	SH WT C	SH	WT	m	C	0.43173	0.40456		0.7204							
6	2747*	SH KO C	SH	KO	m	C	0.45266	1.40559		2.50297							
7	2.779000000000	SH WT C	SH	WT	f	C	1.65628	0.47454		0.88065							
7	2.725000000000	GH KO C	GH	KO	f	C	1.04139	0.83859		1.55627							
											2.722000000000	SH HT C	SH	HT	C	1.39986	0.17824
8	2.692000000000	SH KO C	SH	KO	f	C	1.28735	0.33919		0.62947							
8	2.763000000000	SH WT C	SH	WT	f	C	1.31613	0.64458		1.19622							
9	2.778000000000	GH WT C	GH	WT	f	C	1.09372		5.47519		outlier						
9	2.781000000000	GH KO C	GH	KO	f	C	0.8373	0.6705		1.24432							
											2657*	SH HT C	SH	HT	C	1.23501	0.17173
10	2.693000000000	SH KO C	SH	KO	f	C	1.14343	0.17457		0.32397							
10	2.759000000000	SH WT C	SH	WT	f	C	1.16698	0.13898		0.25792							
11	2.739000000000	GH WT C	GH	WT	f	C	0.70909	0.8973		1.66521							
11	2.738000000000	GH KO C	GH	KO	f	C	0.86608	0.72024		1.33663							

Fig4H 4I microglia

pairs	id	geno	#MG	Area	Int dens	VAR7	VAR8	VAR9	VAR10	VAR11
1	B11 S19 B2 KRM	wt	61	0.00159	0.05641					
2	B11 S19 B3 KRM	ko	53	0.00168	0.0597					
2	B11 S19 B4 KRM	wt	36	0.00154	0.04623					
3	B12 s18 B 1 KRM	ko	49	0.00283	0.05492					
4	B12 s18 b2 KRM	wt	32	0.0019	0.04975					
3	B12 s 20 B3 KRM	wt	35	0.00216	0.04965					
4	B12 s 20 B 4 KR	ko	67	0.00225	0.07741					
						B13 s 20 B1 KRM	ko	33	0.0028	0.06637
6	B13 s20 B2 KRM	wt	43	0.00246	0.05213					
6	B13 s 20 B3 KRM	ko	82	0.00218	0.06533					
1	B11 S19 b1 KRM	ko	71	0.00195	0.06038					

Fig4K and 4L Astrocytes

Section	Block	ID #	Genotype	Age	Sex	strocytes	Avg. ROD	ROD - BG	cell body	processes
070618 C3C6-1 GFAP B4	1	1577	WT	Young	Male	52	6.8255	6.7827	0.043	3.6
070618 C3C6-3 GFAP B6	3	1446	WT	Young	Male	20	5.4788	5.4708	0.0358	3.2
070618 C3C6-6 GFAP B3	6	1148	WT	Young	Male	18	6.0349	6.0309	0.0397	3.5
070618 C3C6-9 GFAP B2	9	1453	WT	Young	Male	31	5.1688	5.1658	0.0343	4.1
070618 C3C6-2 GFAP B5	2	1544	WT	Young	Female	35	4.0567	4.0317	0.0254	3.1
070618 C3C6-4 GFAP B1	4	1387	WT	Young	Female	15	4.3057	4.2947	0.0258	3.1
070618 C3C6-5 GFAP B5	5	1385	WT	Young	Female	20	4.1159	4.1129	0.0298	2.6
070618 C3C6-7 GFAP B1	7	1553	WT	Young	Female	24	4.7331	4.6901	0.0321	3.9
070618 C3C6-8 GFAP B4	8	1322	WT	Young	Female	35	3.1023	3.0823	0.0232	3.8
070618 C3C6-1 GFAP B6	1	1550	KO	Young	Male	44	4.3837	4.3664	0.0306	4.1
070618 C3C6-3 GFAP B5	3	1445	KO	Young	Male	22	4.7238	4.7198	0.0291	3.2
070618 C3C6-6 GFAP B1	6	1326	KO	Young	Male	17	5.9613	5.9483	0.0402	3.5
070618 C3C6-9 GFAP B6	9	1444	KO	Young	Male	41	3.0808	3.0658	0.0241	3
070618 C3C6-2 GFAP B4	2	1548	KO	Young	Female	34	5.6708	5.6617	0.0365	2.9
070618 C3C6-4 GFAP B3	4	1392	KO	Young	Female	16	4.1267	4.0697	0.0271	3.4
070618 C3C6-5 GFAP B3	5	1452	KO	Young	Female	22	4.3775	4.3525	0.0296	4
070618 C3C6-7 GFAP B5	7	1556	KO	Young	Female	20	3.6221	3.6191	0.0263	3.8
070618 C3C6-8 GFAP B2	8	1575	KO	Young	Female	26	5.2793	5.2673	0.0342	4
070618 C3C6-1 G	1	1551	HT	Young	Male	32	5.8392	5.7699	0.0363	3.1
070618 C3C6-3 G	3	1443	HT	Young	Male	25	5.1928	5.1868	0.0335	3.4
070618 C3C6-6 G	6	1547	HT	Young	Male	26	4.9073	4.8933	0.034	4.1
070618 C3C6-9 G	9	1450	HT	Young	Male	19	4.2874	4.2674	0.0277	3.5
070618 C3C6-2 G	2	1546	HT	Young	Female	30	5.6689	5.6429	0.0355	4.5
070618 C3C6-4 G	4	1391	HT	Young	Female	28	4.7046	4.6626	0.0271	4.1
070618 C3C6-5 G	5	1447	HT	Young	Female	22	5.4119	5.4029	0.0313	3.8
070618 C3C6-7 G	7	1552	HT	Young	Female	30	4.4466	4.4346	0.0336	4.5
070618 C3C6-8 G	8	1557	HT	Young	Female	52	4.1275	4.1205	0.0295	3.8
