# Supplementary material for: The Role of PDE11A4 in Social Isolation-Induced Changes in Intracellular Signaling and Neuroinflammation
Source: Front Pharmacol. 2021 Nov 23;12:749628. doi: 10.3389/fphar.2021.749628 (PMC8650591; doi:10.3389/fphar.2021.749628)
Supplement: Supplementary file 6 [file DataSheet1.PDF]

Fig1A VHM Actin

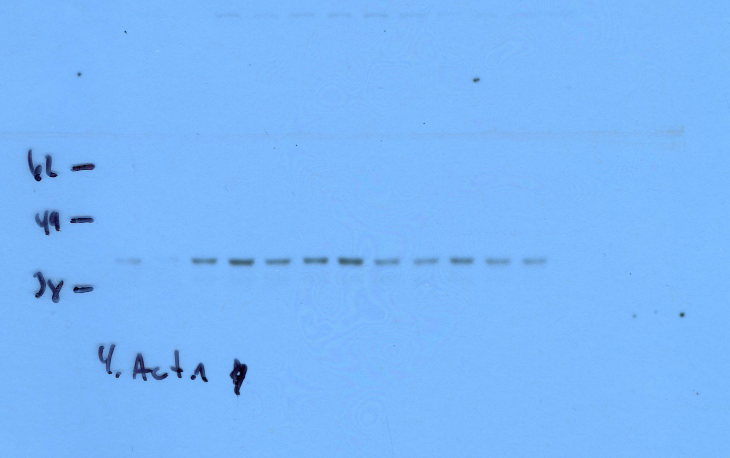

Fig1A VHM PDE11

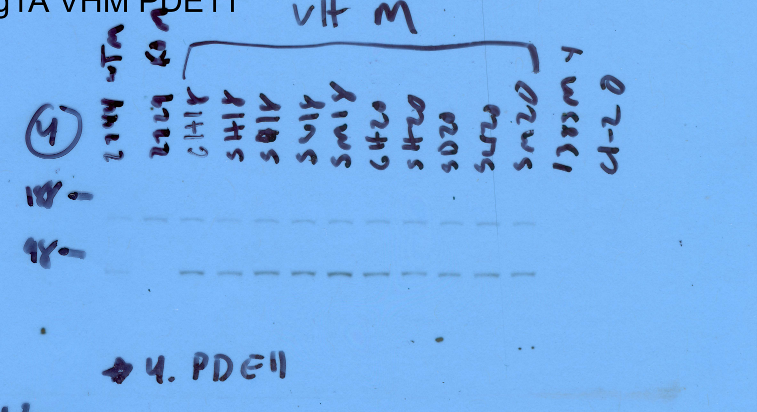

Fig2B VHC Actin

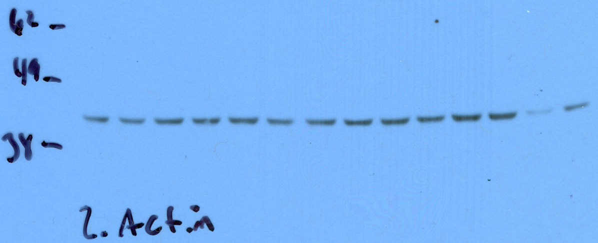

Fig2B VHC PDE11

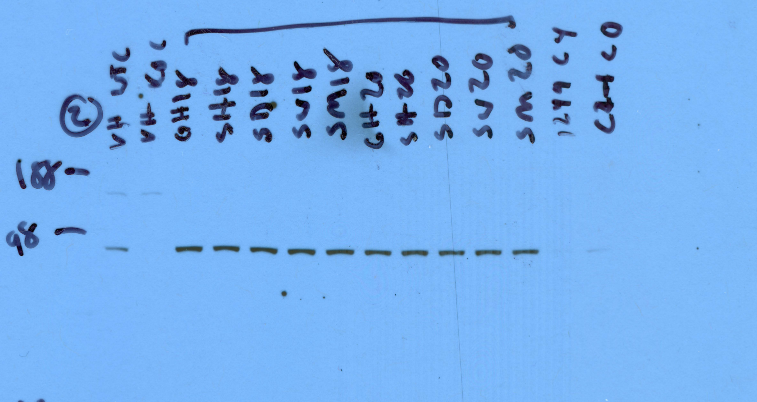

Fig1C VHN Actin

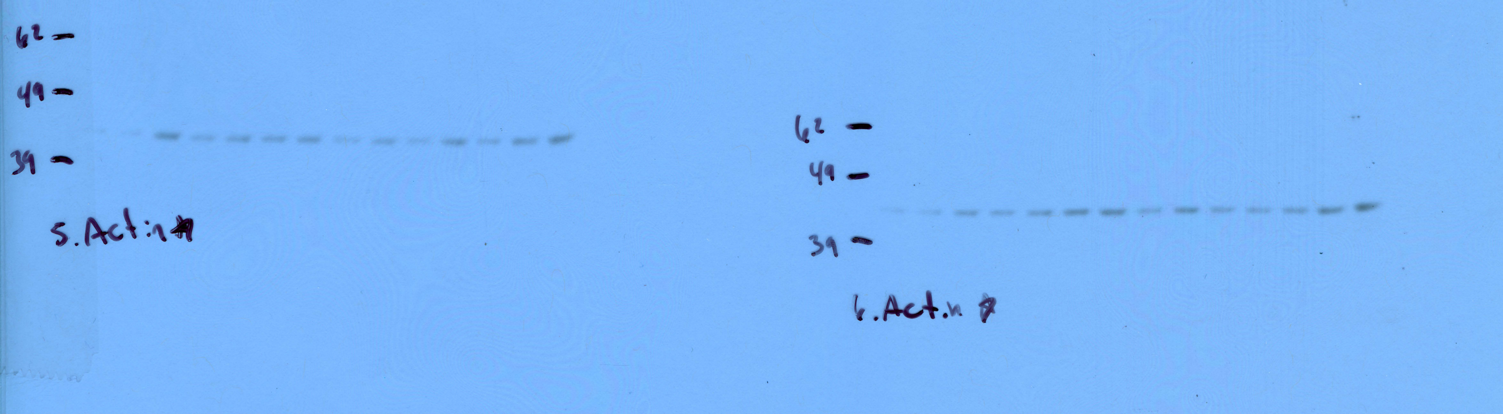

Fig1C VHN PDE11

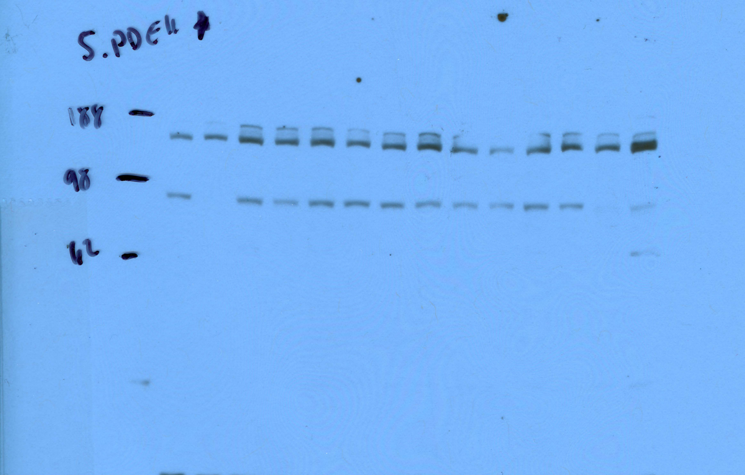

Fig1D DHM Actin

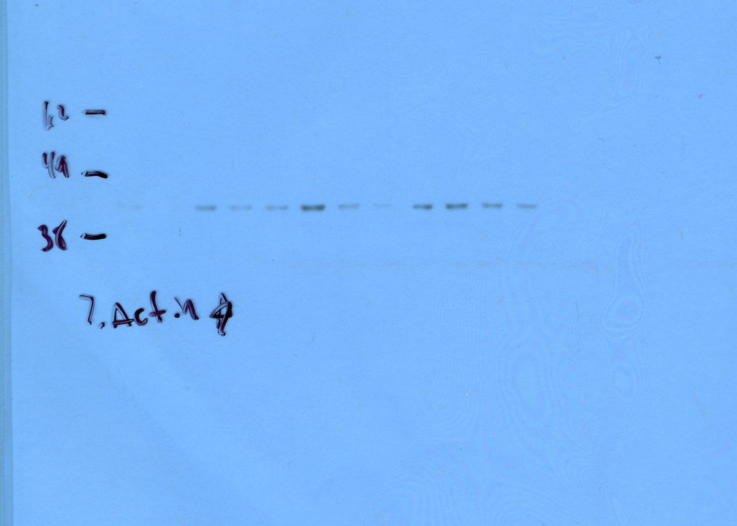

Fig1D DHM PDE11

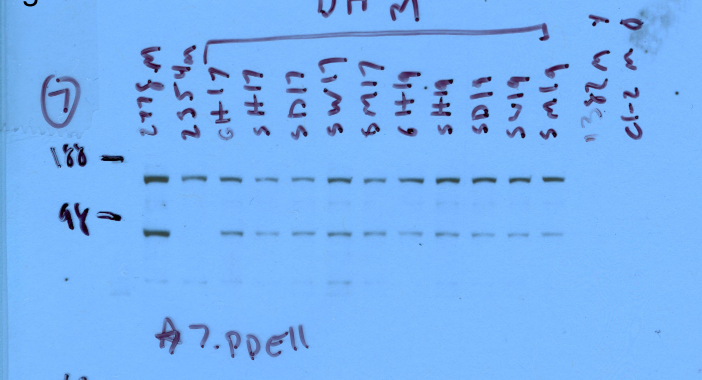

Fig1E DHC Actin

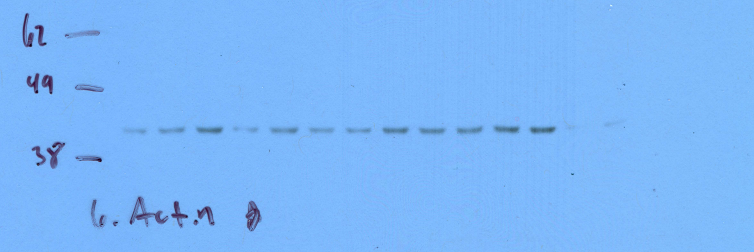

Fig1E DHC PDE11

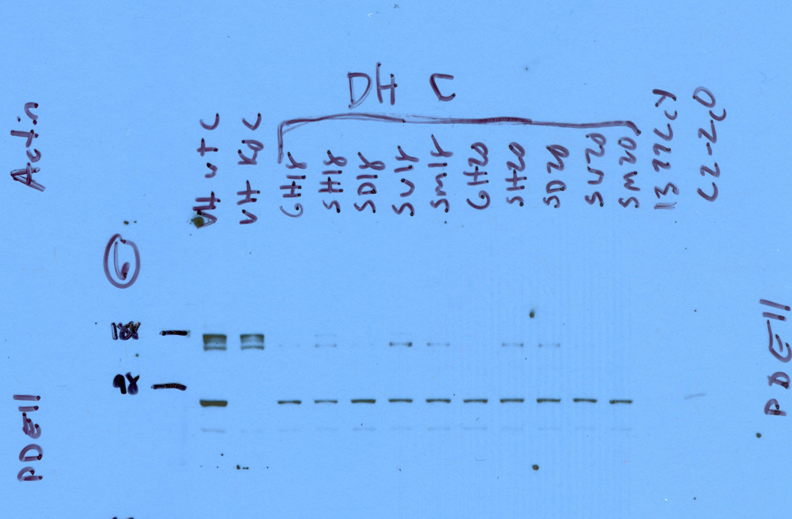

Fig1F DHN Actin

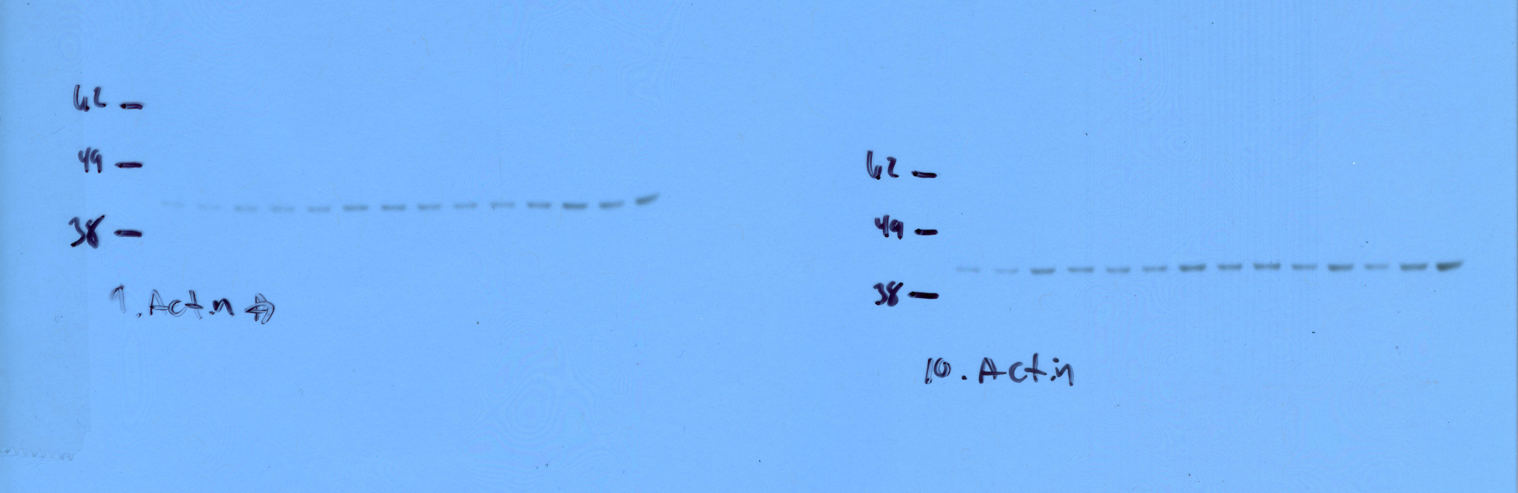

Fig1F DHN PDE11

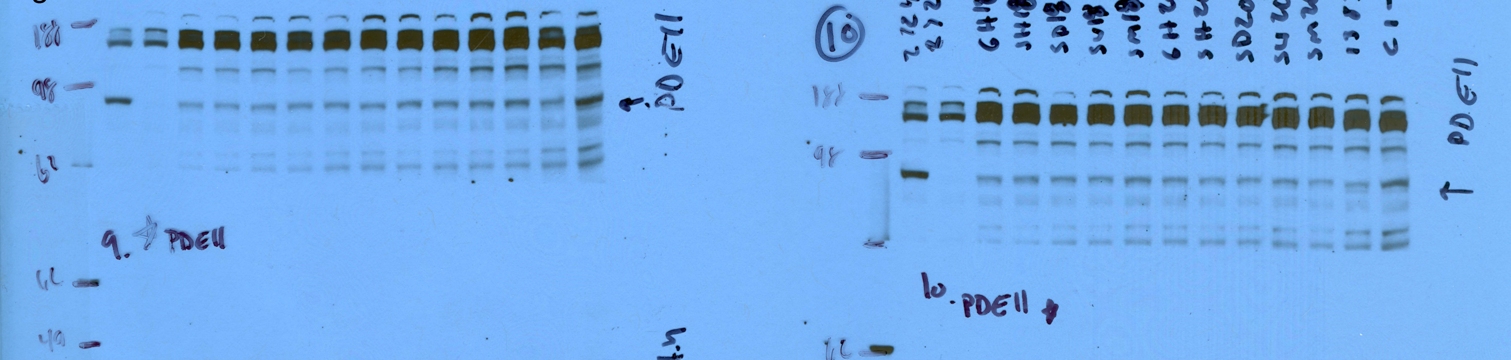

Fig1G VHM PDE10

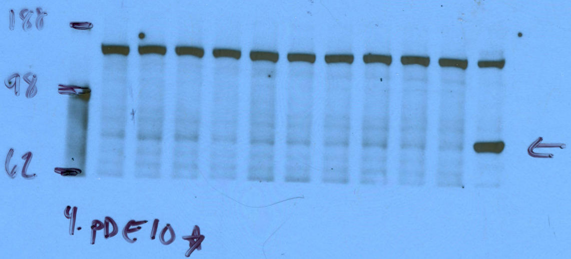

Fig1G VHM PDE2

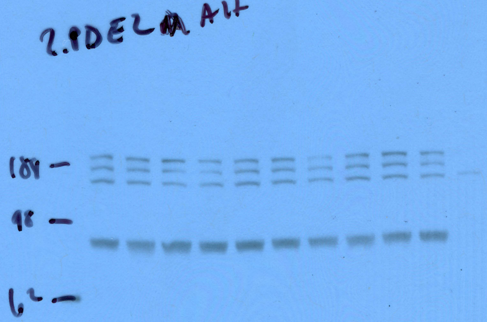

Fig2A VHM Actin

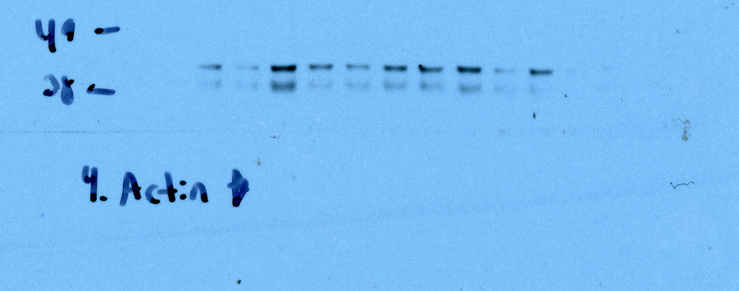

Fig2A VHM PDE11

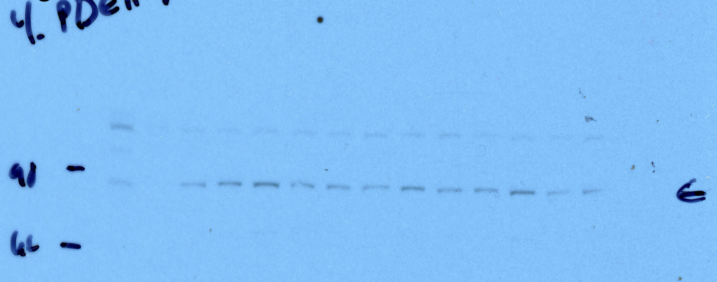

Fig2A WT KO Actin

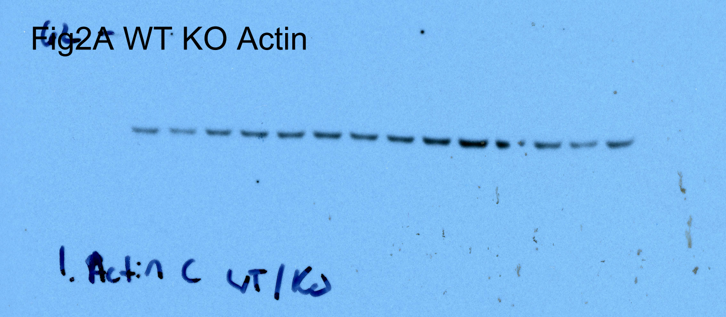

Fig2A WT KO PDE11

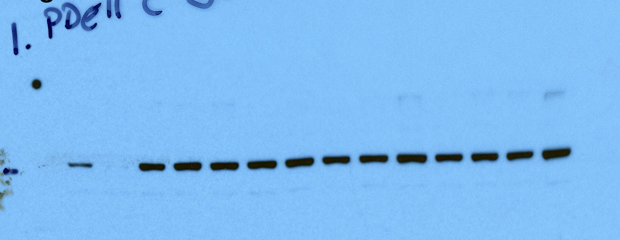

Fig2B VHC Actin

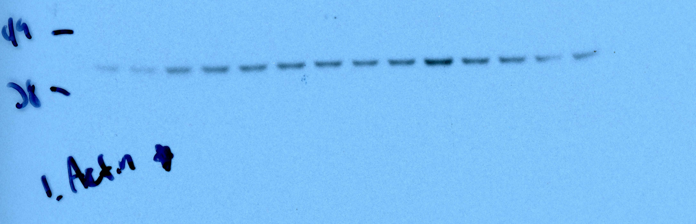

Fig2B VHC PDE11

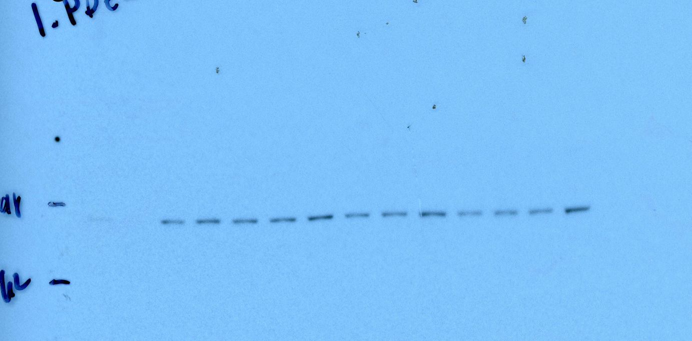

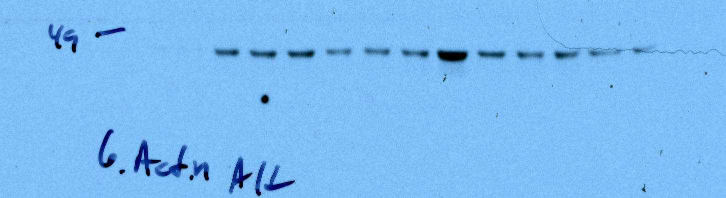

Fig2C VHN Actin

Fig2C VHN PDE11

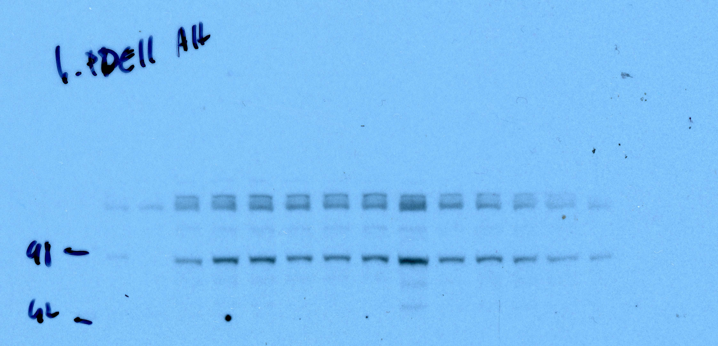

Fig3A VHT S6

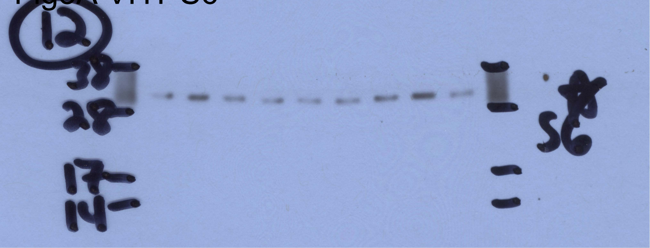

Fig3A VHT pS6 235

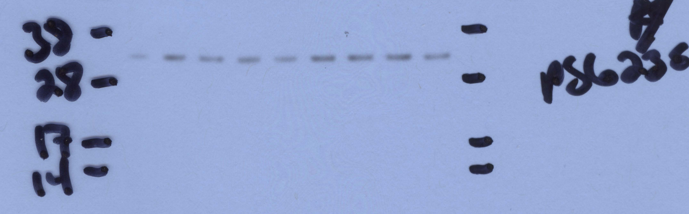

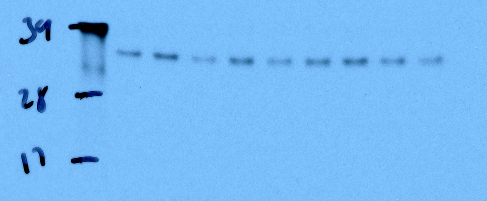

Fig3A VHT pS6 240

4. pS6 240/244

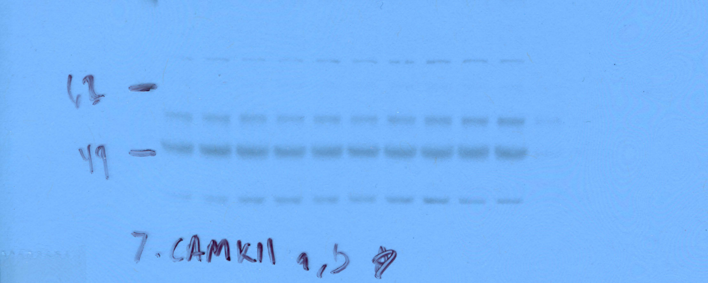

Fig3A VHT CAMKII

Fig3A VHT pCAMKII

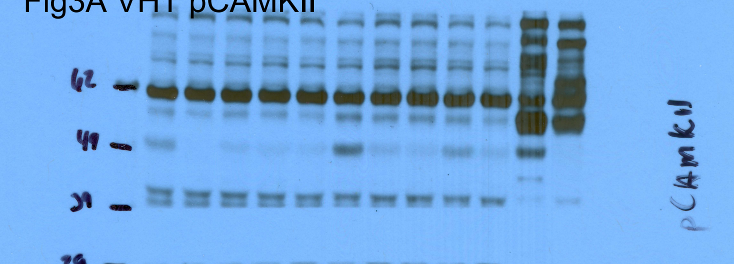

Fig4A VHM Actin

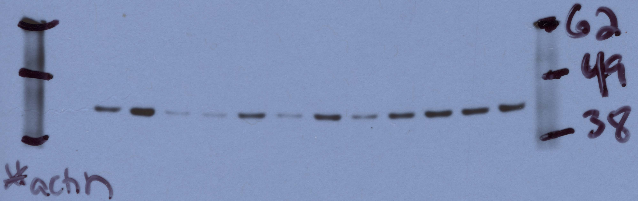

Fig4A VHM IL6 Male

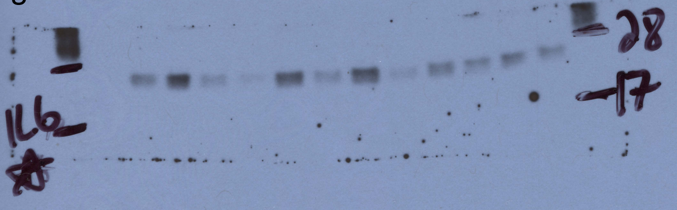

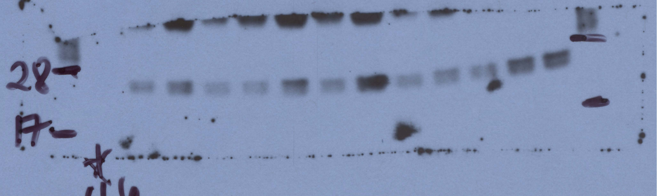

Fig4A VHM IL6 Female

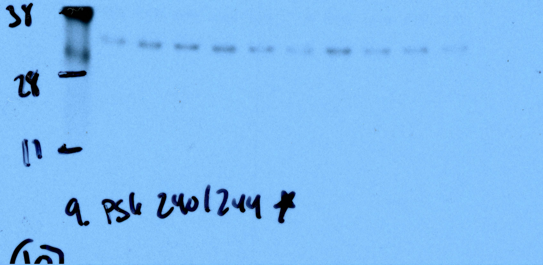
38
28
17
9. PS6 240/244 *

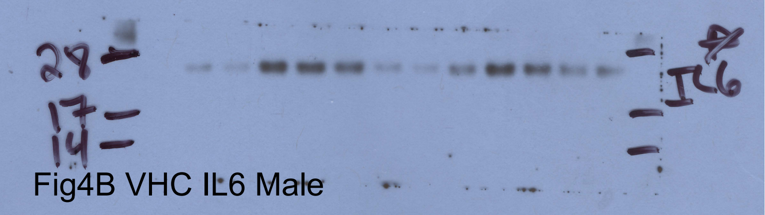

Fig4B VHC IL6 Male

Fig4B VHC Actin Male

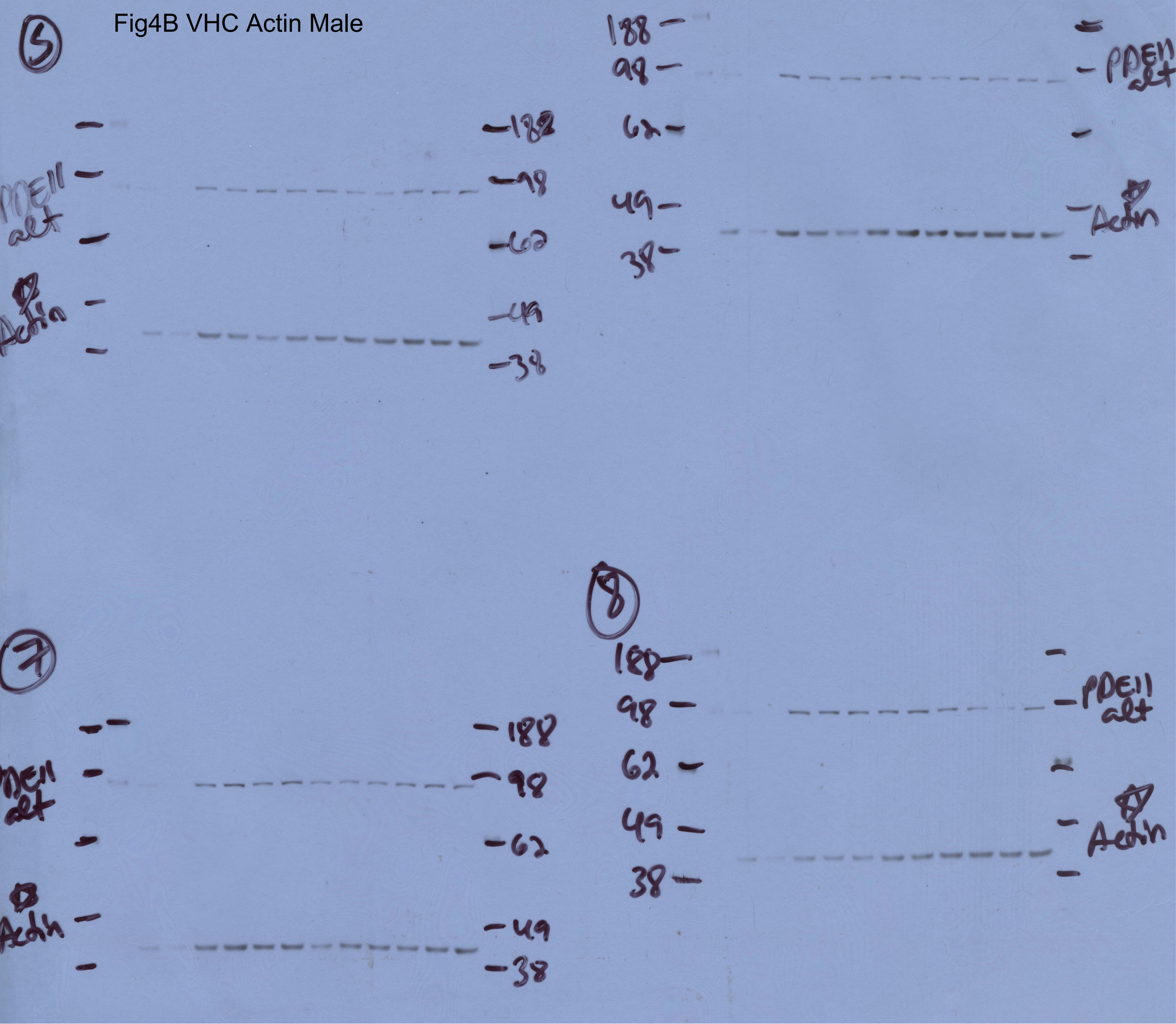

Fig4B VHC Actin Female

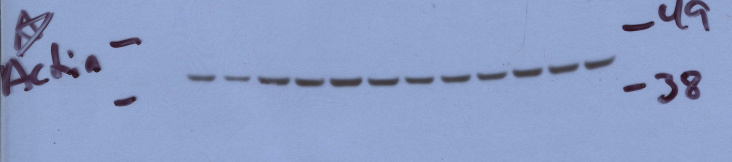

Fig4B VHC IL6 Female

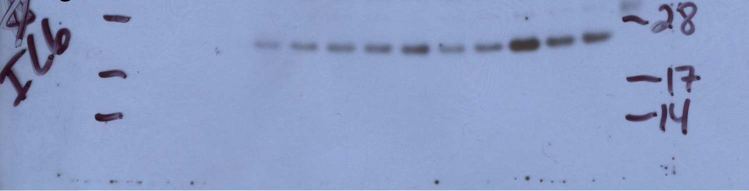

Fig4D WTKO VHM Actin

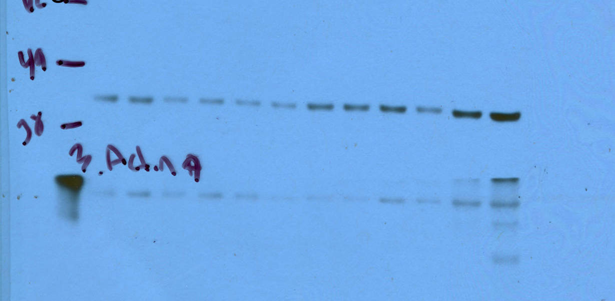

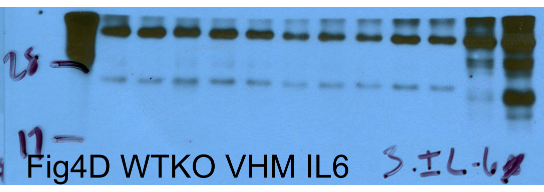

Fig4D WTKO VHM IL6

Fig4E WTKO VHC Actin

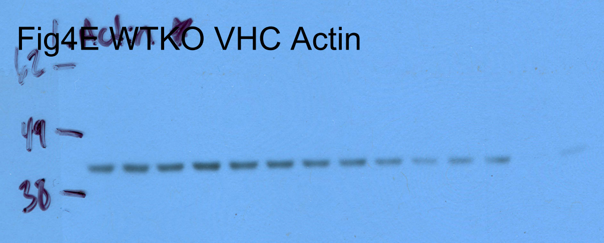

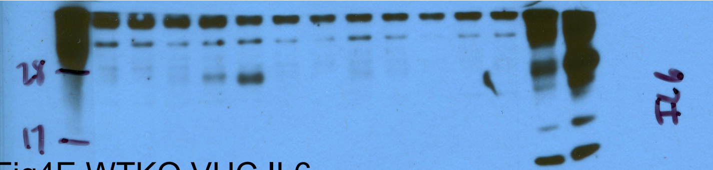

Fig4E WTKO VHC IL6

Fig4G WT IBA1

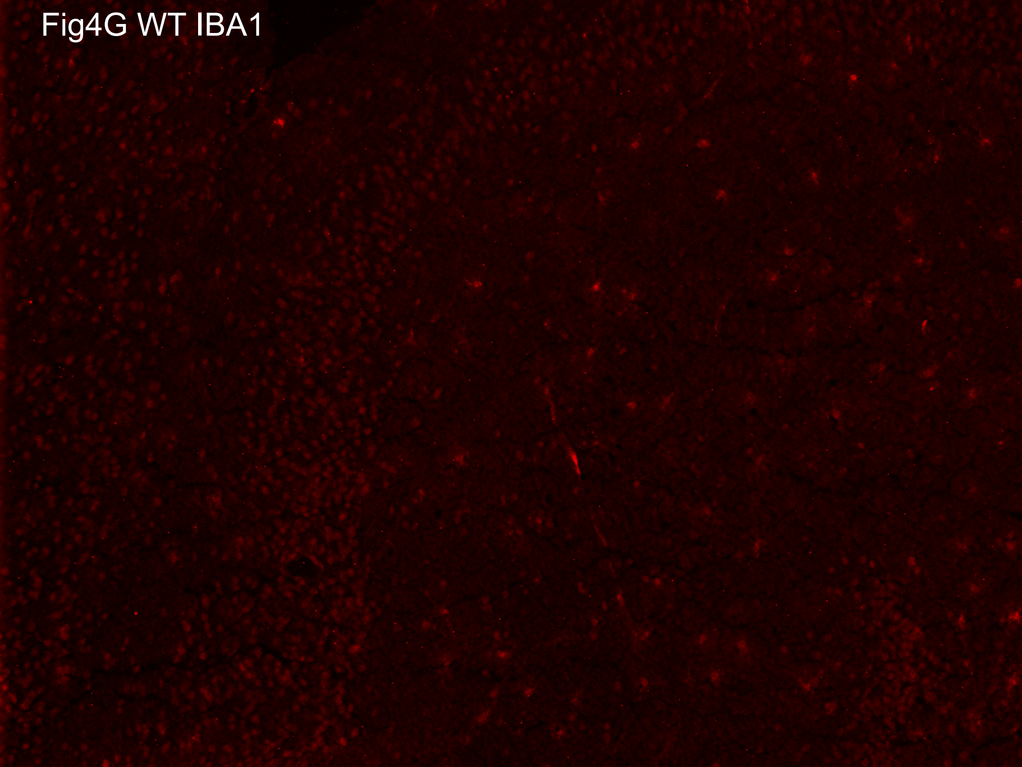

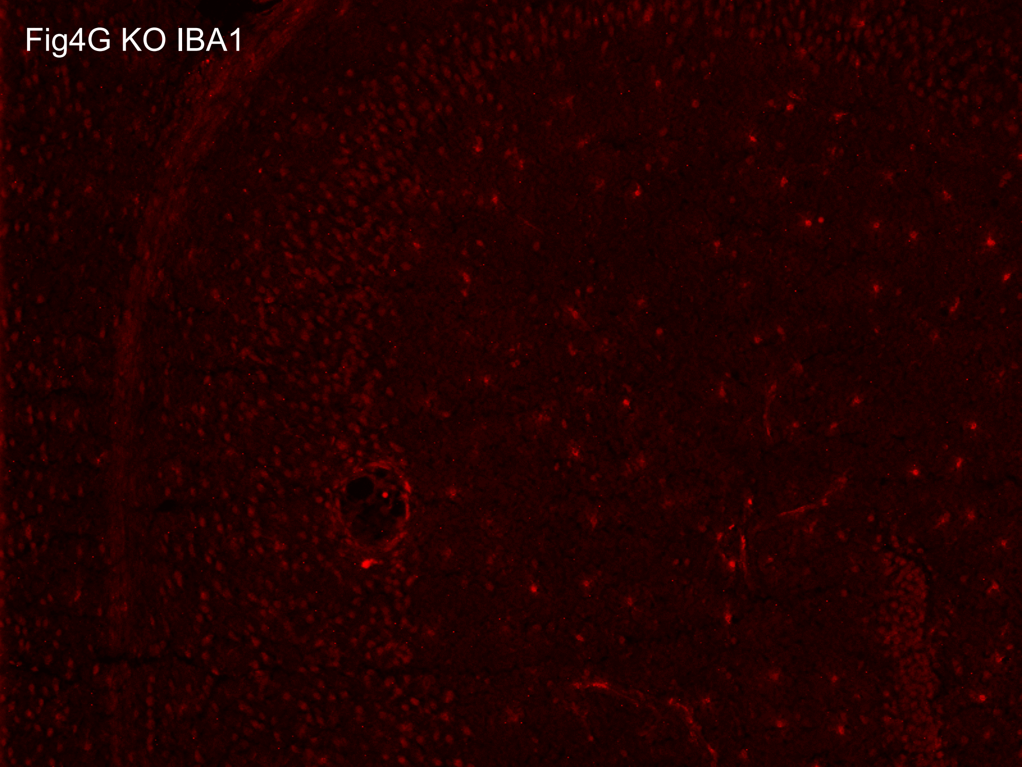
Fig4G KO IBA1

Fig4J WT GFAP

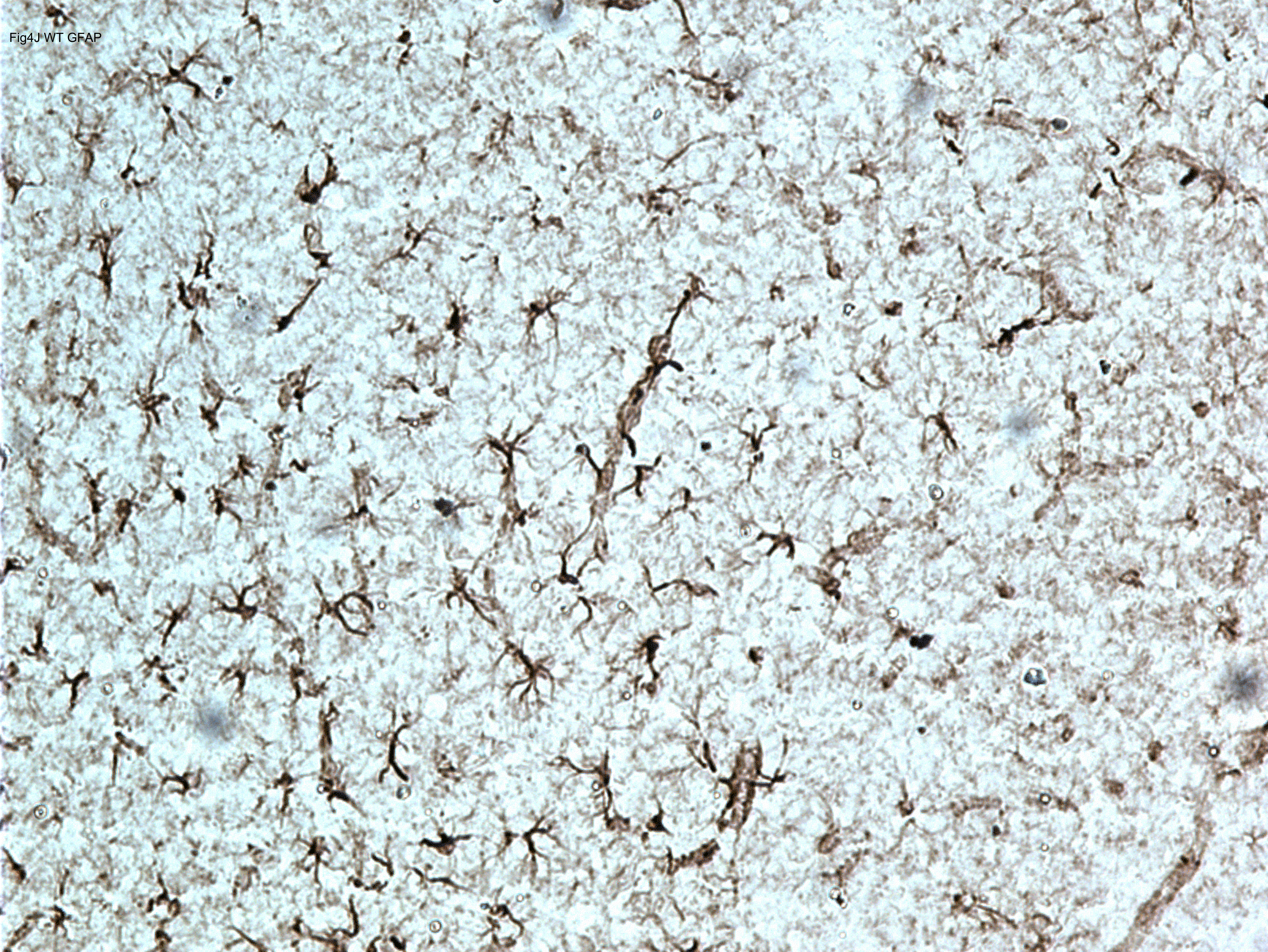

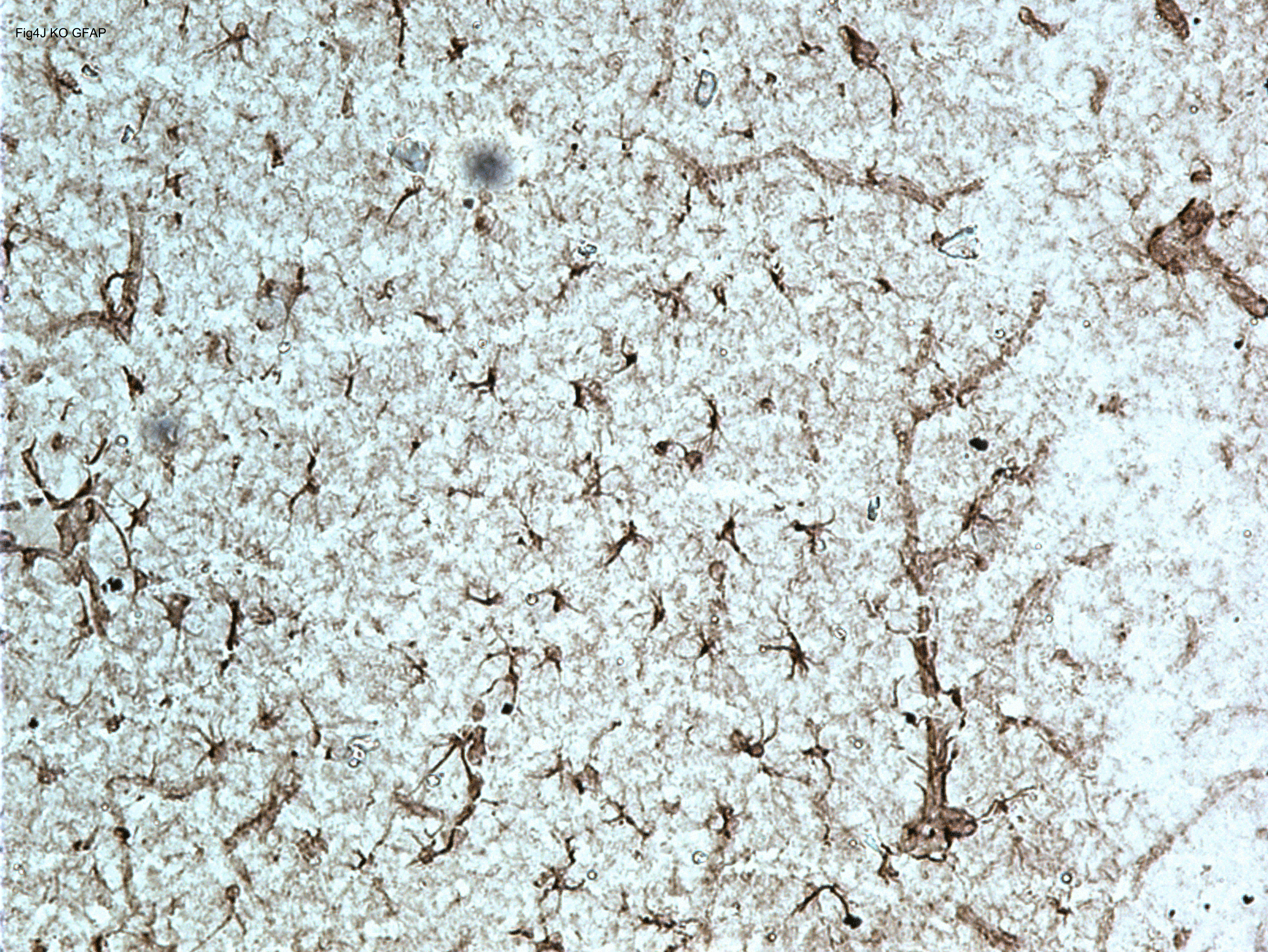
Fig4J KO GFAP
